# Supplementary material for: Biochemical and neurophysiological effects of deficiency of the mitochondrial import protein TIMM50
Source: eLife. 2024 Dec 16;13:RP99914. doi: 10.7554/eLife.99914 (PMC11649234; doi:10.7554/eLife.99914)

TIMM50

(The running order is H.C / P1 / P2)

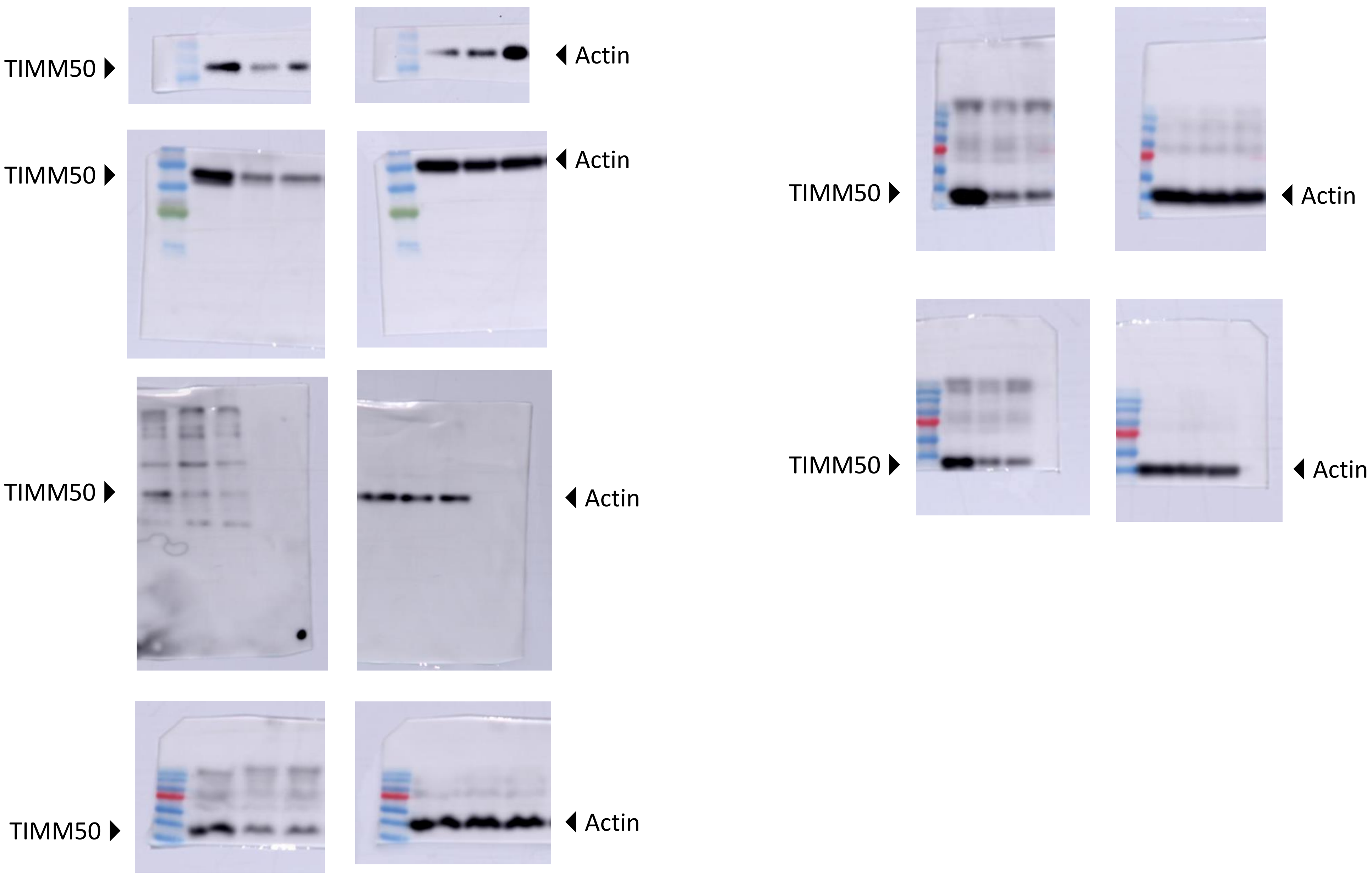

TIMM23

(The running order is H.C / P1 / P2)

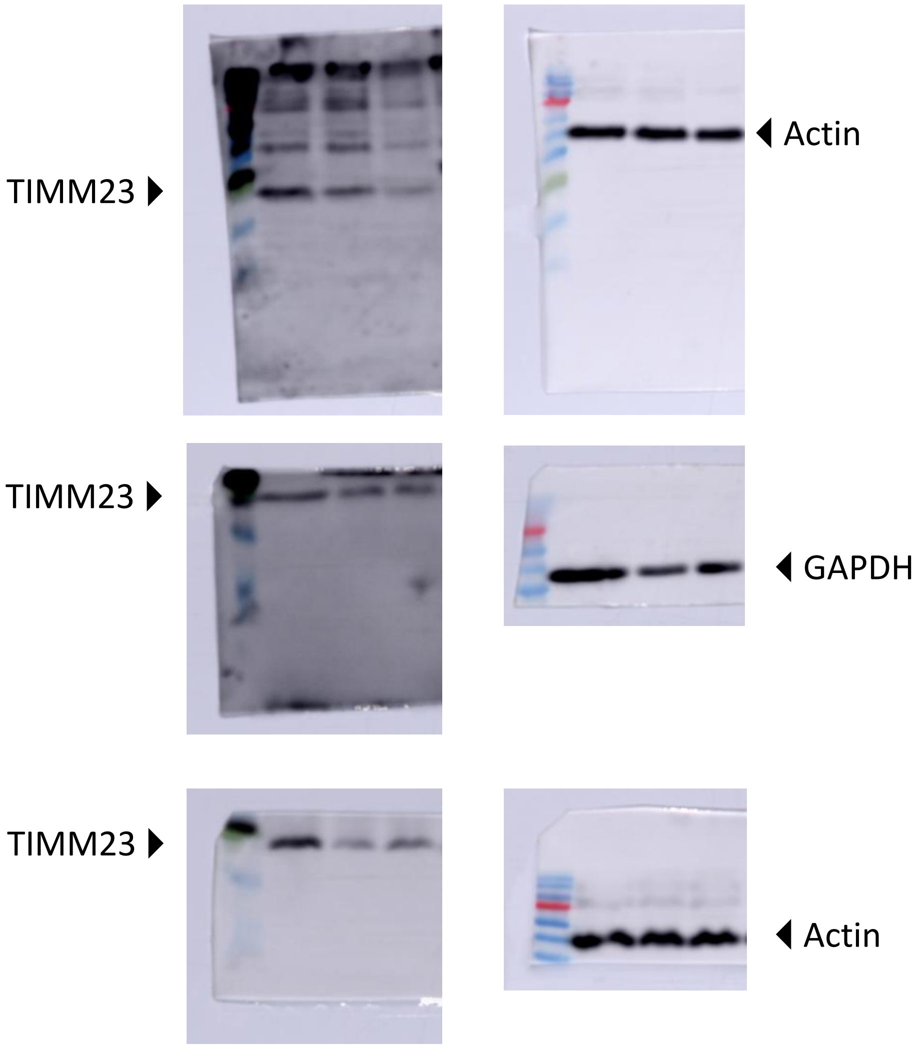

TIMM17A

(The running order is H.C / P1 / P2)

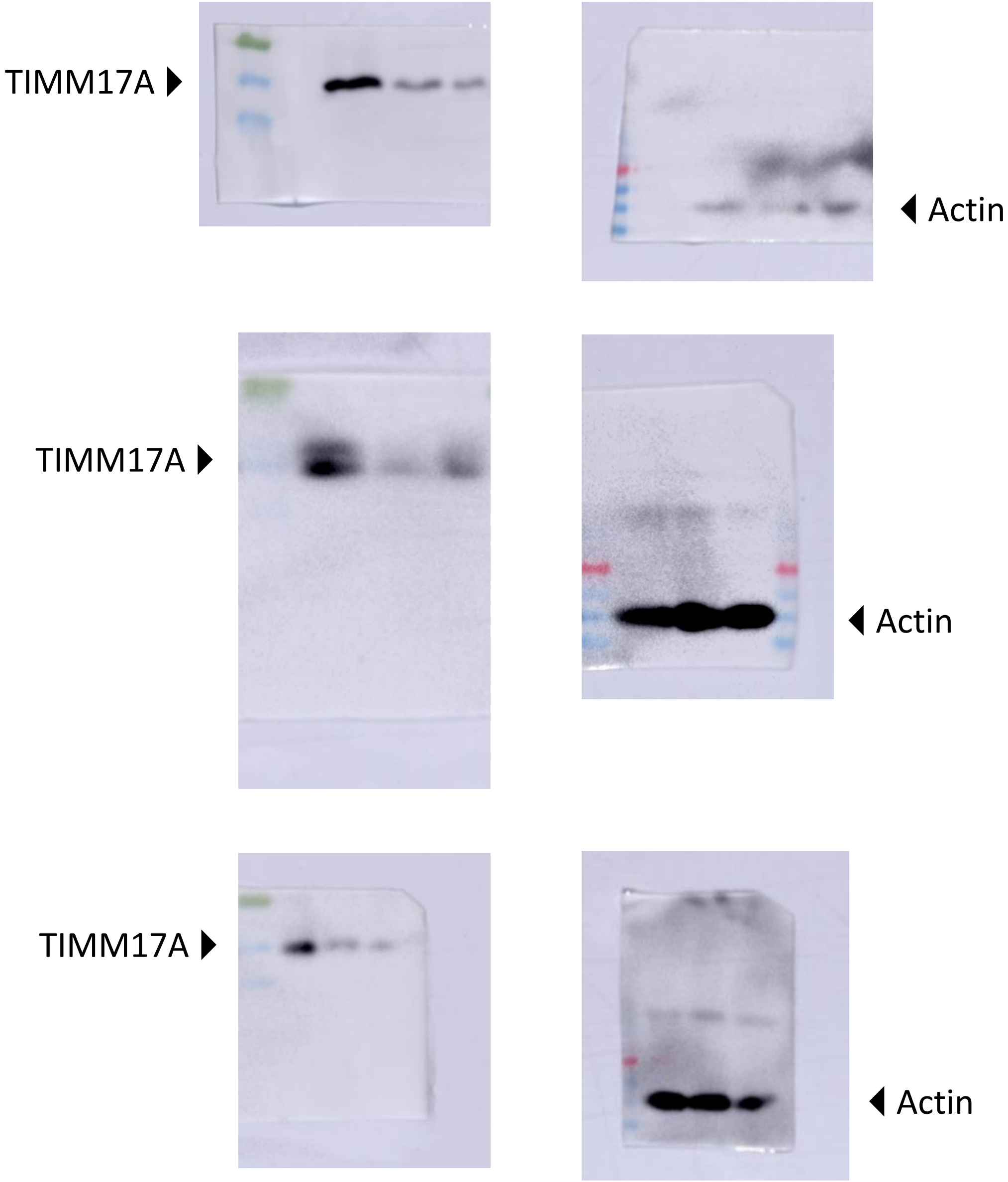

TIMM17B

(The running order is H.C / P1 / P2)

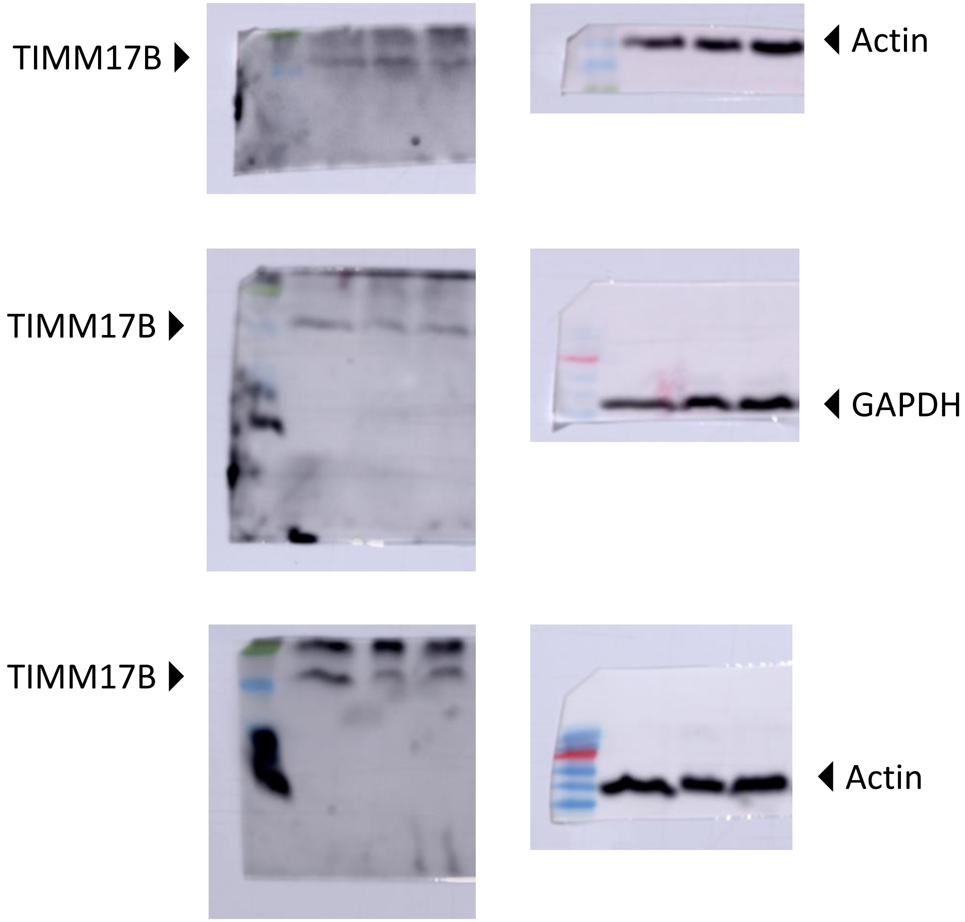

TIMM21

(The running order is H.C / P1 / P2)

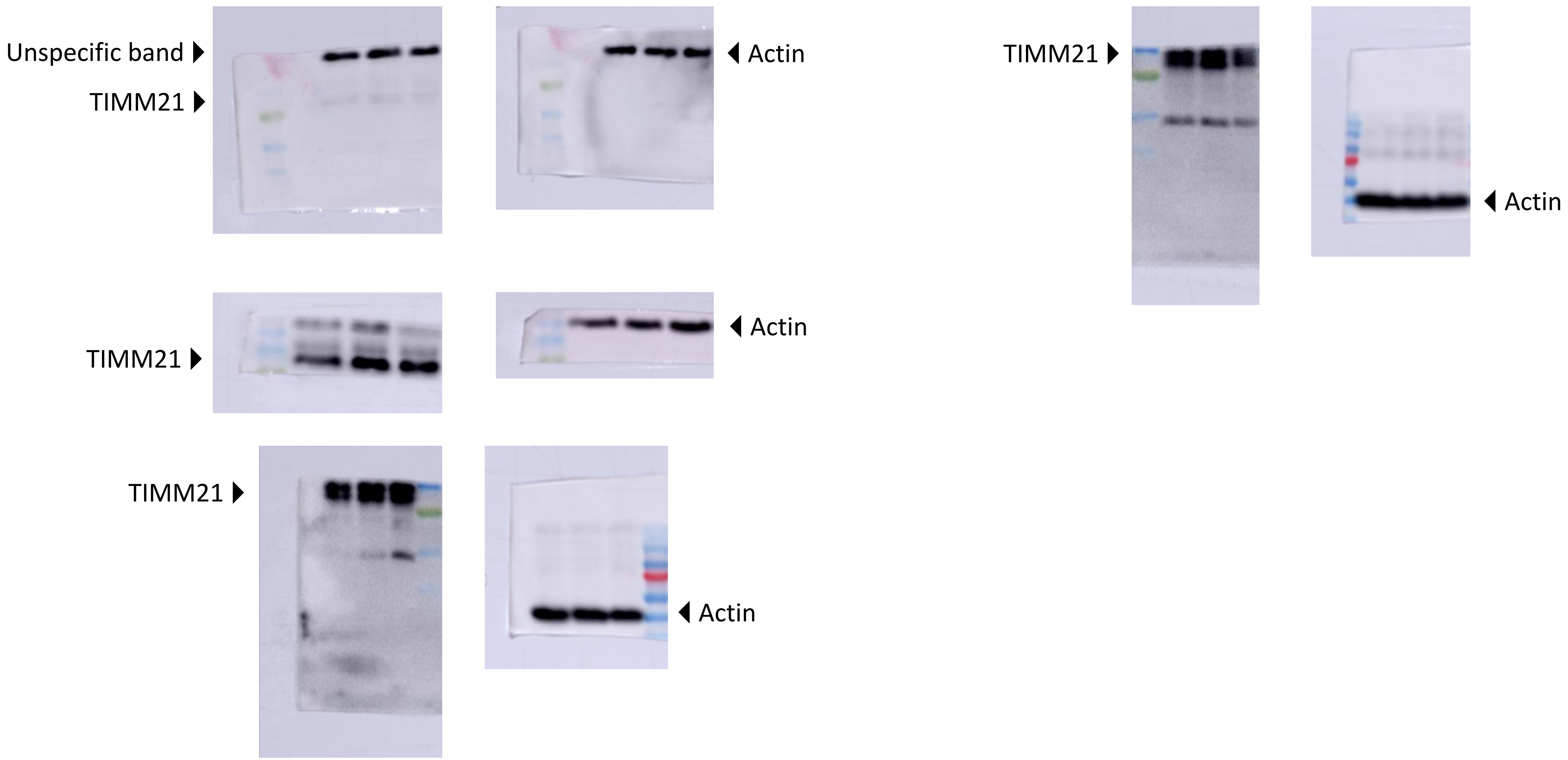

TIMM44

(The running order is H.C / P1 / P2)

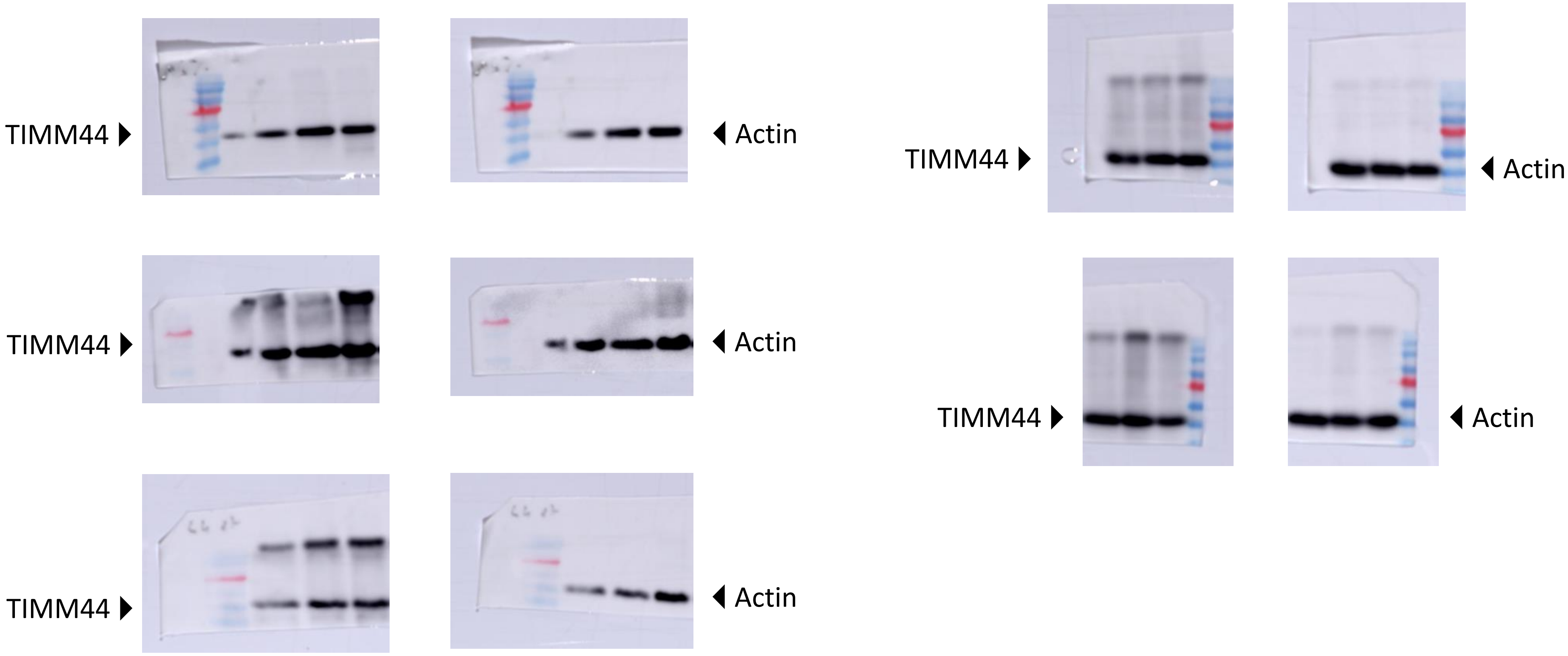

Pam16

(The running order is H.C / P1 / P2)

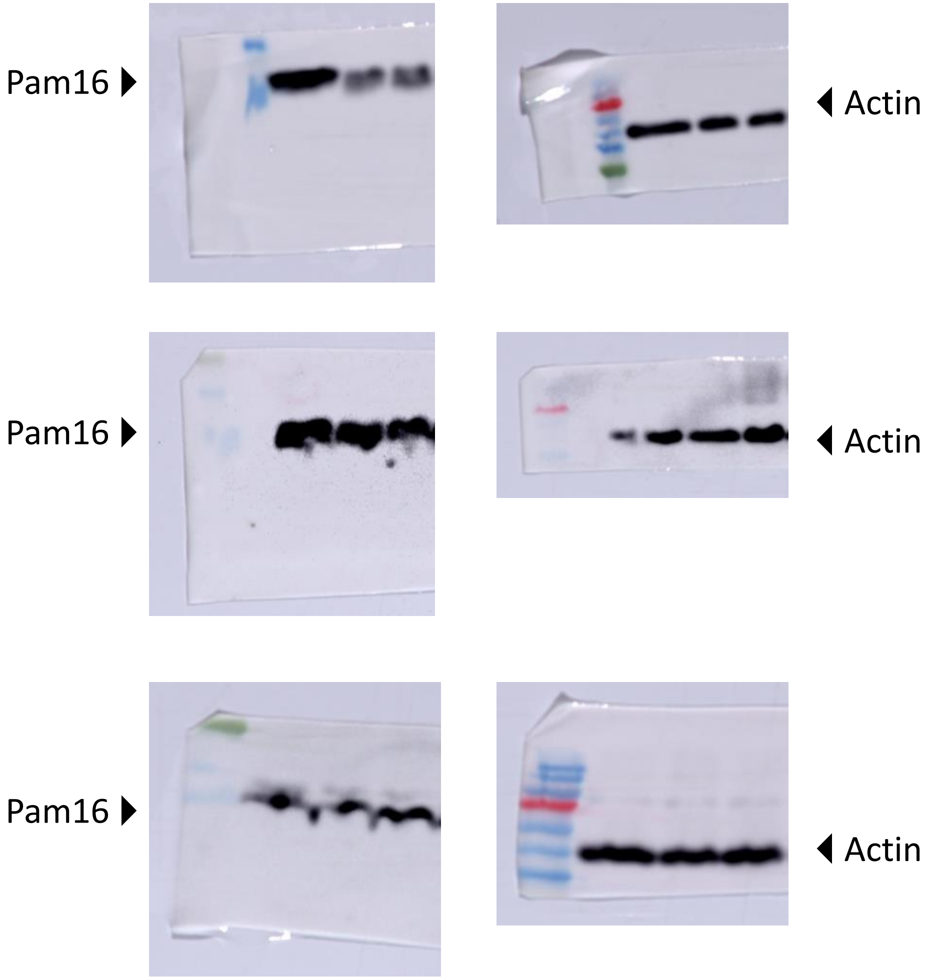

TOMM40

(The running order is H.C / P1 / P2)

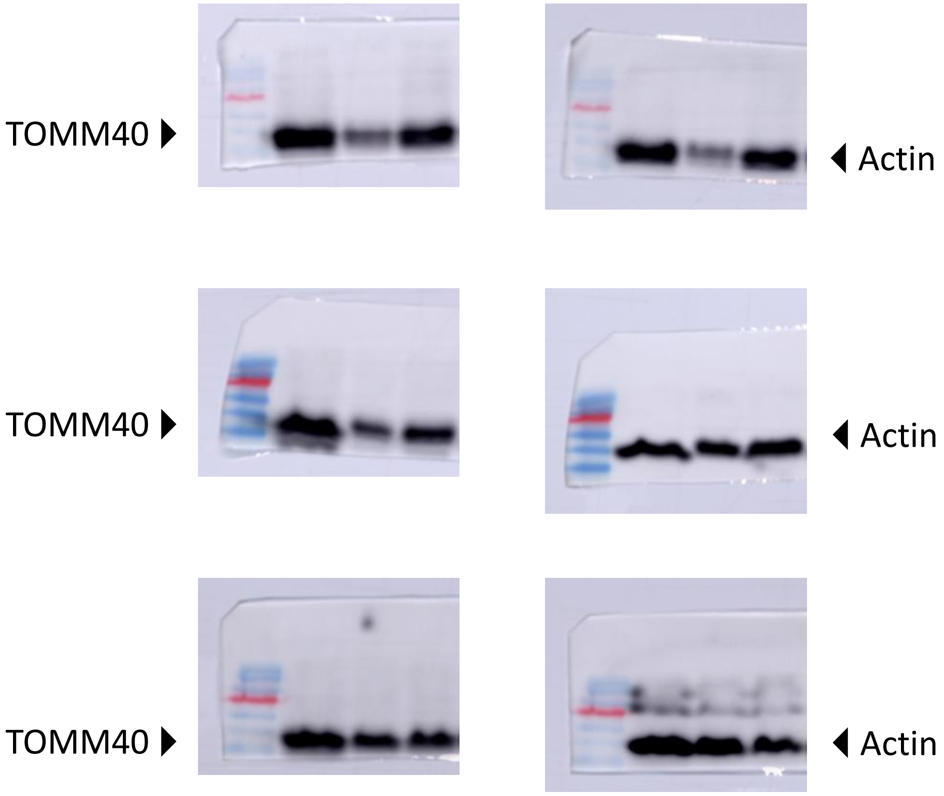

TOMM20

(The running order is H.C / P1 / P2)

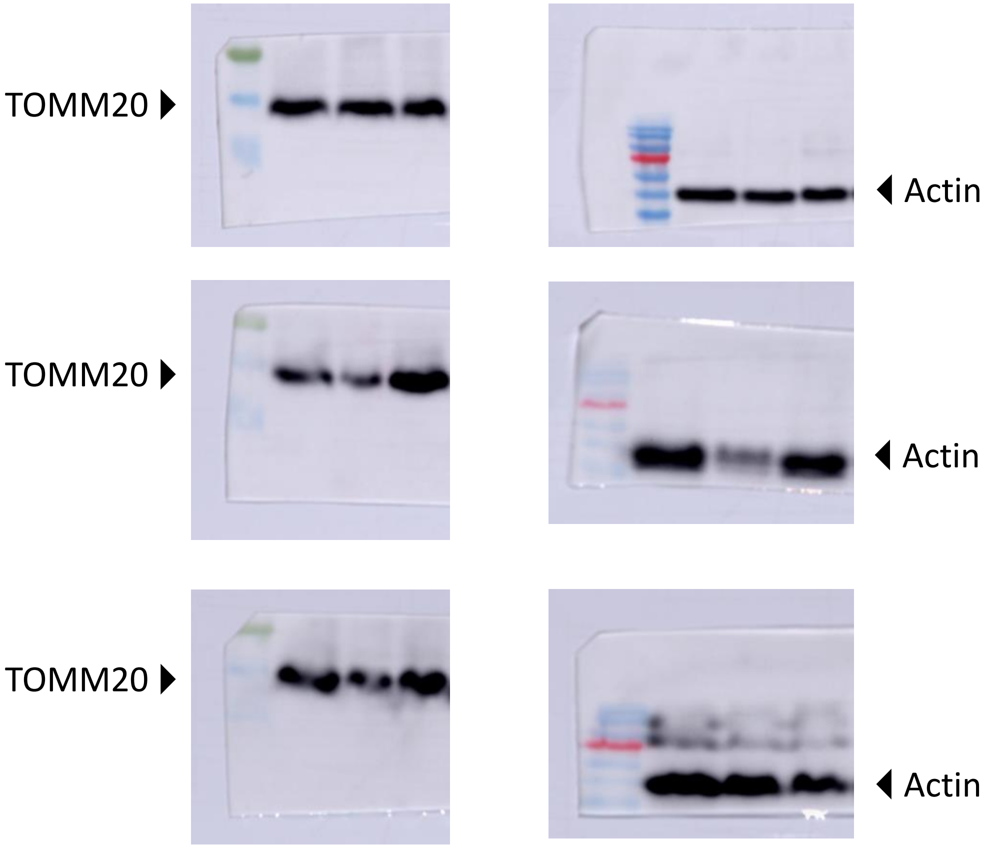

mtHsp60

(The running order is H.C / P1 / P2)

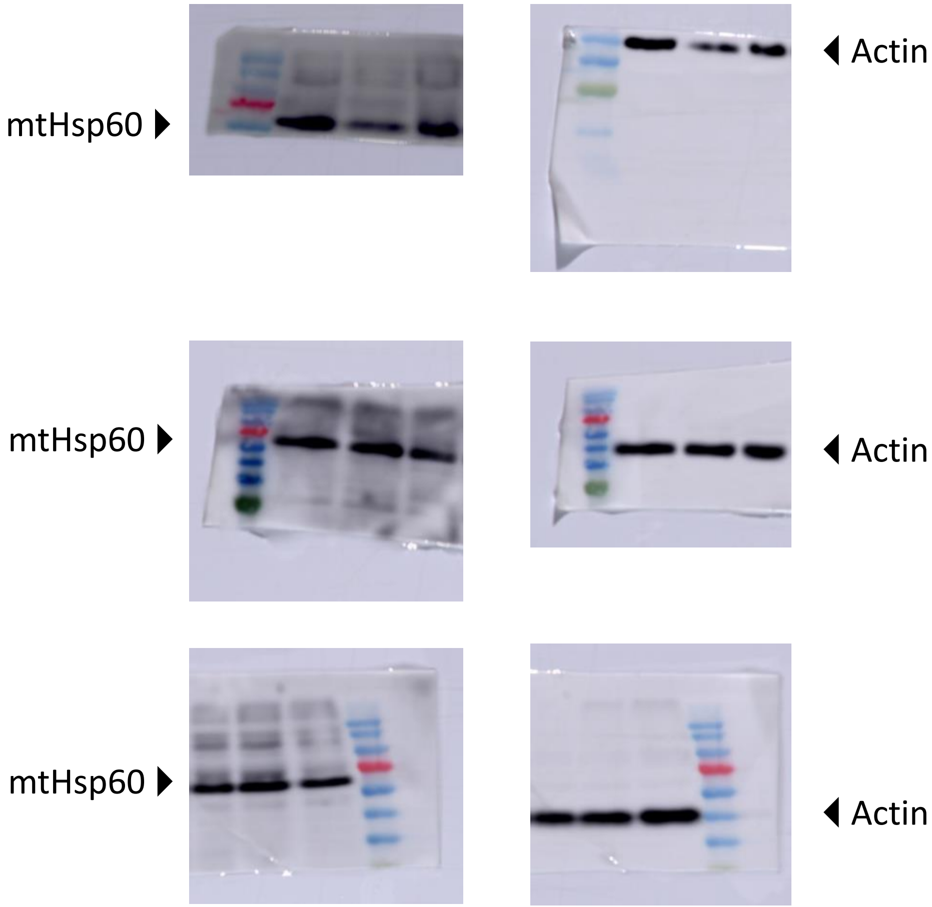

Aconitase-2

(The running order is H.C / P1 / P2)

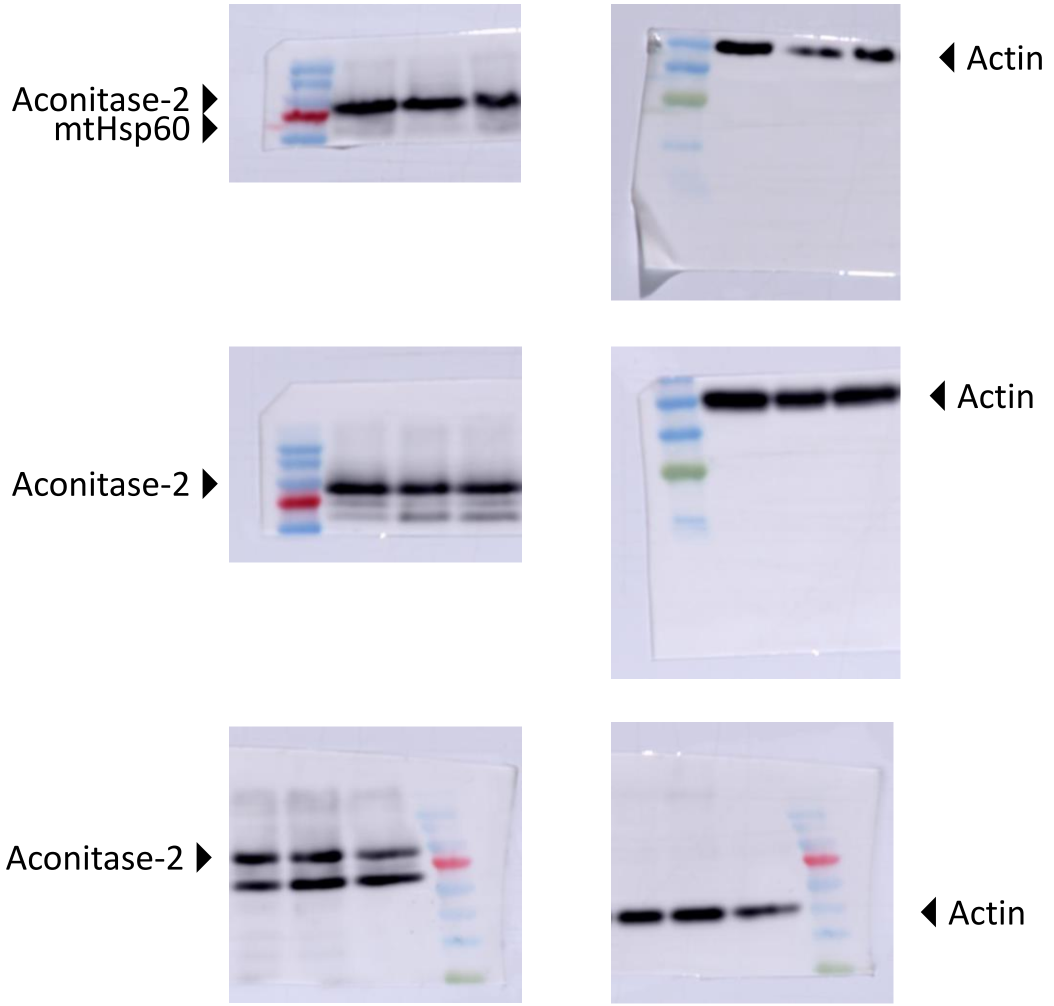

Supplement: Figure 1—source data 1. [file elife-99914-fig1-data1.zip › Figure 1-source data 1.pdf]
