# Supplementary figures and images for: Biochemical and neurophysiological effects of deficiency of the mitochondrial import protein TIMM50 (part 1 of 2)
Source: eLife. 2024 Dec 16;13:RP99914. doi: 10.7554/eLife.99914 (PMC11649234; doi:10.7554/eLife.99914)

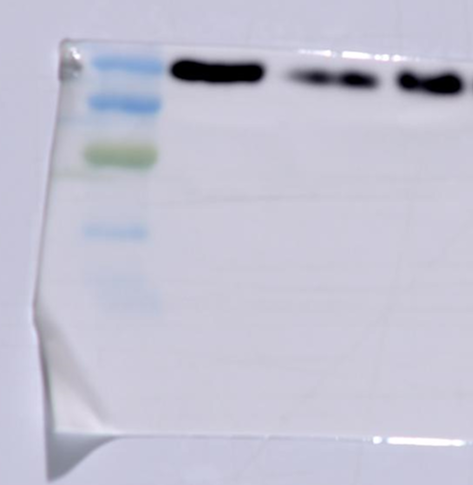

Supplement: Figure 1—source data 2. [file elife-99914-fig1-data2.zip › Figure 1-source data 2/Aconitase-2 Actin rep1.tif]

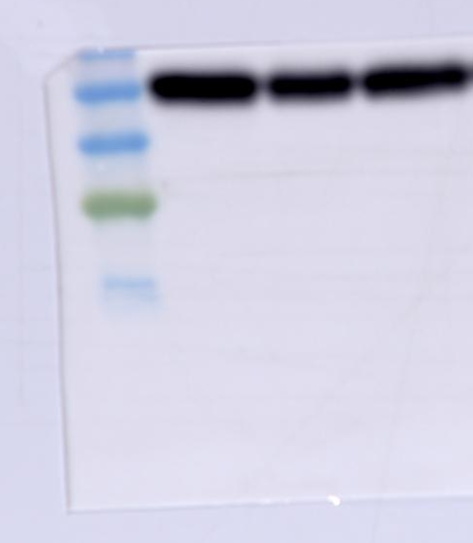

Supplement: Figure 1—source data 2. [file elife-99914-fig1-data2.zip › Figure 1-source data 2/Aconitase-2 Actin rep2.tif]

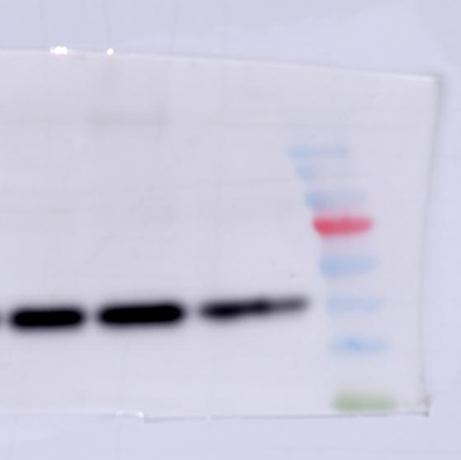

Supplement: Figure 1—source data 2. [file elife-99914-fig1-data2.zip › Figure 1-source data 2/Aconitase-2 Actin rep3.tif]

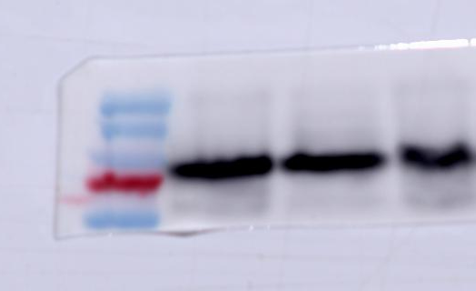

Supplement: Figure 1—source data 2. [file elife-99914-fig1-data2.zip › Figure 1-source data 2/Aconitase-2 rep1.tif]

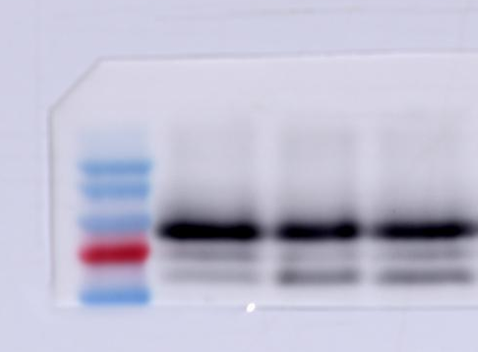

Supplement: Figure 1—source data 2. [file elife-99914-fig1-data2.zip › Figure 1-source data 2/Aconitase-2 rep2.tif]

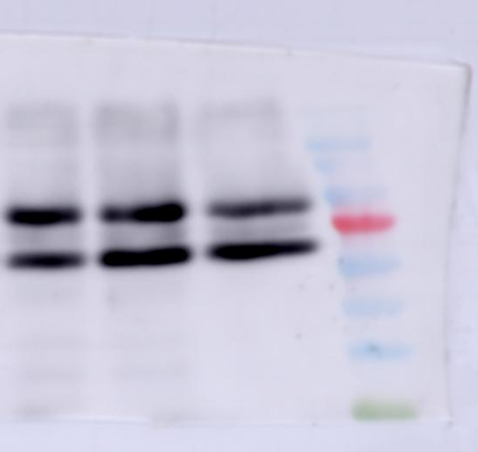

Supplement: Figure 1—source data 2. [file elife-99914-fig1-data2.zip › Figure 1-source data 2/Aconitase-2 rep3.tif]

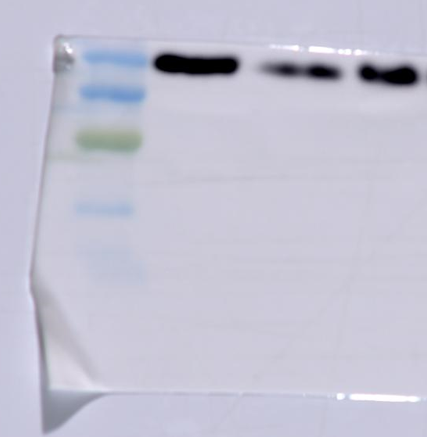

Supplement: Figure 1—source data 2. [file elife-99914-fig1-data2.zip › Figure 1-source data 2/mtHsp60 Actin rep1.tif]

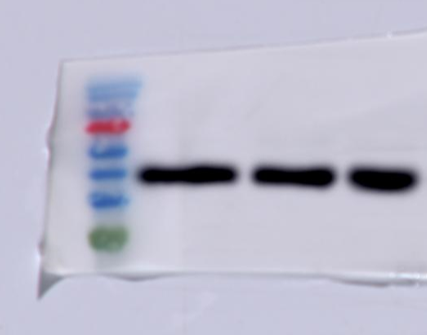

Supplement: Figure 1—source data 2. [file elife-99914-fig1-data2.zip › Figure 1-source data 2/mtHsp60 Actin rep2.tif]

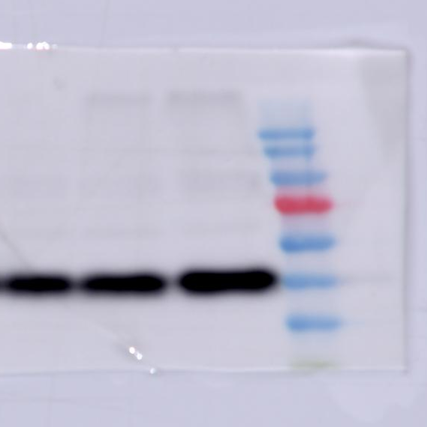

Supplement: Figure 1—source data 2. [file elife-99914-fig1-data2.zip › Figure 1-source data 2/mtHsp60 Actin rep3.tif]

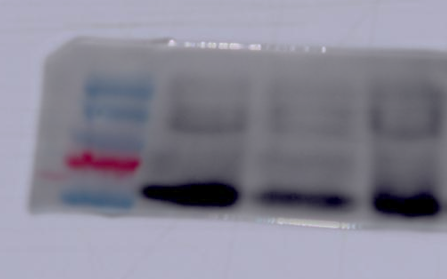

Supplement: Figure 1—source data 2. [file elife-99914-fig1-data2.zip › Figure 1-source data 2/mtHsp60 rep1.tif]

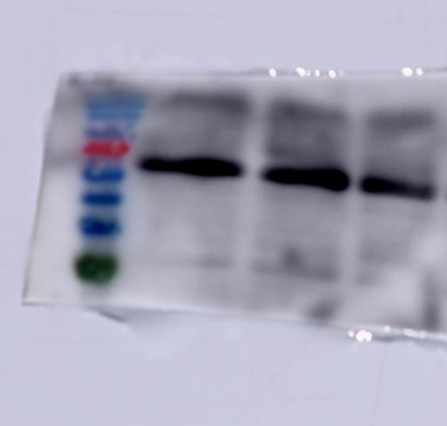

Supplement: Figure 1—source data 2. [file elife-99914-fig1-data2.zip › Figure 1-source data 2/mtHsp60 rep2.tif]

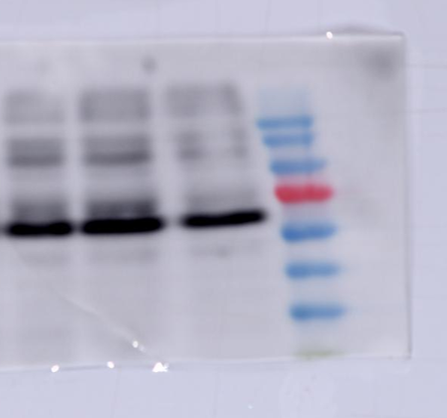

Supplement: Figure 1—source data 2. [file elife-99914-fig1-data2.zip › Figure 1-source data 2/mtHsp60 rep3.tif]

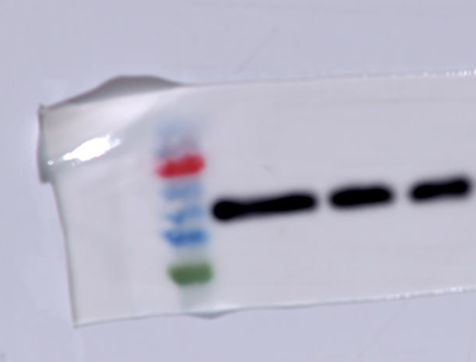

Supplement: Figure 1—source data 2. [file elife-99914-fig1-data2.zip › Figure 1-source data 2/Pam16 Actin rep1.tif]

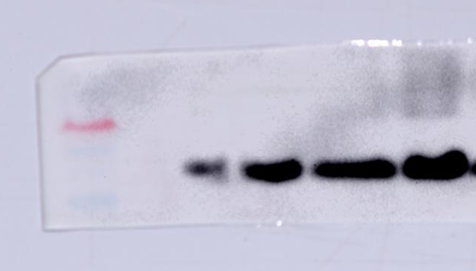

Supplement: Figure 1—source data 2. [file elife-99914-fig1-data2.zip › Figure 1-source data 2/Pam16 Actin rep2.tif]

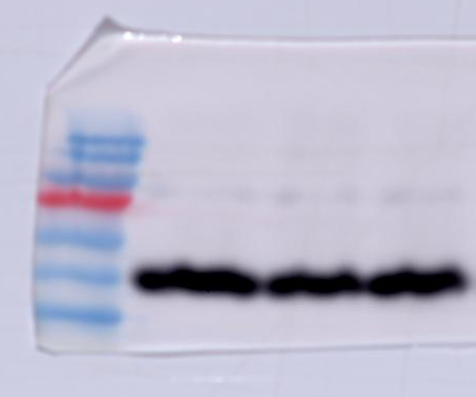

Supplement: Figure 1—source data 2. [file elife-99914-fig1-data2.zip › Figure 1-source data 2/Pam16 Actin rep3.tif]

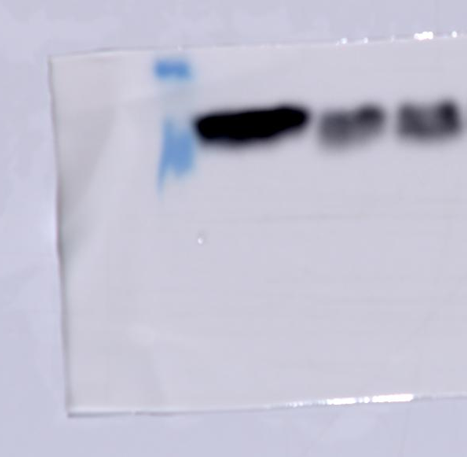

Supplement: Figure 1—source data 2. [file elife-99914-fig1-data2.zip › Figure 1-source data 2/Pam16 rep1.tif]

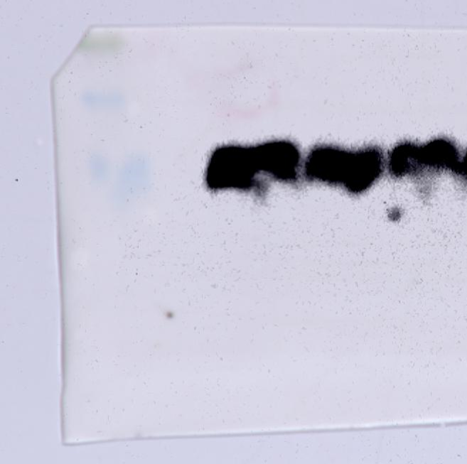

Supplement: Figure 1—source data 2. [file elife-99914-fig1-data2.zip › Figure 1-source data 2/Pam16 rep2.tif]

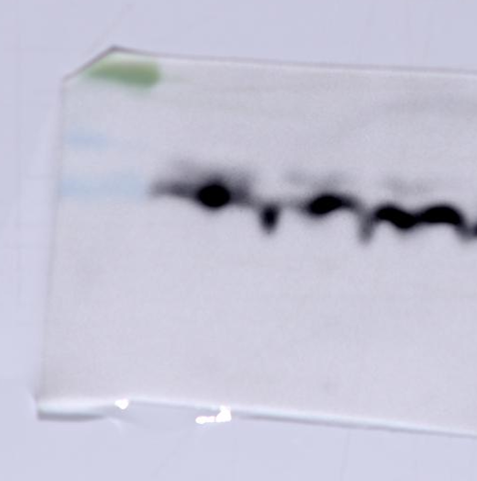

Supplement: Figure 1—source data 2. [file elife-99914-fig1-data2.zip › Figure 1-source data 2/Pam16 rep3.tif]

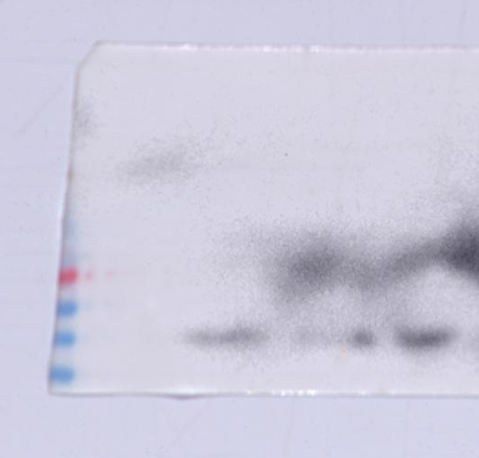

Supplement: Figure 1—source data 2. [file elife-99914-fig1-data2.zip › Figure 1-source data 2/TIMM17A Actin rep1.tif]

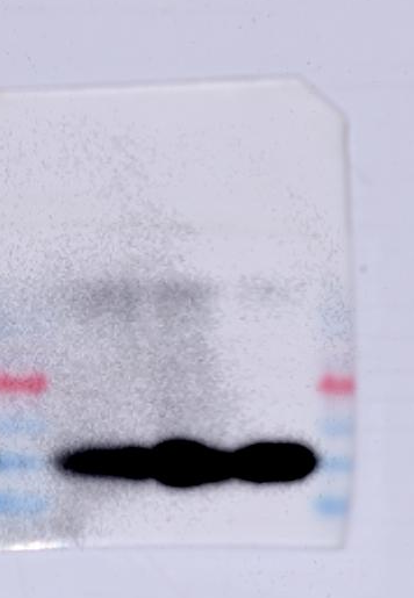

Supplement: Figure 1—source data 2. [file elife-99914-fig1-data2.zip › Figure 1-source data 2/TIMM17A Actin rep2.tif]

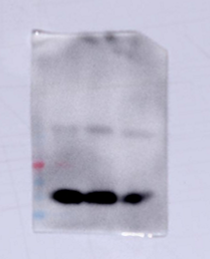

Supplement: Figure 1—source data 2. [file elife-99914-fig1-data2.zip › Figure 1-source data 2/TIMM17A Actin rep3.tif]

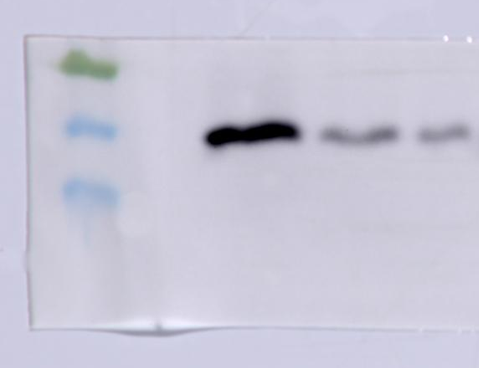

Supplement: Figure 1—source data 2. [file elife-99914-fig1-data2.zip › Figure 1-source data 2/TIMM17A rep1.tif]

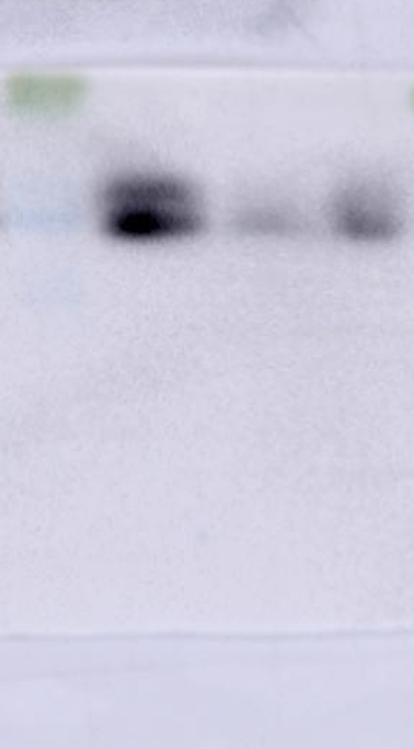

Supplement: Figure 1—source data 2. [file elife-99914-fig1-data2.zip › Figure 1-source data 2/TIMM17A rep2.tif]

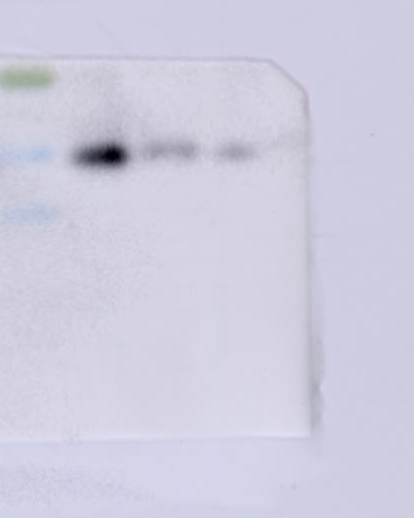

Supplement: Figure 1—source data 2. [file elife-99914-fig1-data2.zip › Figure 1-source data 2/TIMM17A rep3.tif]

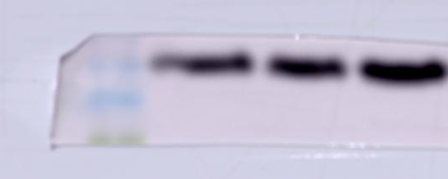

Supplement: Figure 1—source data 2. [file elife-99914-fig1-data2.zip › Figure 1-source data 2/TIMM17B Actin rep1.tif]

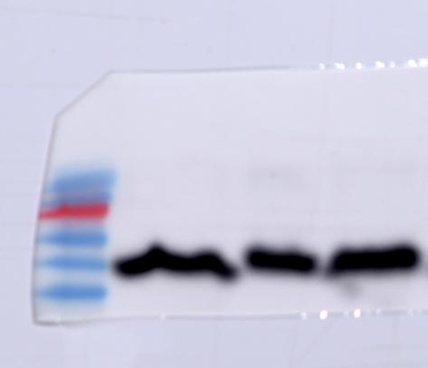

Supplement: Figure 1—source data 2. [file elife-99914-fig1-data2.zip › Figure 1-source data 2/TIMM17B Actin rep3.tif]

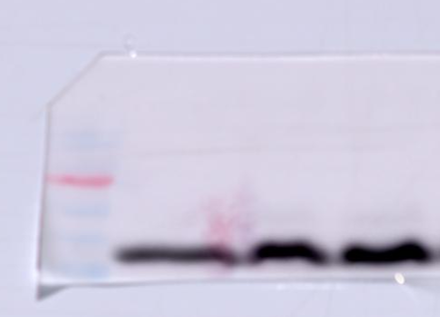

Supplement: Figure 1—source data 2. [file elife-99914-fig1-data2.zip › Figure 1-source data 2/TIMM17B GAPDH rep2.tif]

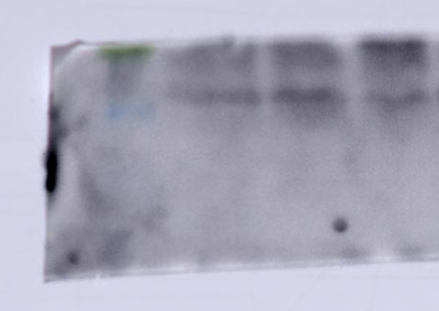

Supplement: Figure 1—source data 2. [file elife-99914-fig1-data2.zip › Figure 1-source data 2/TIMM17B rep1.tif]

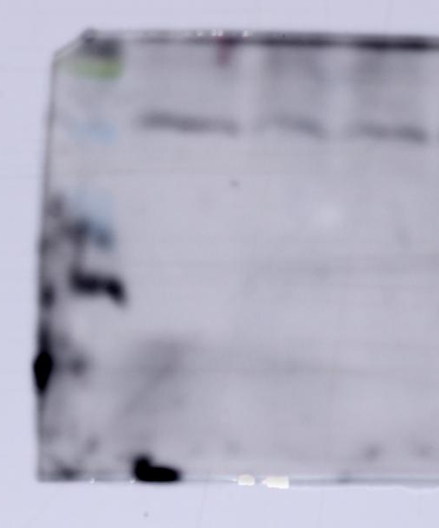

Supplement: Figure 1—source data 2. [file elife-99914-fig1-data2.zip › Figure 1-source data 2/TIMM17B rep2.tif]

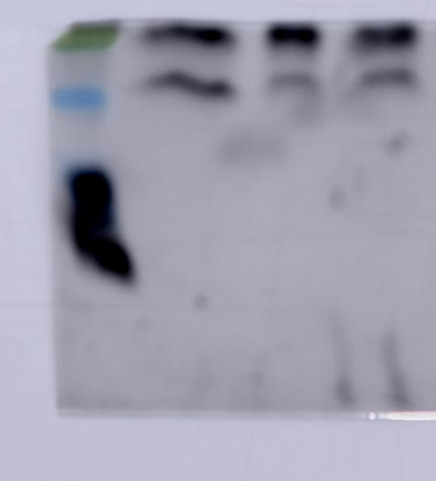

Supplement: Figure 1—source data 2. [file elife-99914-fig1-data2.zip › Figure 1-source data 2/TIMM17B rep3.tif]

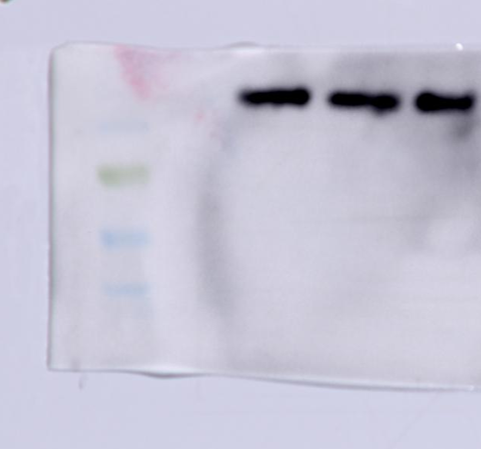

Supplement: Figure 1—source data 2. [file elife-99914-fig1-data2.zip › Figure 1-source data 2/TIMM21 Actin rep1.tif]

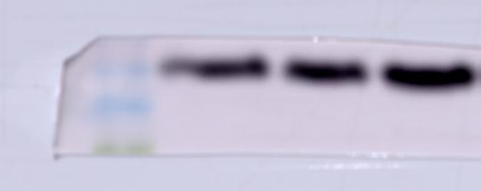

Supplement: Figure 1—source data 2. [file elife-99914-fig1-data2.zip › Figure 1-source data 2/TIMM21 Actin rep2.tif]

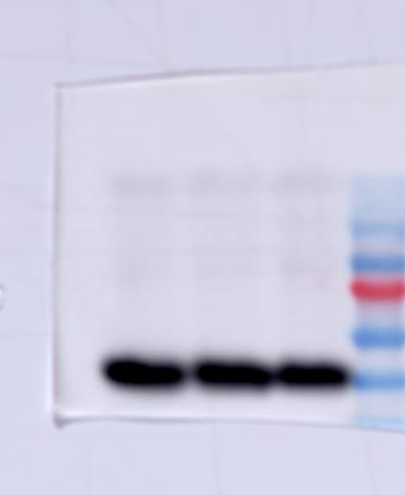

Supplement: Figure 1—source data 2. [file elife-99914-fig1-data2.zip › Figure 1-source data 2/TIMM21 Actin rep3.tif]

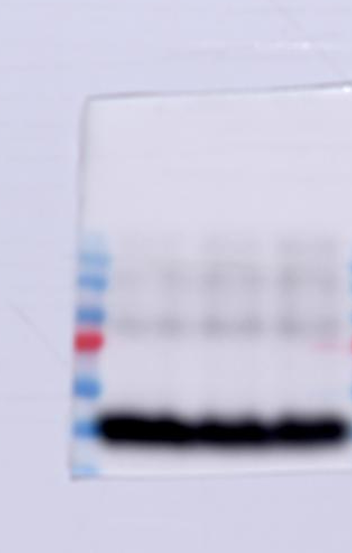

Supplement: Figure 1—source data 2. [file elife-99914-fig1-data2.zip › Figure 1-source data 2/TIMM21 Actin rep4.tif]

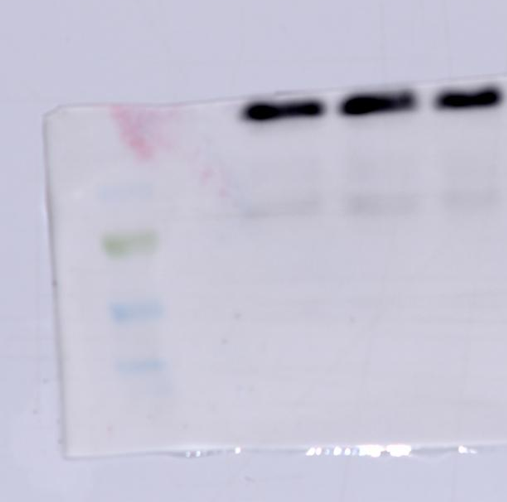

Supplement: Figure 1—source data 2. [file elife-99914-fig1-data2.zip › Figure 1-source data 2/TIMM21 rep1.tif]

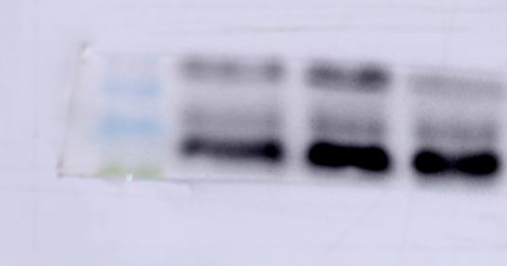

Supplement: Figure 1—source data 2. [file elife-99914-fig1-data2.zip › Figure 1-source data 2/TIMM21 rep2.tif]

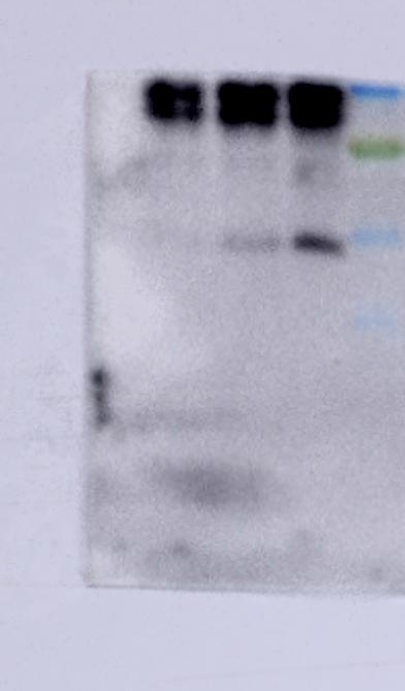

Supplement: Figure 1—source data 2. [file elife-99914-fig1-data2.zip › Figure 1-source data 2/TIMM21 rep3.tif]

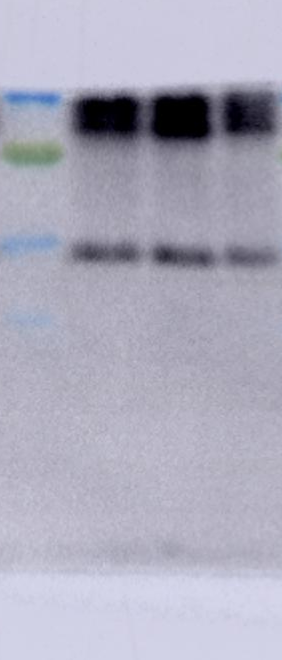

Supplement: Figure 1—source data 2. [file elife-99914-fig1-data2.zip › Figure 1-source data 2/TIMM21 rep4.tif]

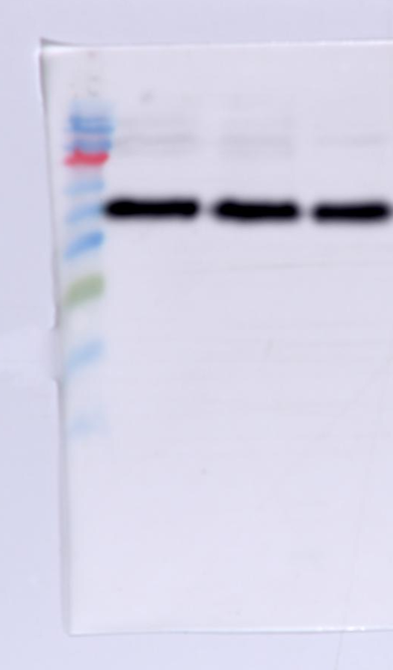

Supplement: Figure 1—source data 2. [file elife-99914-fig1-data2.zip › Figure 1-source data 2/TIMM23 Actin rep1.tif]

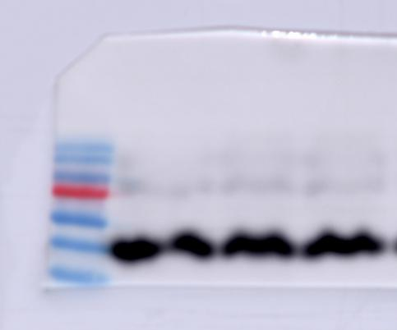

Supplement: Figure 1—source data 2. [file elife-99914-fig1-data2.zip › Figure 1-source data 2/TIMM23 Actin rep3.tif]

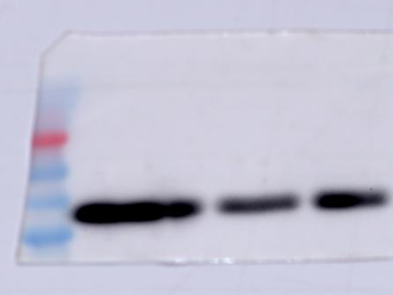

Supplement: Figure 1—source data 2. [file elife-99914-fig1-data2.zip › Figure 1-source data 2/TIMM23 GAPDH rep2.tif]

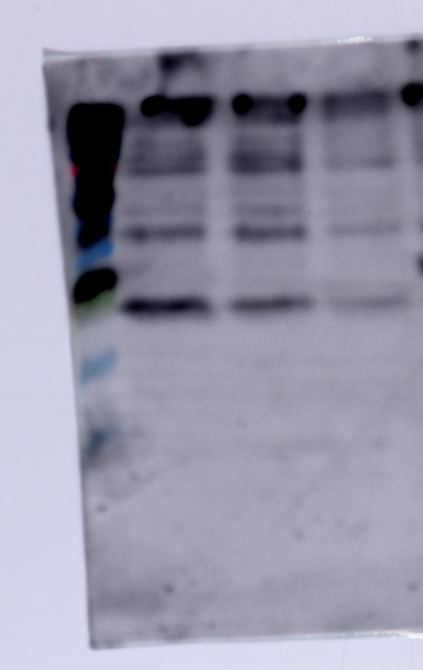

Supplement: Figure 1—source data 2. [file elife-99914-fig1-data2.zip › Figure 1-source data 2/TIMM23 rep1.tif]

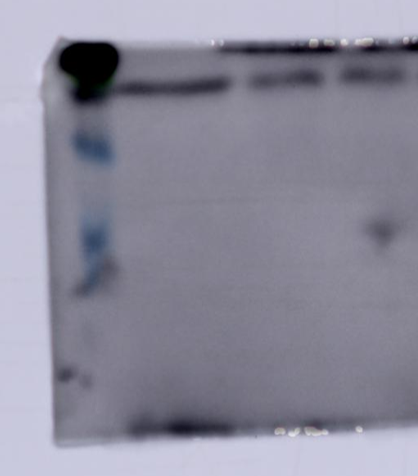

Supplement: Figure 1—source data 2. [file elife-99914-fig1-data2.zip › Figure 1-source data 2/TIMM23 rep2.tif]

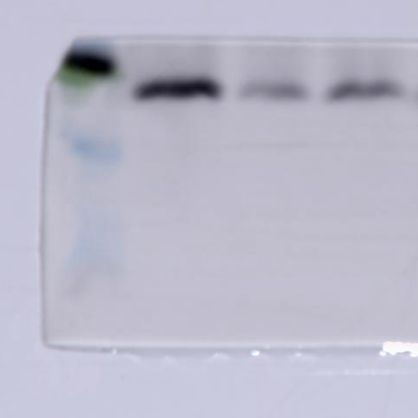

Supplement: Figure 1—source data 2. [file elife-99914-fig1-data2.zip › Figure 1-source data 2/TIMM23 rep3.tif]

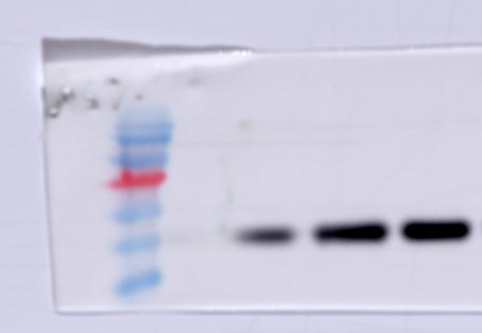

Supplement: Figure 1—source data 2. [file elife-99914-fig1-data2.zip › Figure 1-source data 2/TIMM44 Actin rep1.tif]

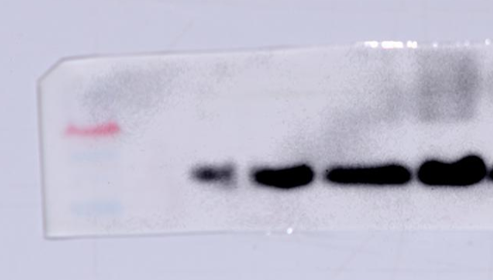

Supplement: Figure 1—source data 2. [file elife-99914-fig1-data2.zip › Figure 1-source data 2/TIMM44 Actin rep2.tif]

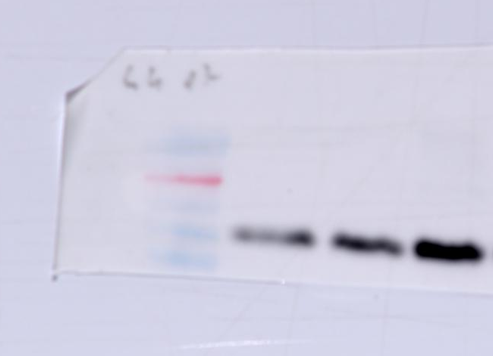

Supplement: Figure 1—source data 2. [file elife-99914-fig1-data2.zip › Figure 1-source data 2/TIMM44 Actin rep3.tif]

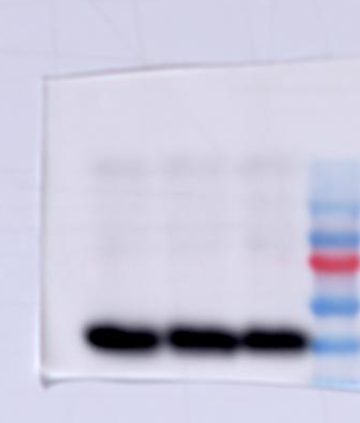

Supplement: Figure 1—source data 2. [file elife-99914-fig1-data2.zip › Figure 1-source data 2/TIMM44 Actin rep4.tif]

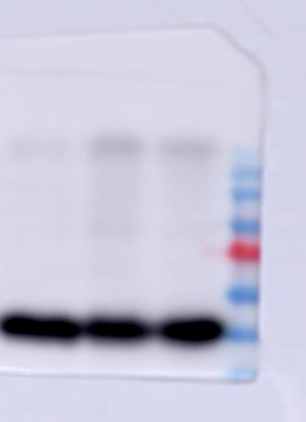

Supplement: Figure 1—source data 2. [file elife-99914-fig1-data2.zip › Figure 1-source data 2/TIMM44 Actin rep5.tif]

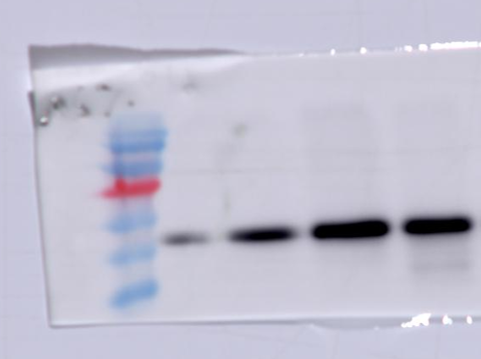

Supplement: Figure 1—source data 2. [file elife-99914-fig1-data2.zip › Figure 1-source data 2/TIMM44 rep1.tif]

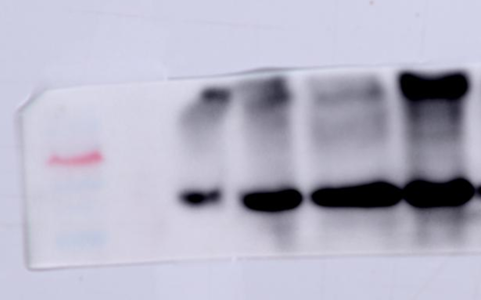

Supplement: Figure 1—source data 2. [file elife-99914-fig1-data2.zip › Figure 1-source data 2/TIMM44 rep2.tif]

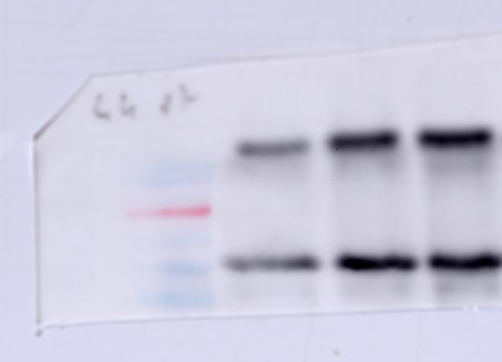

Supplement: Figure 1—source data 2. [file elife-99914-fig1-data2.zip › Figure 1-source data 2/TIMM44 rep3.tif]

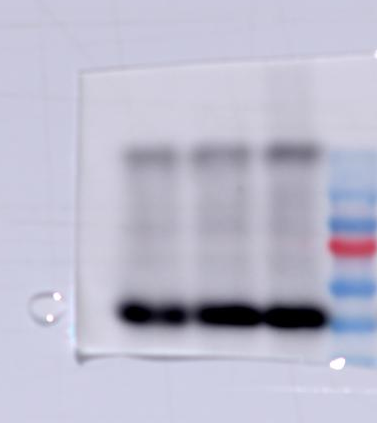

Supplement: Figure 1—source data 2. [file elife-99914-fig1-data2.zip › Figure 1-source data 2/TIMM44 rep4.tif]

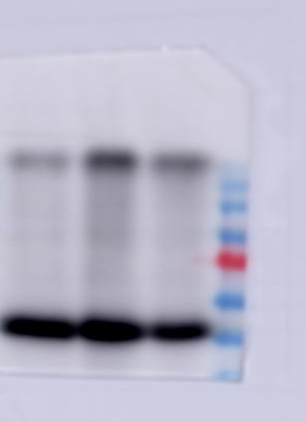

Supplement: Figure 1—source data 2. [file elife-99914-fig1-data2.zip › Figure 1-source data 2/TIMM44 rep5.tif]

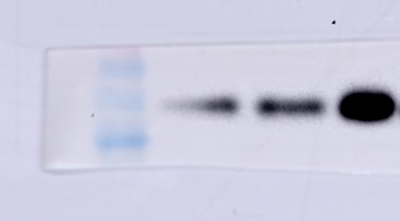

Supplement: Figure 1—source data 2. [file elife-99914-fig1-data2.zip › Figure 1-source data 2/TIMM50 Actin rep1.tif]

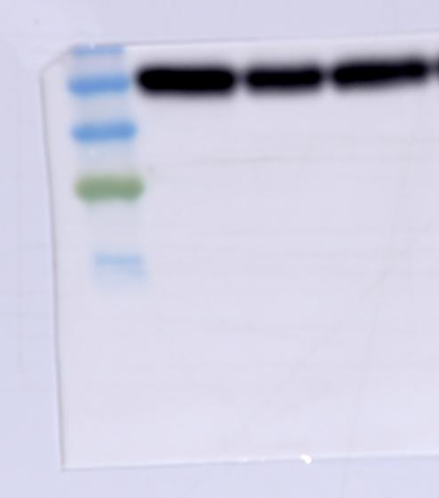

Supplement: Figure 1—source data 2. [file elife-99914-fig1-data2.zip › Figure 1-source data 2/TIMM50 Actin rep2.tif]

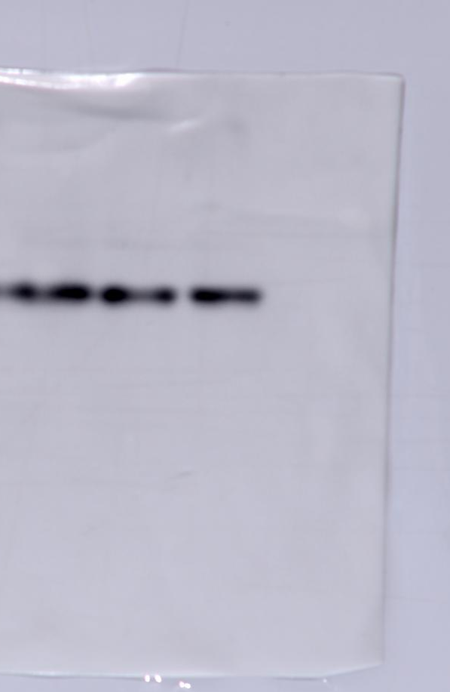

Supplement: Figure 1—source data 2. [file elife-99914-fig1-data2.zip › Figure 1-source data 2/TIMM50 Actin rep3.tif]

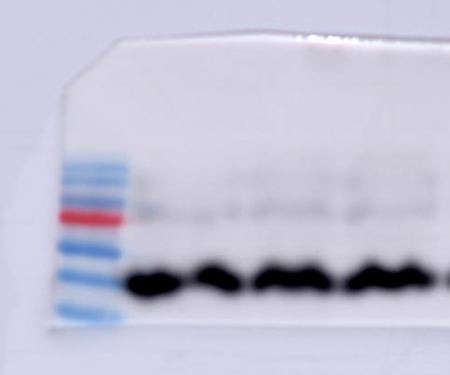

Supplement: Figure 1—source data 2. [file elife-99914-fig1-data2.zip › Figure 1-source data 2/TIMM50 Actin rep4.tif]

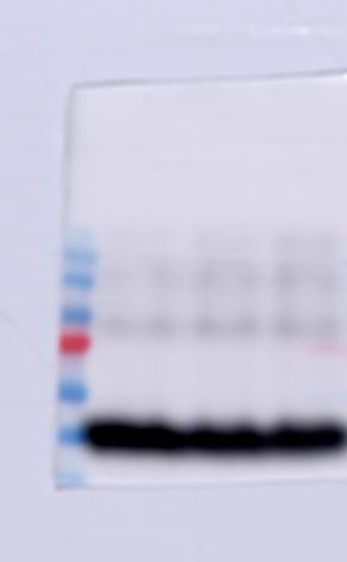

Supplement: Figure 1—source data 2. [file elife-99914-fig1-data2.zip › Figure 1-source data 2/TIMM50 Actin rep5.tif]

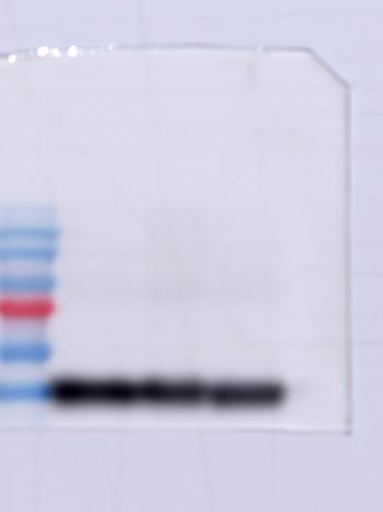

Supplement: Figure 1—source data 2. [file elife-99914-fig1-data2.zip › Figure 1-source data 2/TIMM50 Actin rep6.tif]

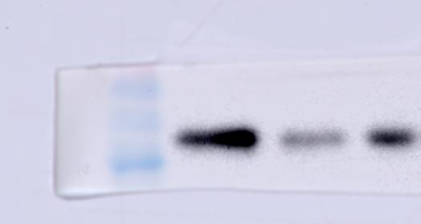

Supplement: Figure 1—source data 2. [file elife-99914-fig1-data2.zip › Figure 1-source data 2/TIMM50 rep1.tif]

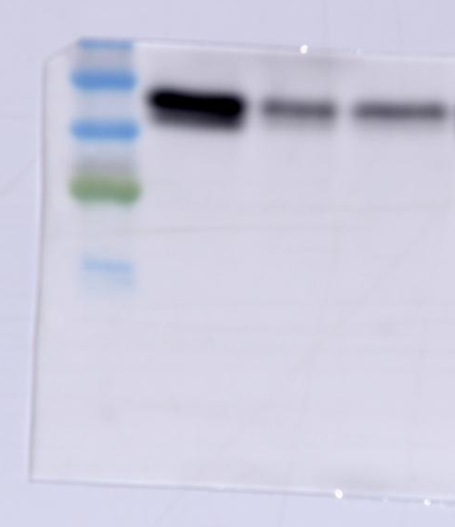

Supplement: Figure 1—source data 2. [file elife-99914-fig1-data2.zip › Figure 1-source data 2/TIMM50 rep2.tif]

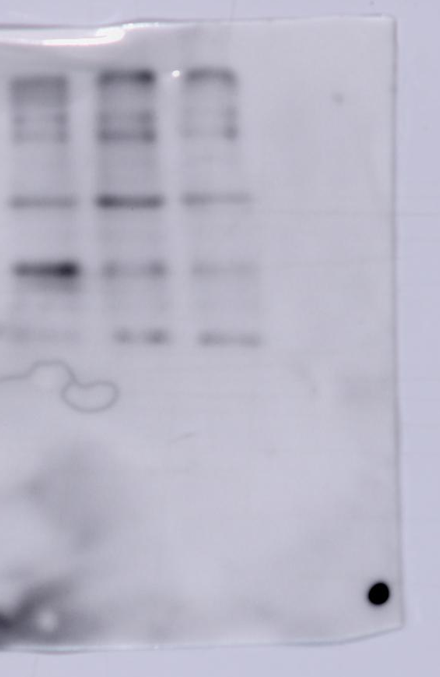

Supplement: Figure 1—source data 2. [file elife-99914-fig1-data2.zip › Figure 1-source data 2/TIMM50 rep3.tif]

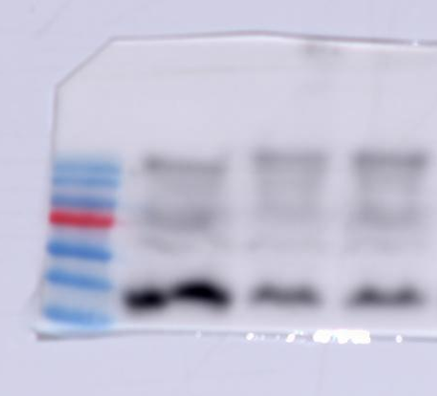

Supplement: Figure 1—source data 2. [file elife-99914-fig1-data2.zip › Figure 1-source data 2/TIMM50 rep4.tif]

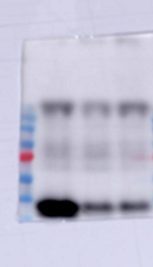

Supplement: Figure 1—source data 2. [file elife-99914-fig1-data2.zip › Figure 1-source data 2/TIMM50 rep5.tif]

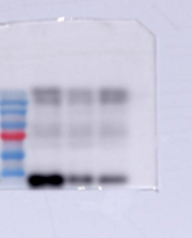

Supplement: Figure 1—source data 2. [file elife-99914-fig1-data2.zip › Figure 1-source data 2/TIMM50 rep6.tif]

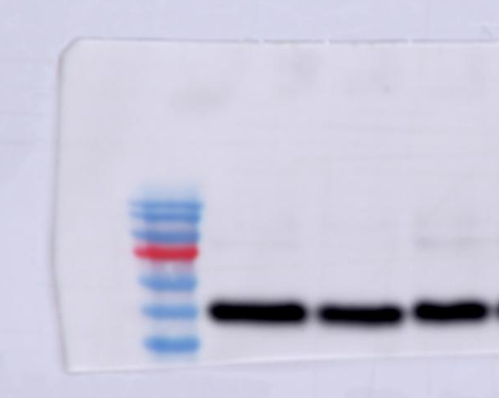

Supplement: Figure 1—source data 2. [file elife-99914-fig1-data2.zip › Figure 1-source data 2/TOMM20 Actin rep1.tif]

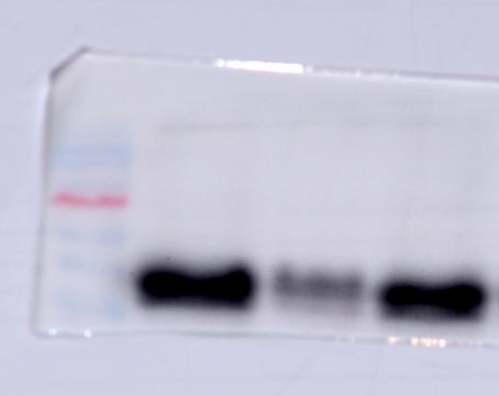

Supplement: Figure 1—source data 2. [file elife-99914-fig1-data2.zip › Figure 1-source data 2/TOMM20 Actin rep2.tif]

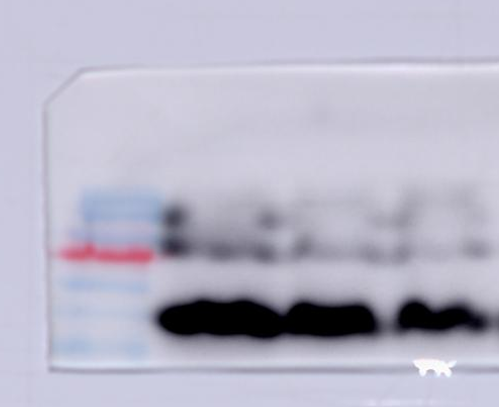

Supplement: Figure 1—source data 2. [file elife-99914-fig1-data2.zip › Figure 1-source data 2/TOMM20 Actin rep3.tif]

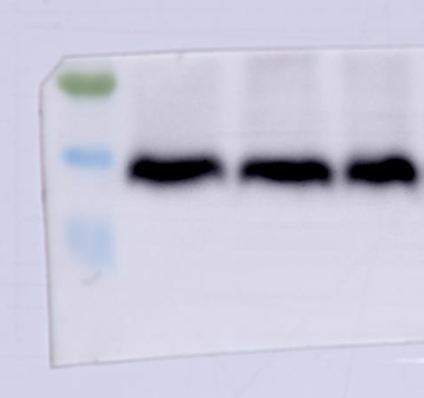

Supplement: Figure 1—source data 2. [file elife-99914-fig1-data2.zip › Figure 1-source data 2/TOMM20 rep1.tif]

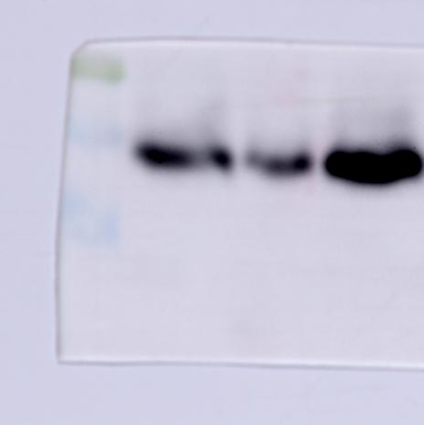

Supplement: Figure 1—source data 2. [file elife-99914-fig1-data2.zip › Figure 1-source data 2/TOMM20 rep2.tif]

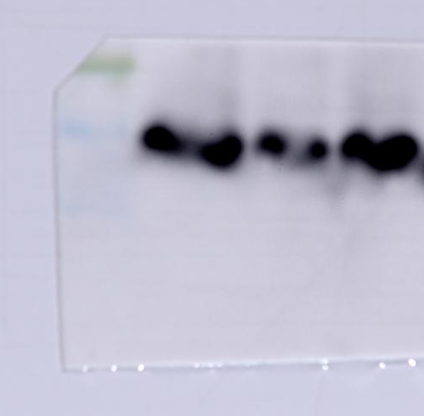

Supplement: Figure 1—source data 2. [file elife-99914-fig1-data2.zip › Figure 1-source data 2/TOMM20 rep3.tif]

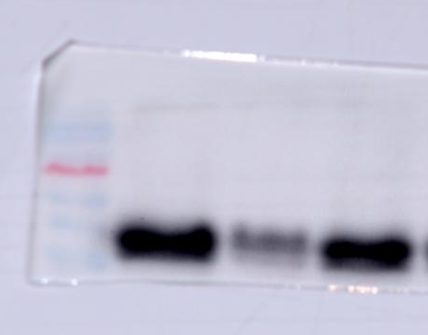

Supplement: Figure 1—source data 2. [file elife-99914-fig1-data2.zip › Figure 1-source data 2/TOMM40 Actin rep1.tif]

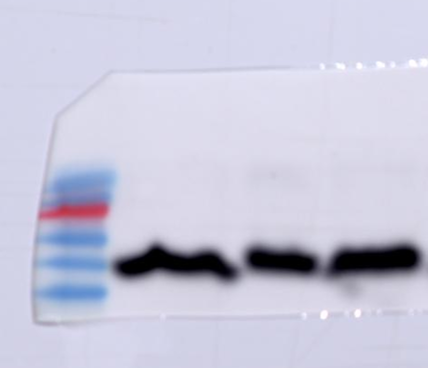

Supplement: Figure 1—source data 2. [file elife-99914-fig1-data2.zip › Figure 1-source data 2/TOMM40 Actin rep2.tif]

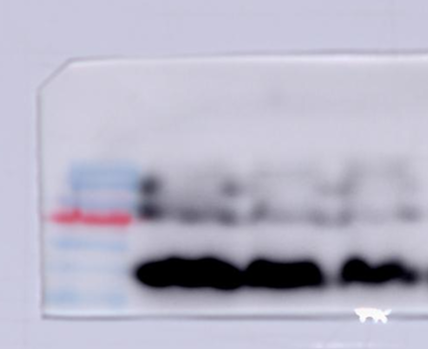

Supplement: Figure 1—source data 2. [file elife-99914-fig1-data2.zip › Figure 1-source data 2/TOMM40 Actin rep3.tif]

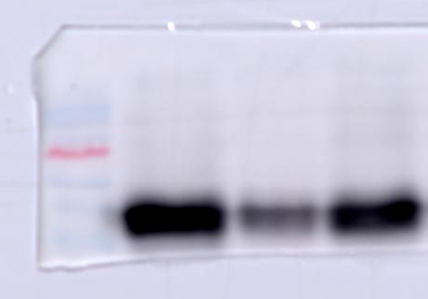

Supplement: Figure 1—source data 2. [file elife-99914-fig1-data2.zip › Figure 1-source data 2/TOMM40 rep1.tif]

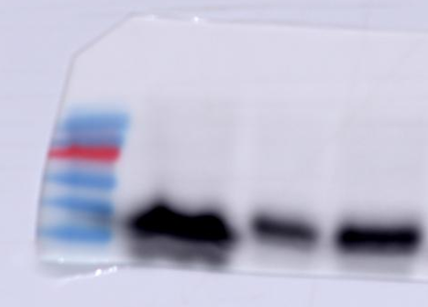

Supplement: Figure 1—source data 2. [file elife-99914-fig1-data2.zip › Figure 1-source data 2/TOMM40 rep2.tif]

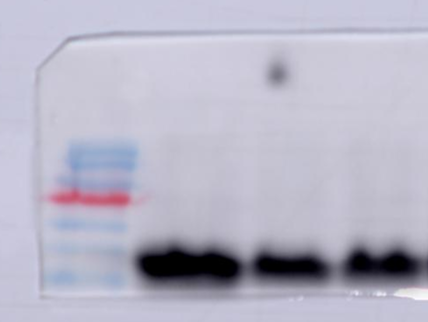

Supplement: Figure 1—source data 2. [file elife-99914-fig1-data2.zip › Figure 1-source data 2/TOMM40 rep3.tif]

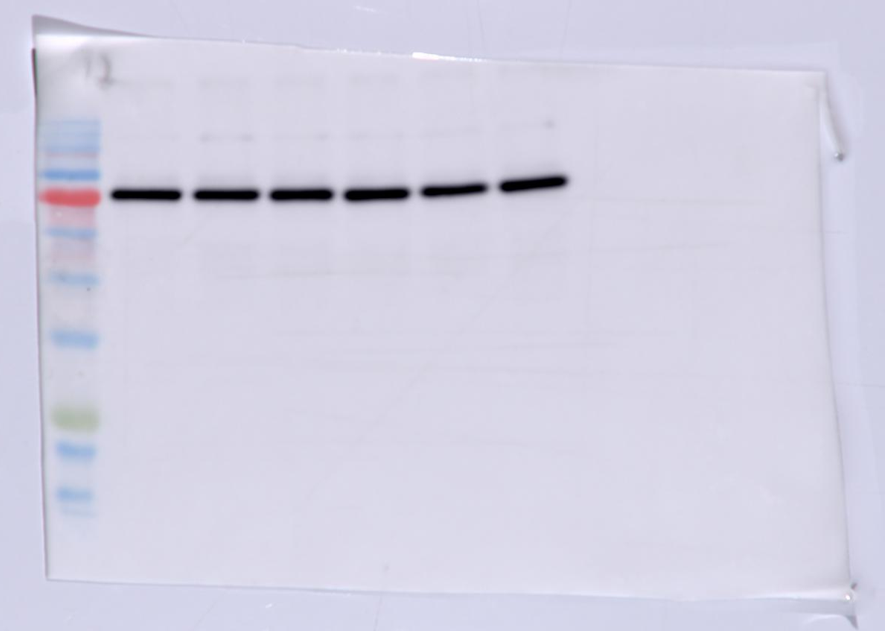

Supplement: Figure 2—source data 2. [file elife-99914-fig2-data2.zip › Figure 2-source data 2/Aconitase-2 rep1.tif]

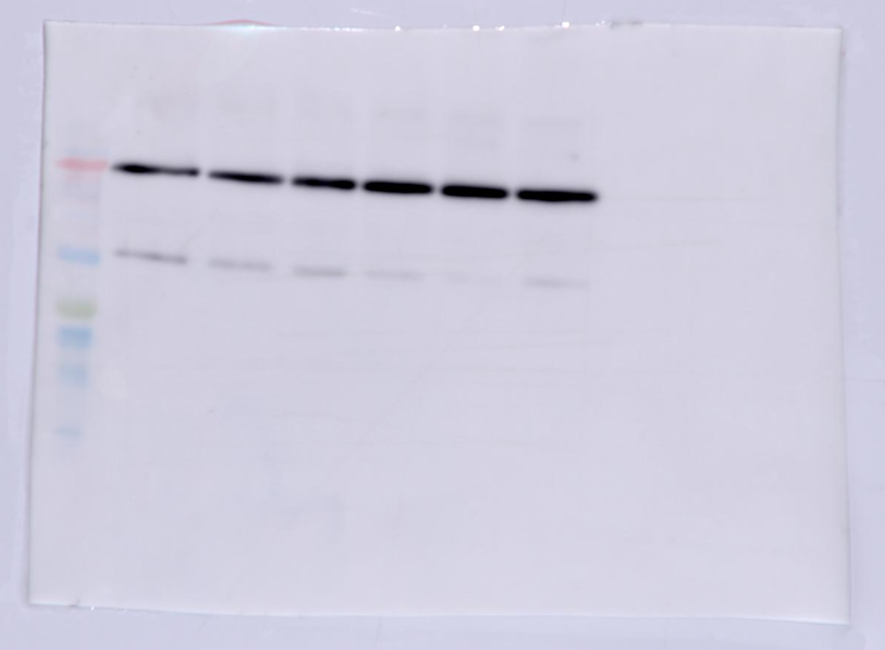

Supplement: Figure 2—source data 2. [file elife-99914-fig2-data2.zip › Figure 2-source data 2/Aconitase-2 rep2.tif]

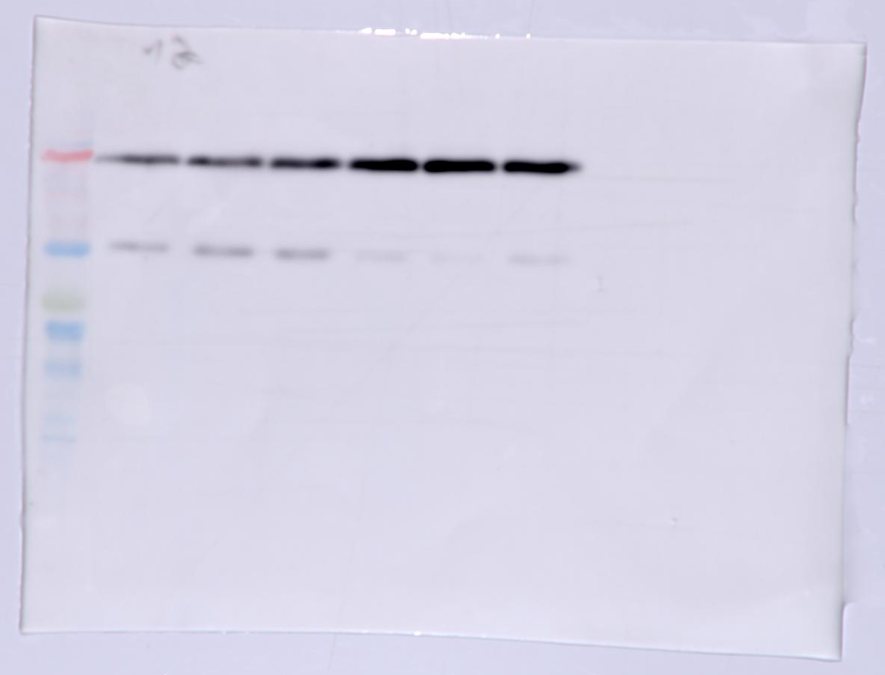

Supplement: Figure 2—source data 2. [file elife-99914-fig2-data2.zip › Figure 2-source data 2/Aconitase-2 rep3.tif]

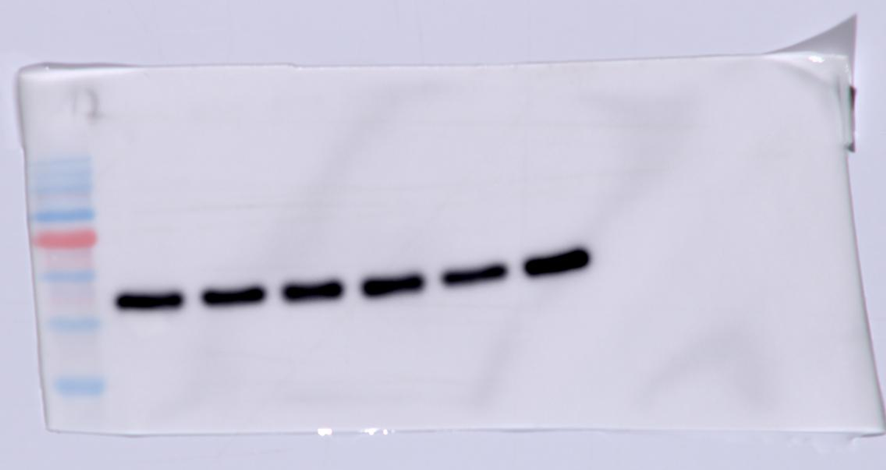

Supplement: Figure 2—source data 2. [file elife-99914-fig2-data2.zip › Figure 2-source data 2/Aconitase-2 Tubulin rep1.tif]

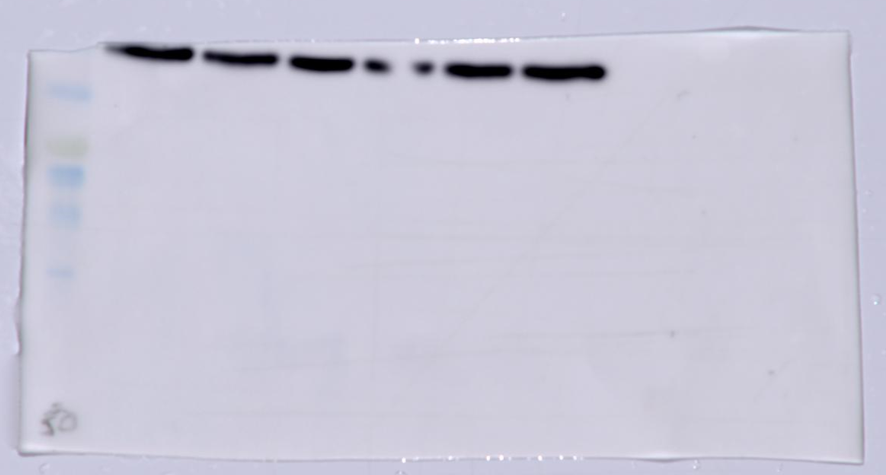

Supplement: Figure 2—source data 2. [file elife-99914-fig2-data2.zip › Figure 2-source data 2/Aconitase-2 Tubulin rep2.tif]

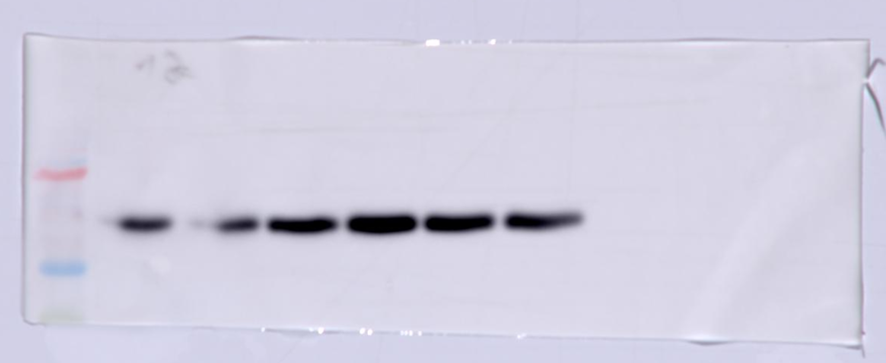

Supplement: Figure 2—source data 2. [file elife-99914-fig2-data2.zip › Figure 2-source data 2/Aconitase-2 Tubulin rep3.tif]

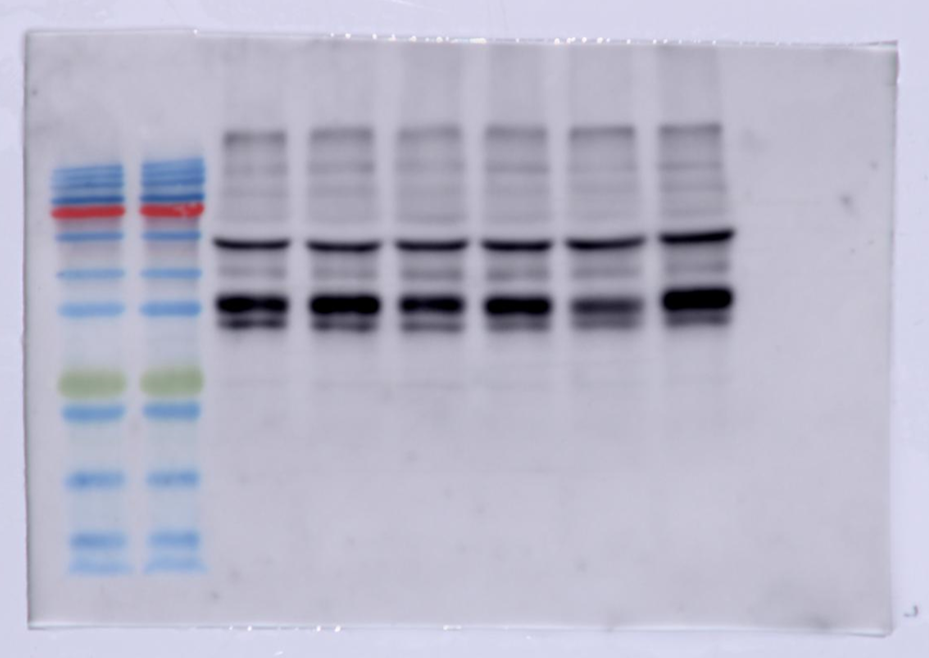

Supplement: Figure 2—source data 2. [file elife-99914-fig2-data2.zip › Figure 2-source data 2/mtHsp60 rep1.tif]

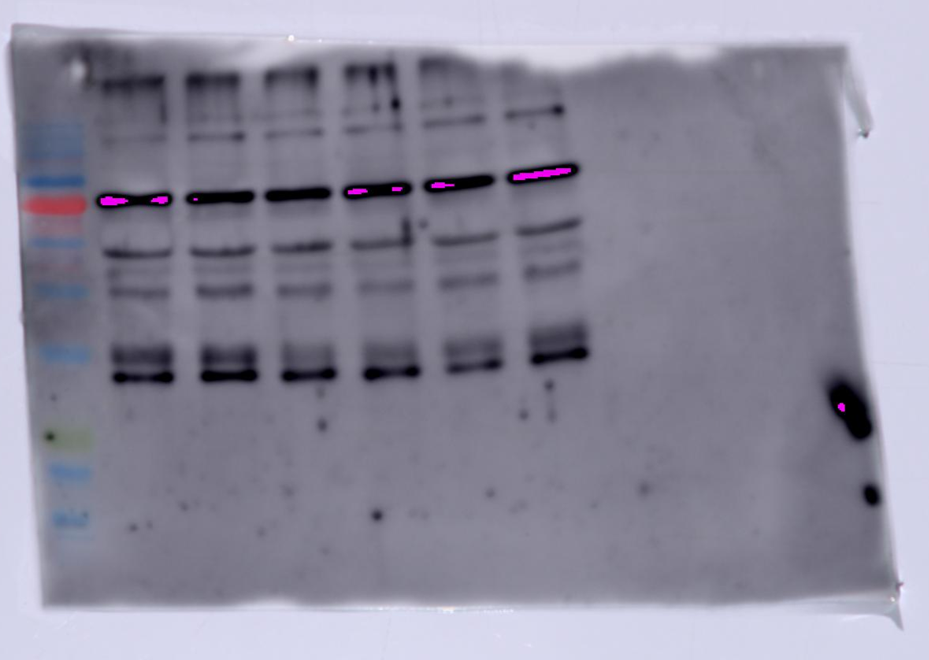

Supplement: Figure 2—source data 2. [file elife-99914-fig2-data2.zip › Figure 2-source data 2/mtHsp60 rep2.tif]

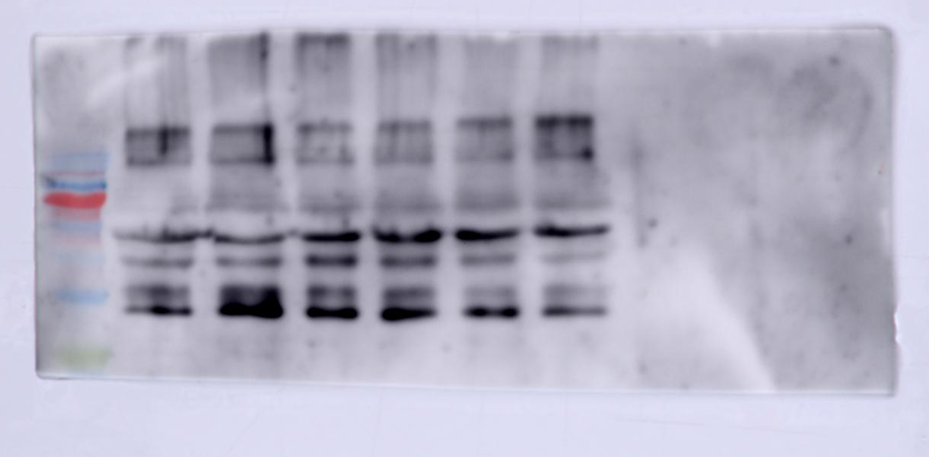

Supplement: Figure 2—source data 2. [file elife-99914-fig2-data2.zip › Figure 2-source data 2/mtHsp60 rep3.tif]

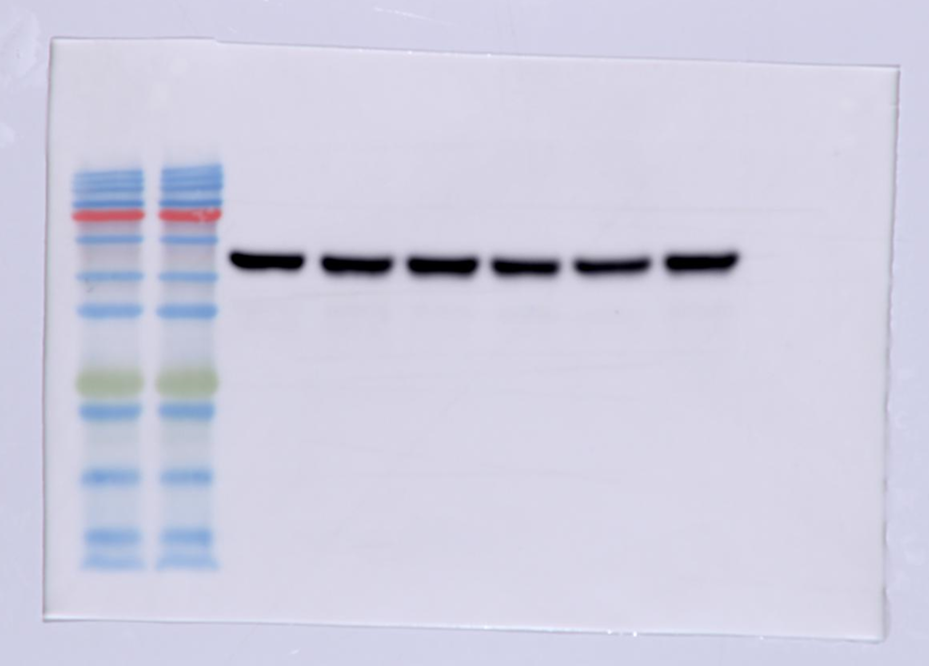

Supplement: Figure 2—source data 2. [file elife-99914-fig2-data2.zip › Figure 2-source data 2/mtHsp60 Tubulin rep1.tif]

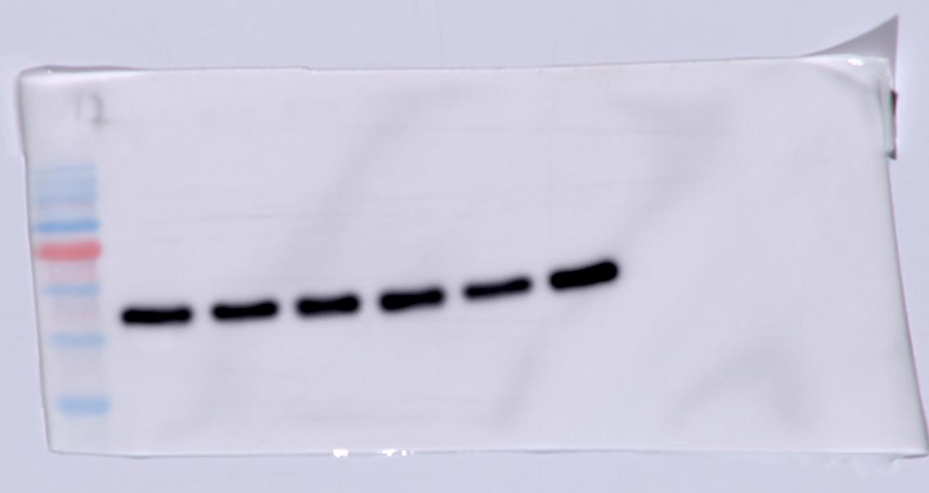

Supplement: Figure 2—source data 2. [file elife-99914-fig2-data2.zip › Figure 2-source data 2/mtHsp60 Tubulin rep2.tif]

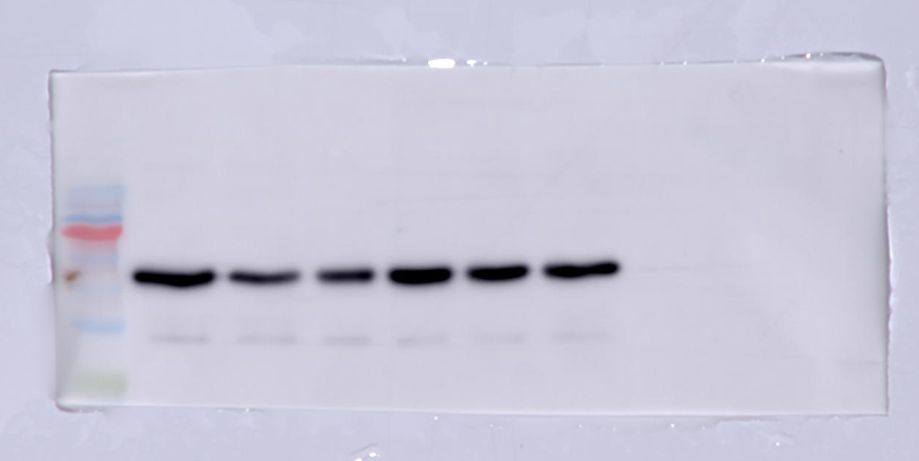

Supplement: Figure 2—source data 2. [file elife-99914-fig2-data2.zip › Figure 2-source data 2/mtHsp60 Tubulin rep3.tif]

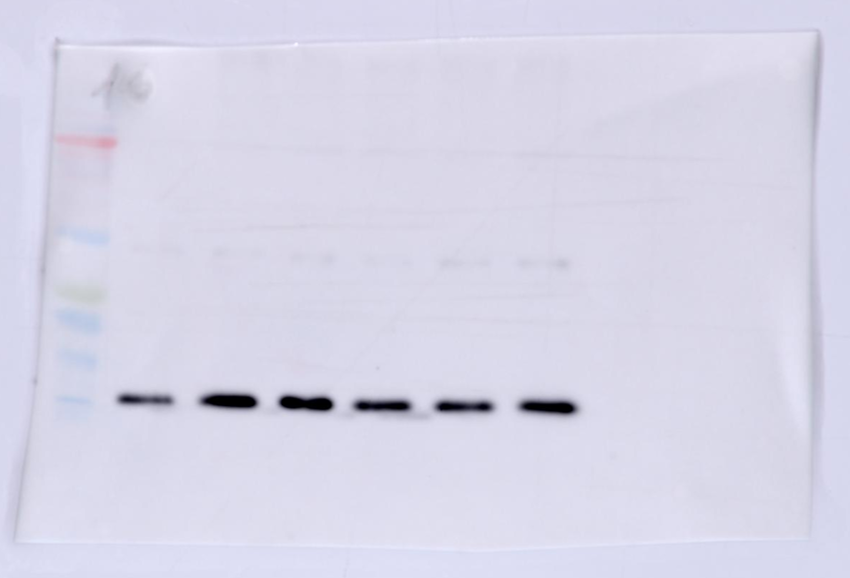

Supplement: Figure 2—source data 2. [file elife-99914-fig2-data2.zip › Figure 2-source data 2/Pam16 rep1.tif]

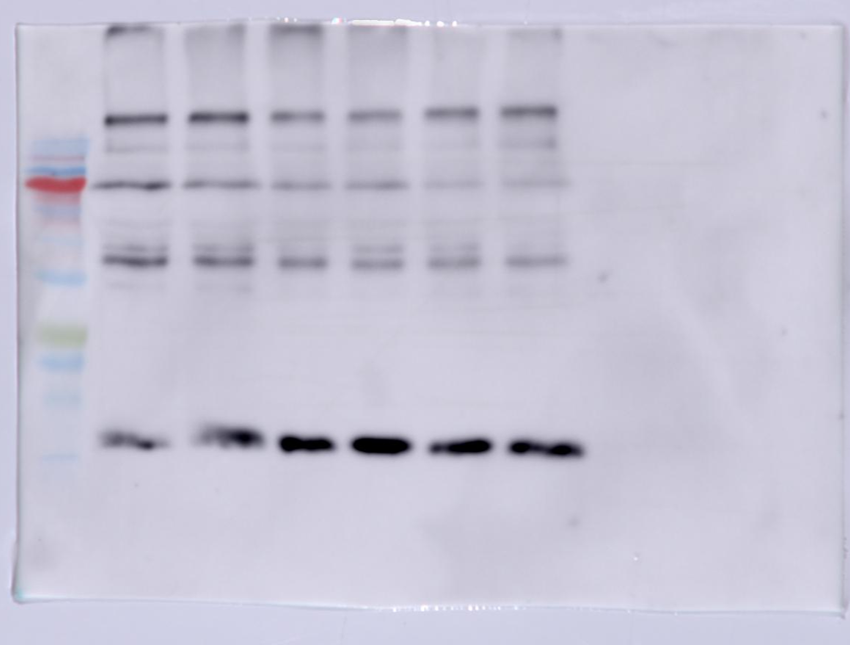

Supplement: Figure 2—source data 2. [file elife-99914-fig2-data2.zip › Figure 2-source data 2/Pam16 rep2.tif]

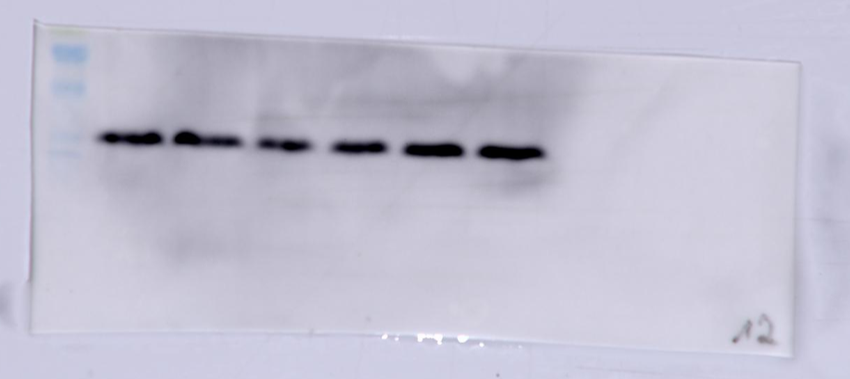

Supplement: Figure 2—source data 2. [file elife-99914-fig2-data2.zip › Figure 2-source data 2/Pam16 rep3.tif]

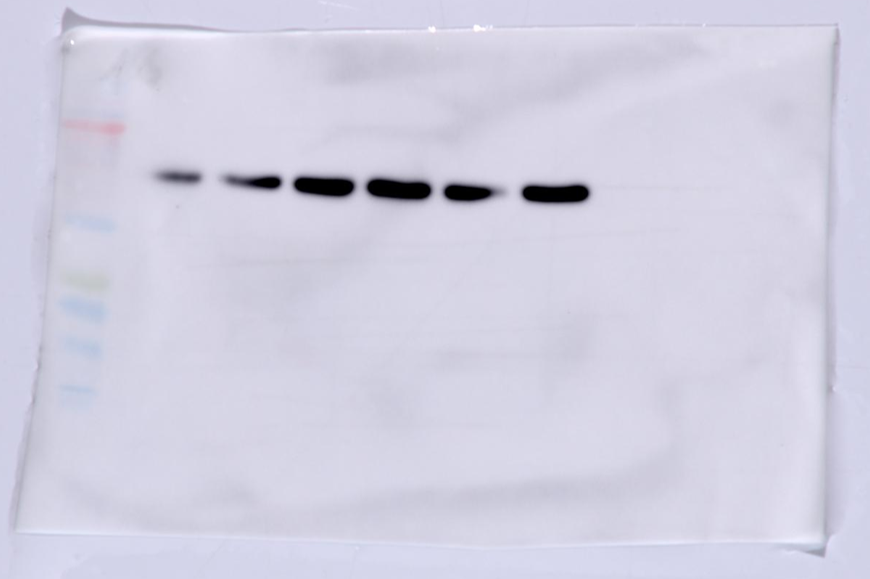

Supplement: Figure 2—source data 2. [file elife-99914-fig2-data2.zip › Figure 2-source data 2/Pam16 Tubulin rep1.tif]

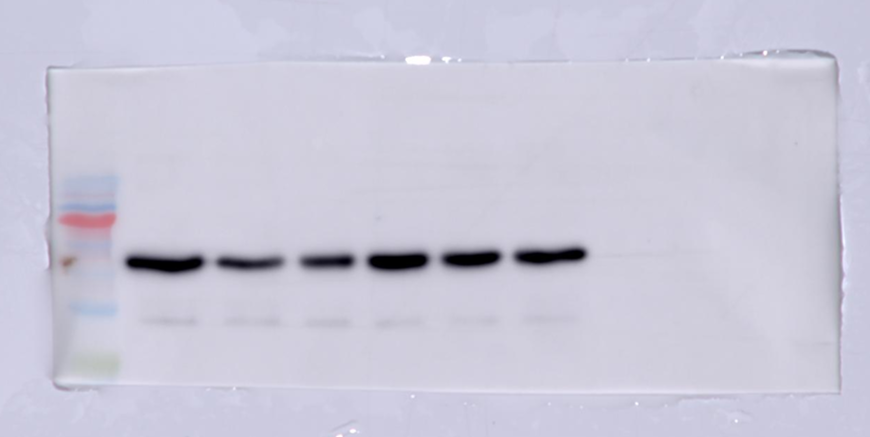

Supplement: Figure 2—source data 2. [file elife-99914-fig2-data2.zip › Figure 2-source data 2/Pam16 Tubulin rep2.tif]

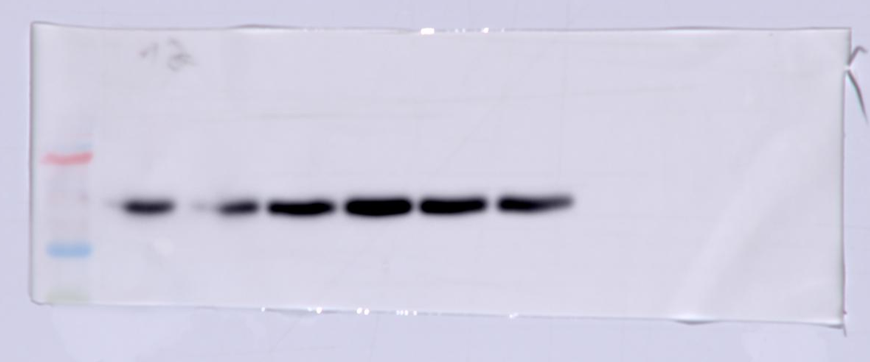

Supplement: Figure 2—source data 2. [file elife-99914-fig2-data2.zip › Figure 2-source data 2/Pam16 Tubulin rep3.tif]

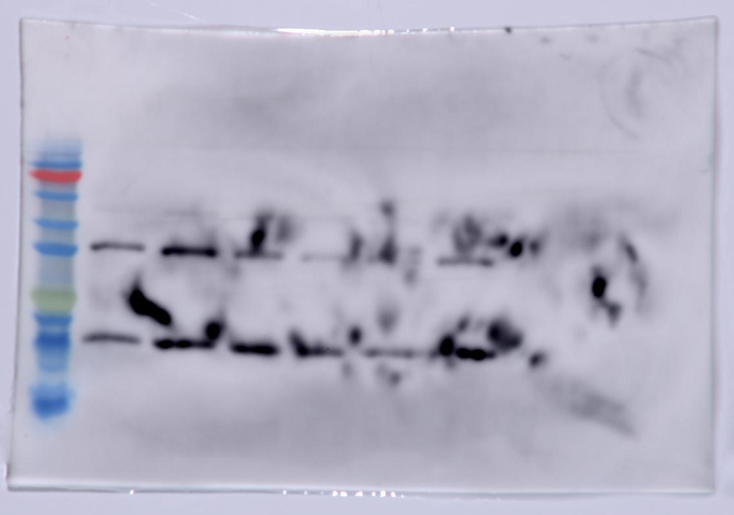

Supplement: Figure 2—source data 2. [file elife-99914-fig2-data2.zip › Figure 2-source data 2/TIMM17A rep1.tif]

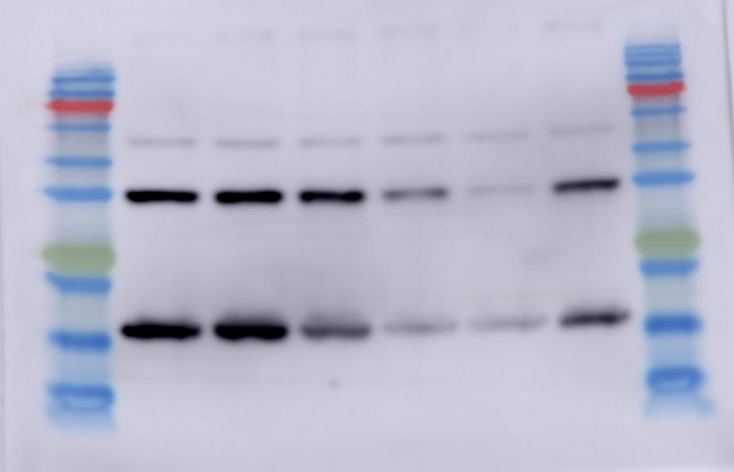

Supplement: Figure 2—source data 2. [file elife-99914-fig2-data2.zip › Figure 2-source data 2/TIMM17A rep2.tif]

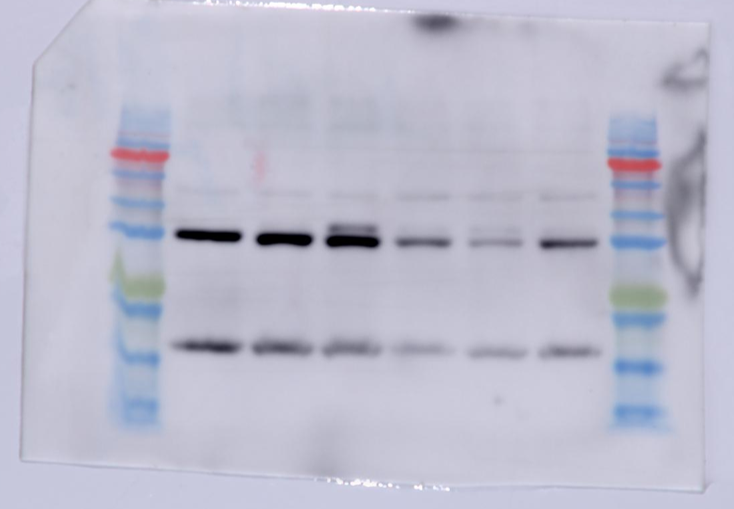

Supplement: Figure 2—source data 2. [file elife-99914-fig2-data2.zip › Figure 2-source data 2/TIMM17A rep3.tif]

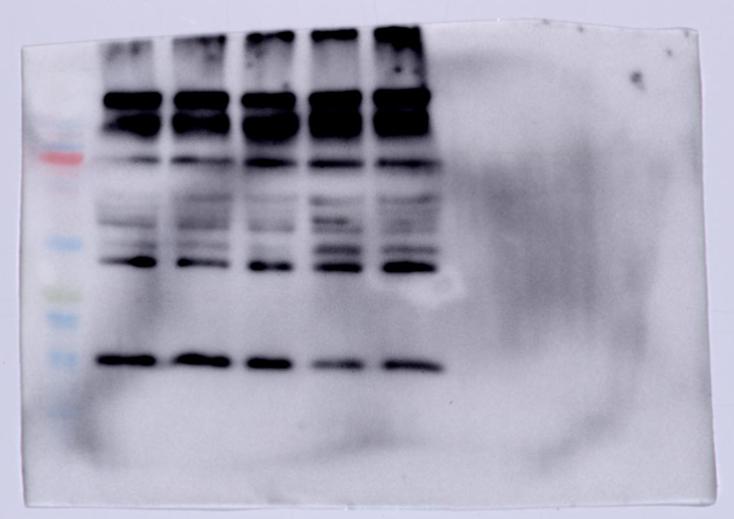

Supplement: Figure 2—source data 2. [file elife-99914-fig2-data2.zip › Figure 2-source data 2/TIMM17A rep4.tif]
