# Supplementary material for: Biochemical and neurophysiological effects of deficiency of the mitochondrial import protein TIMM50
Source: eLife. 2024 Dec 16;13:RP99914. doi: 10.7554/eLife.99914 (PMC11649234; doi:10.7554/eLife.99914)

TIMM50

(Running order is Untreated / pLL3.7 control / Scr control / Sh1 / Sh2 / Sh3)

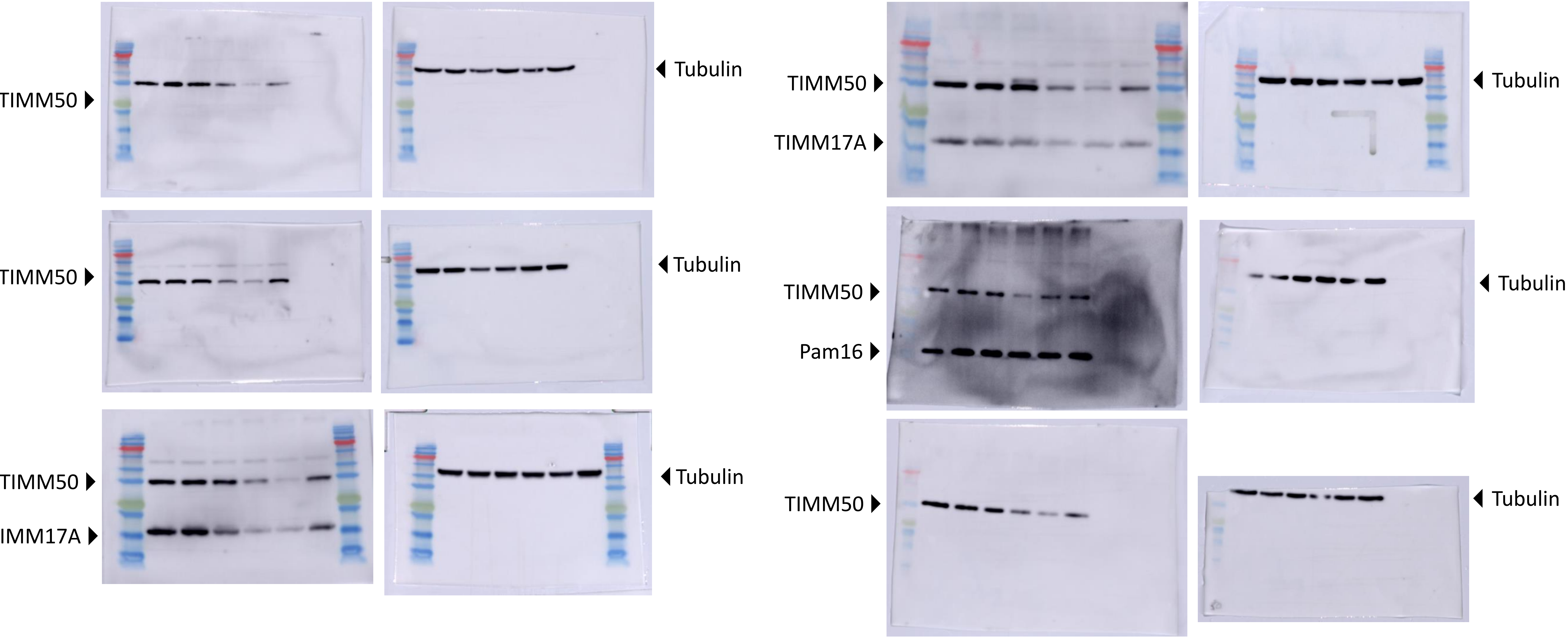

TIMM50

(Running order is Untreated / pLL3.7 control / Scr control / Sh1 / Sh2 / Sh3)

*The bottom blot has only Scr control and Sh2 samples from 4 separate cultures and a different running order than the rest

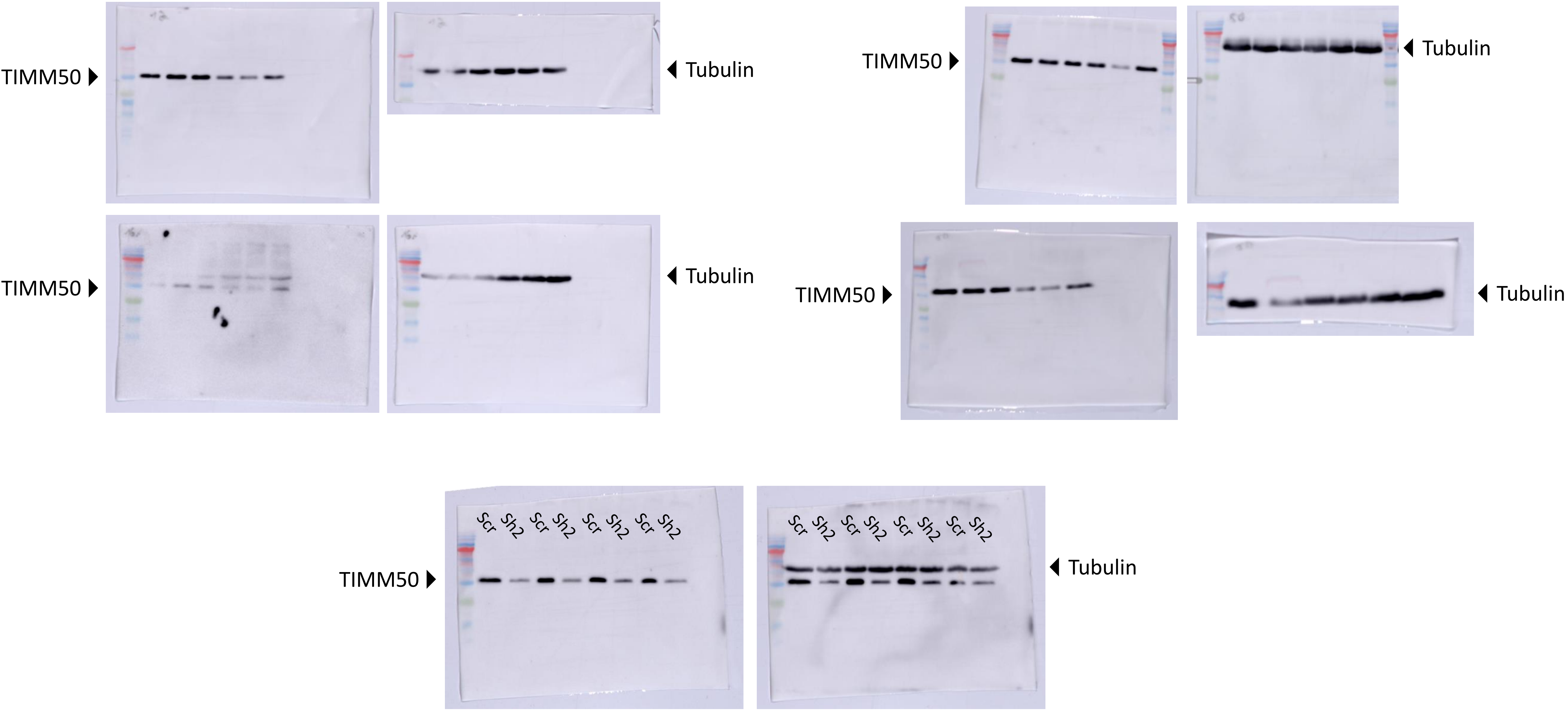

TIMM23

(Running order is Untreated / pLL3.7 control / Scr control / Sh1 / Sh2 / Sh3)

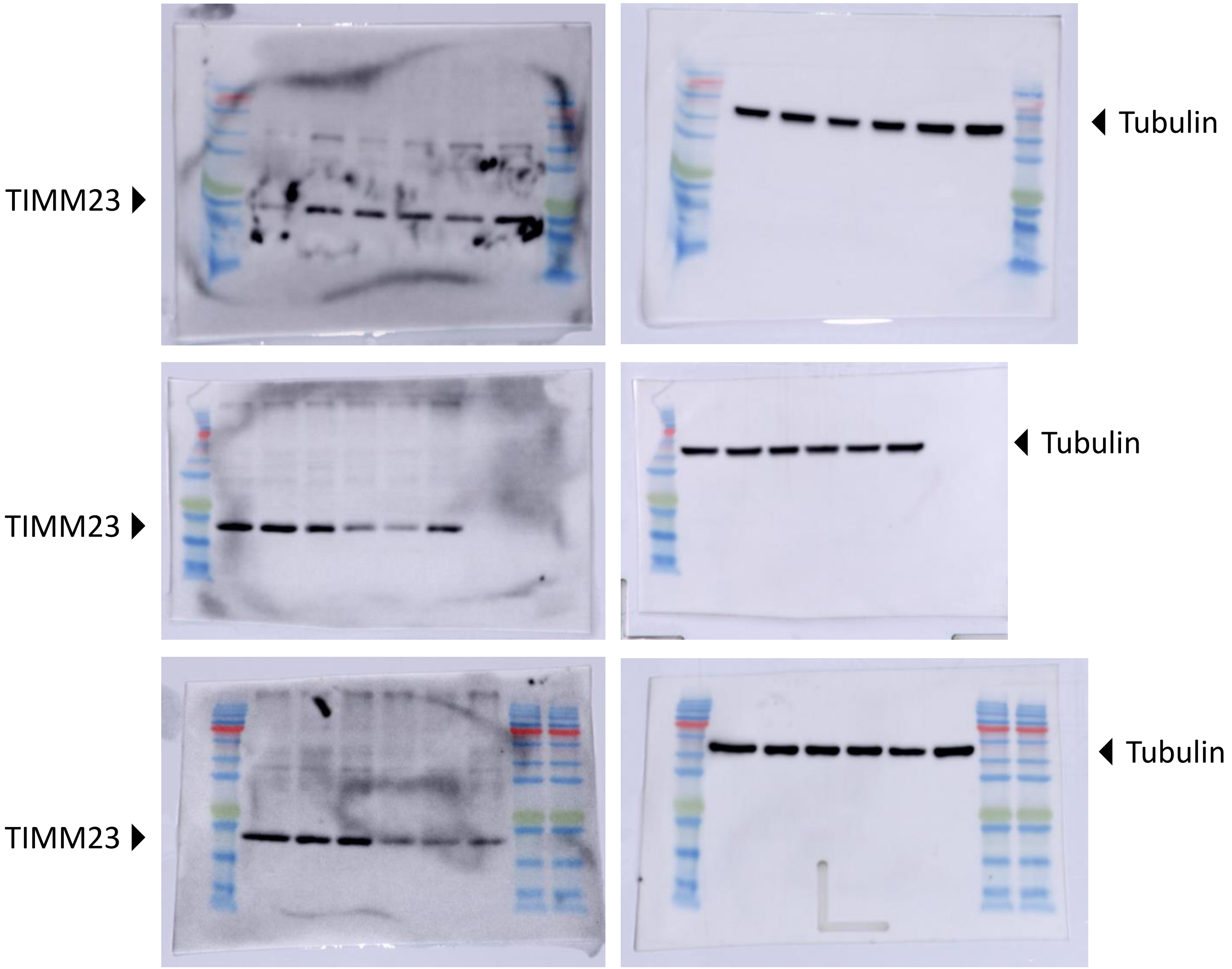

TIMM17A

(Running order is Untreated / pLL3.7 control / Scr control / Sh1 / Sh2 / Sh3)

*Note that the blot on the right had a different running order

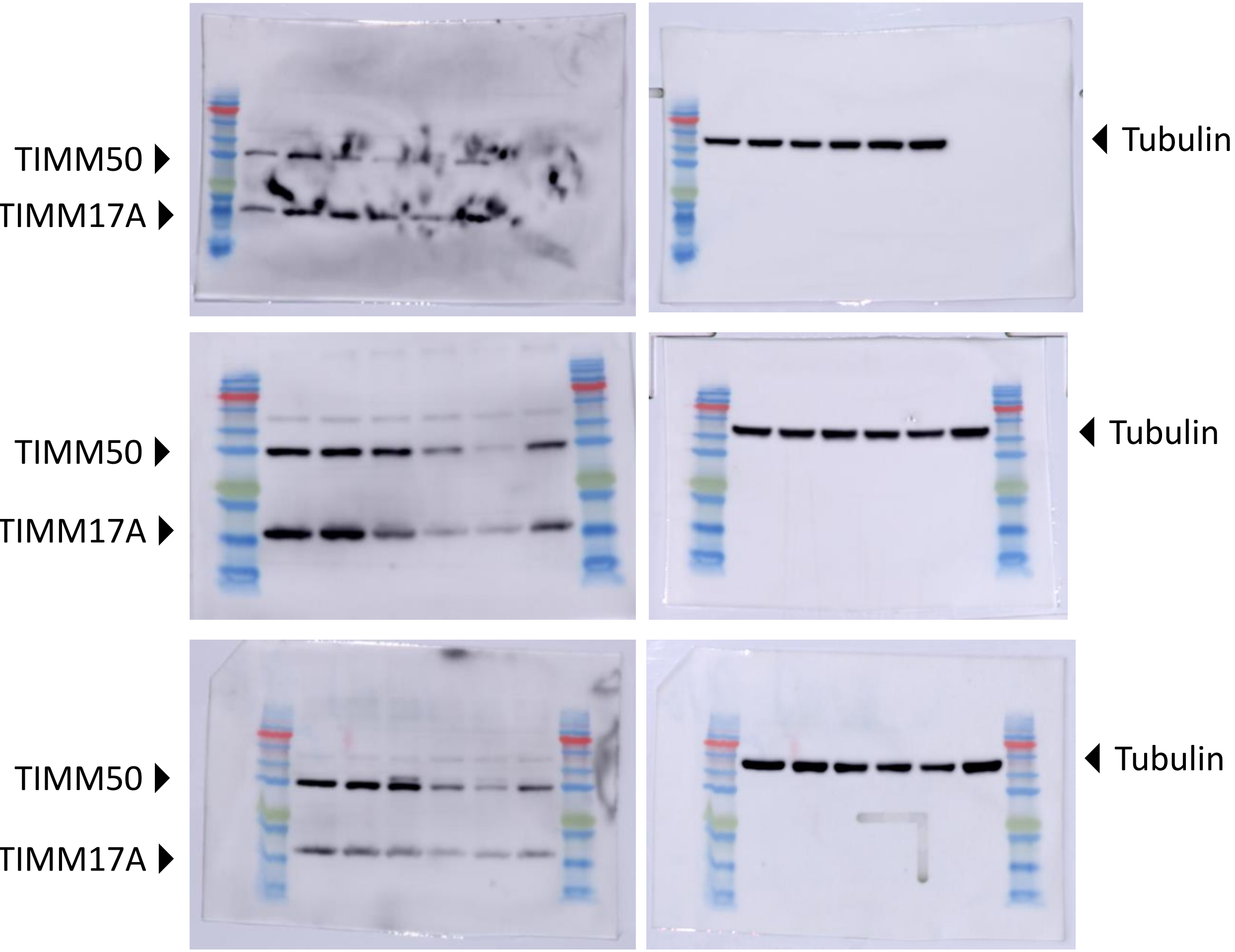

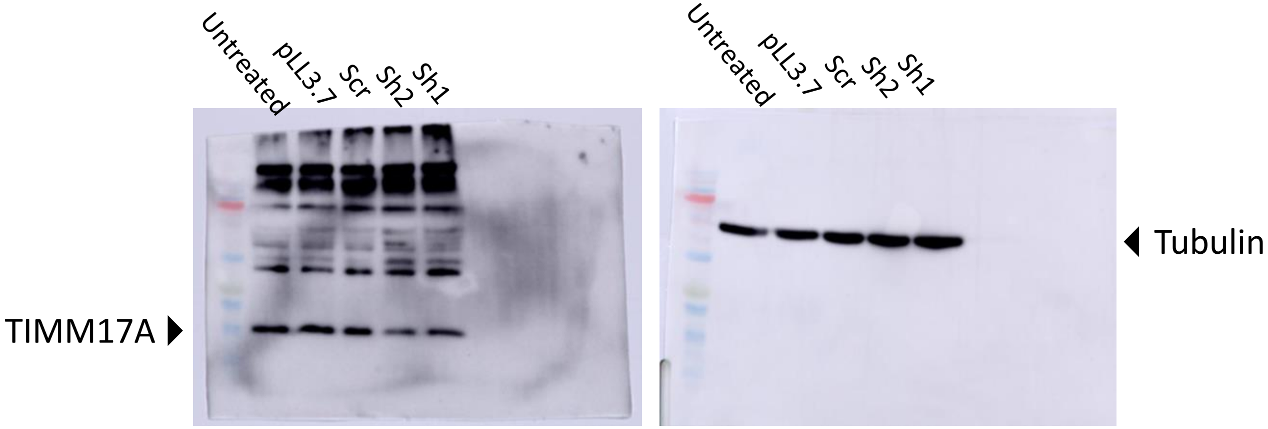

TIMM17B

(Running order is Untreated / pLL3.7 control / Scr control / Sh1 / Sh2 / Sh3)

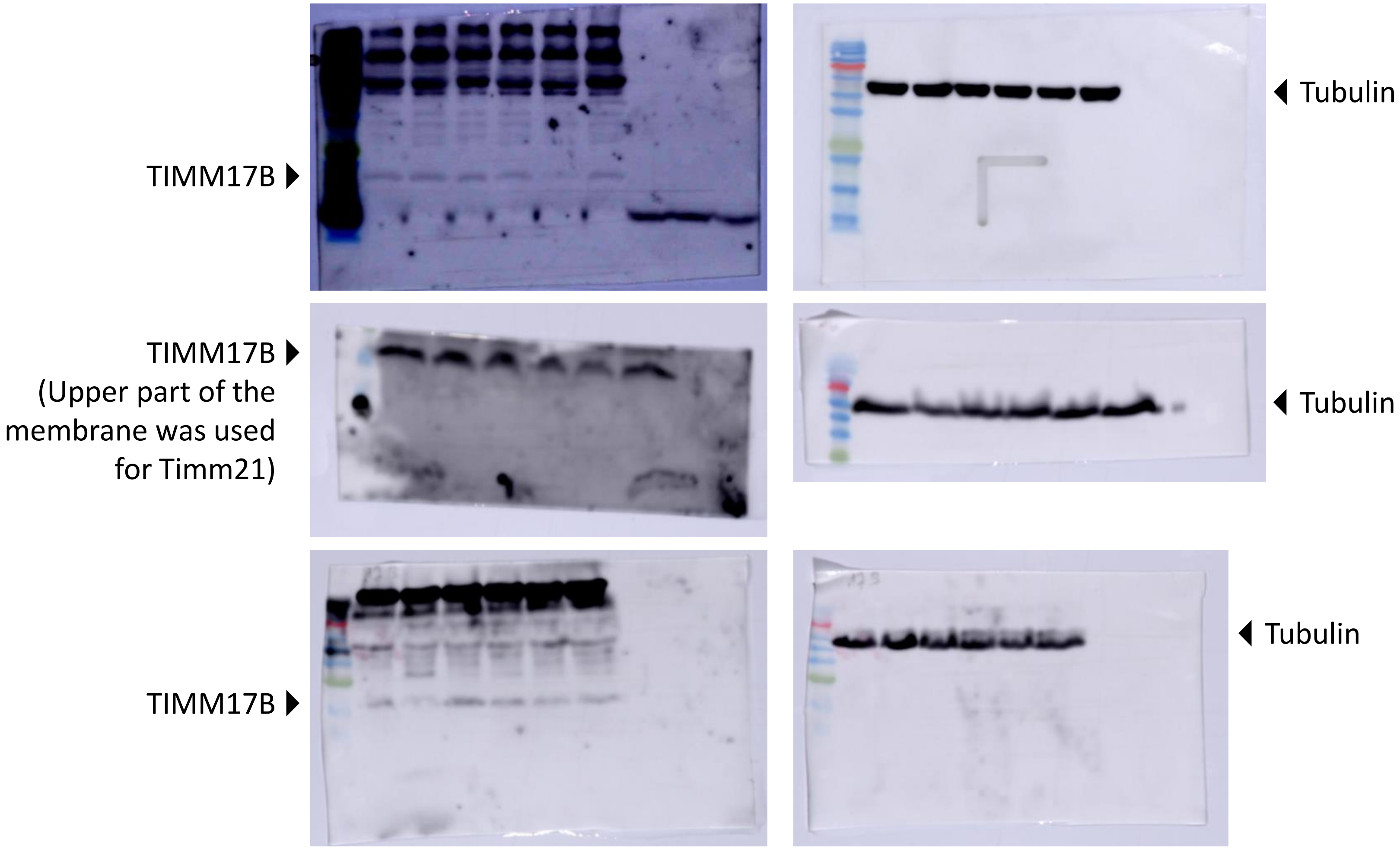

TIMM21

(Running order is Untreated / pLL3.7 control / Scr control / Sh1 / Sh2 / Sh3)

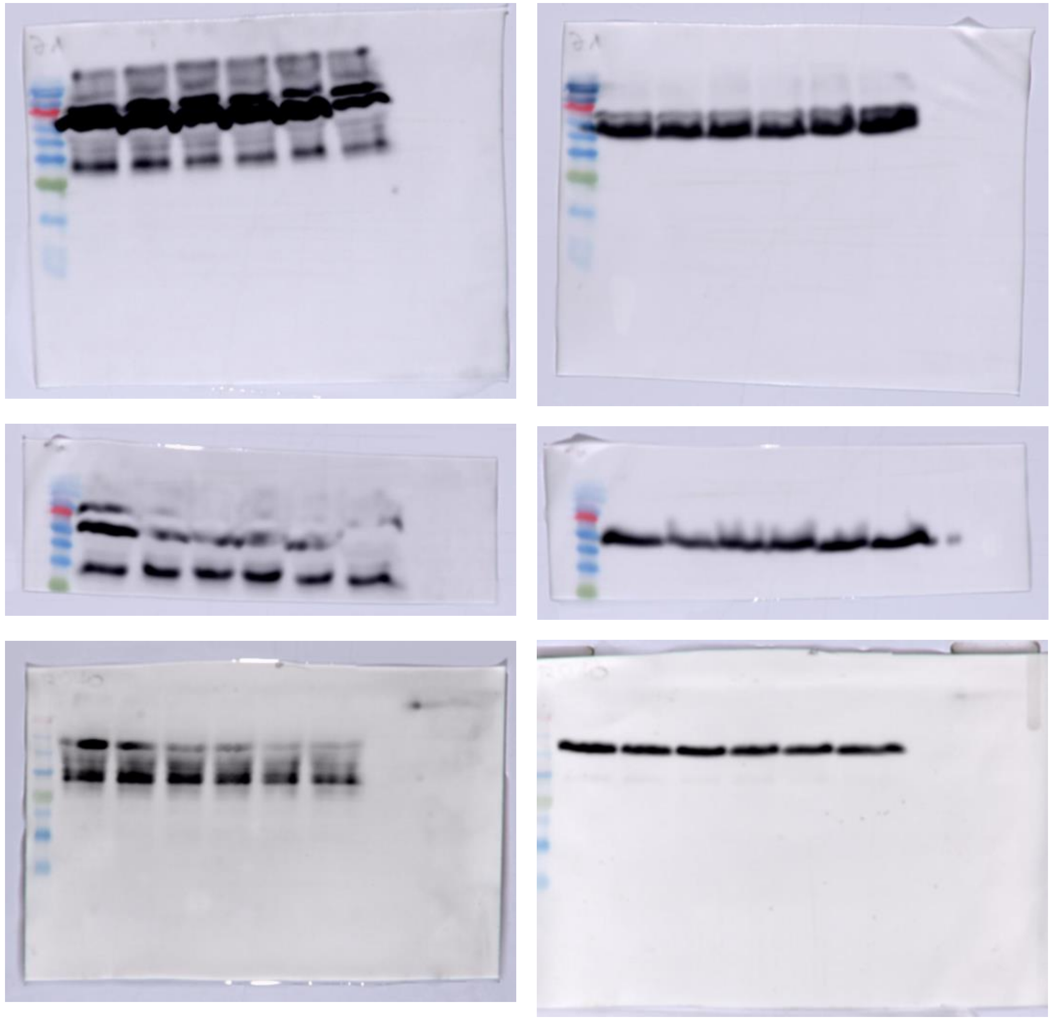

TIMM21 ▶

◀ Tubulin

TIMM21 ▶

(Bottom part of the membrane was used for TIMM17B)

◀ Tubulin

TIMM21 ▶

◀ Tubulin

TIMM44

(Running order is Untreated / pLL3.7 control / Scr control / Sh1 / Sh2 / Sh3)

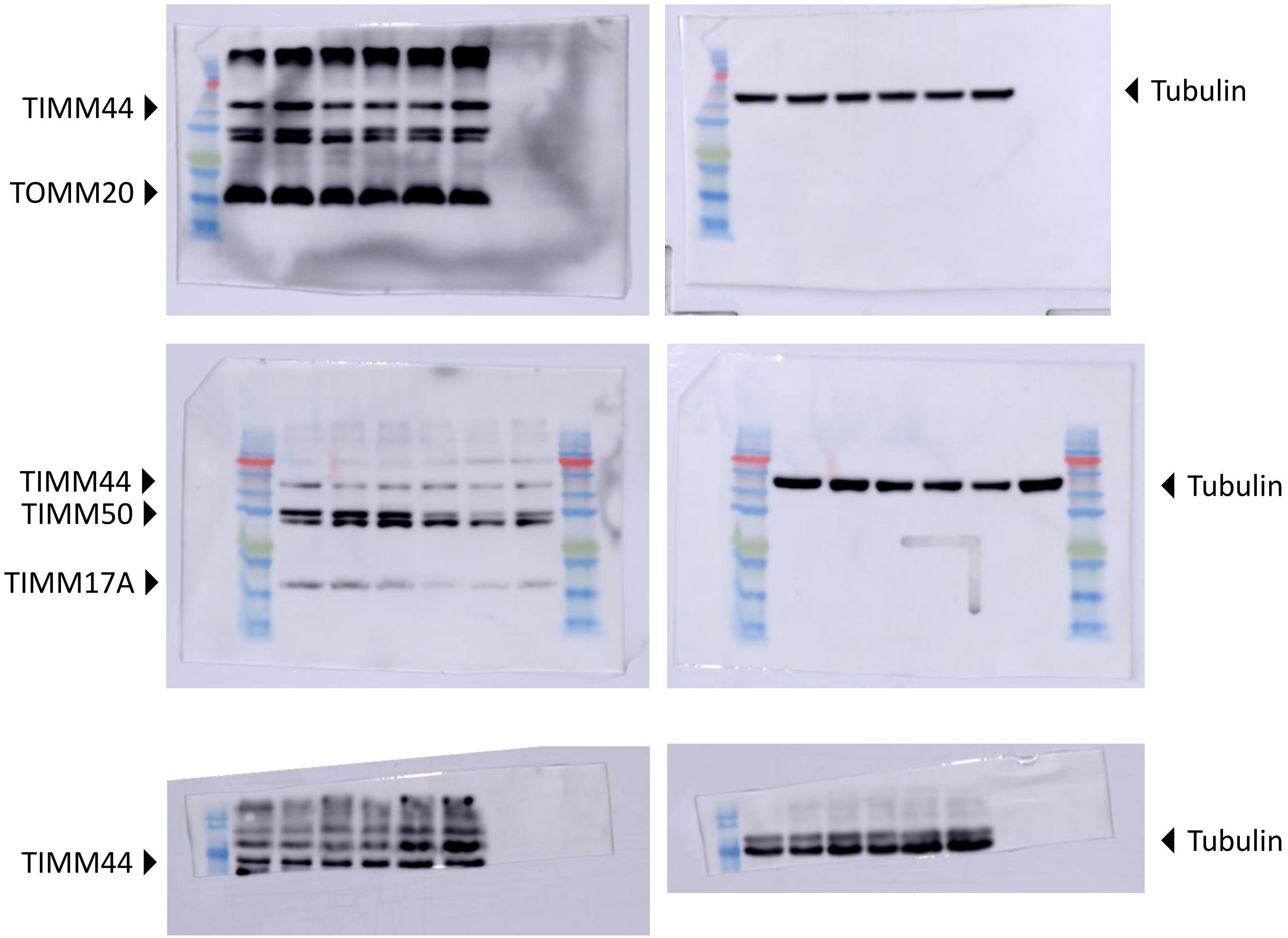

Pam16

(Running order is Untreated / pLL3.7 control / Scr control / Sh1 / Sh2 / Sh3)

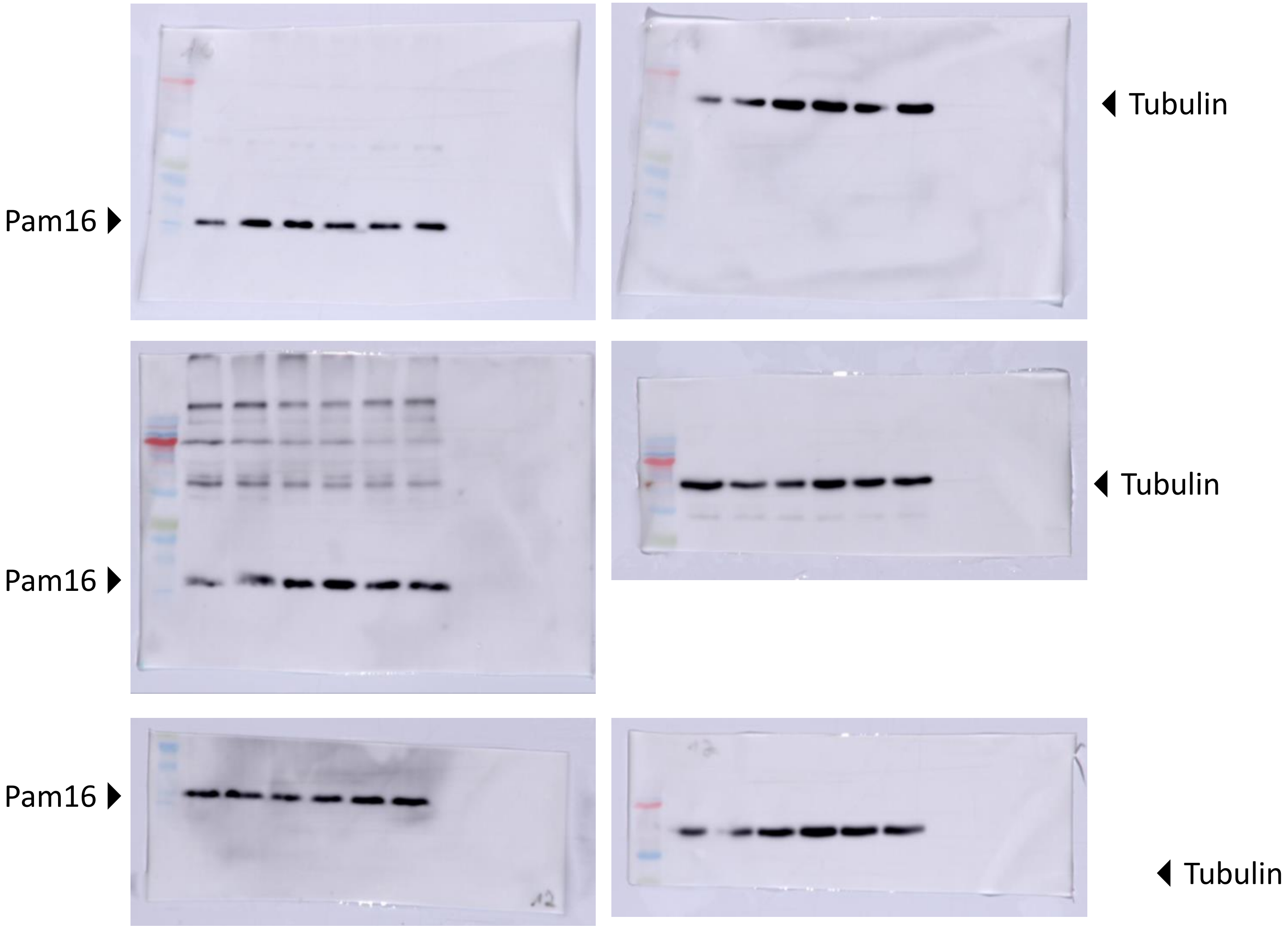

TOMM40

(Running order is Untreated / pLL3.7 control / Scr control / Sh1 / Sh2 / Sh3)

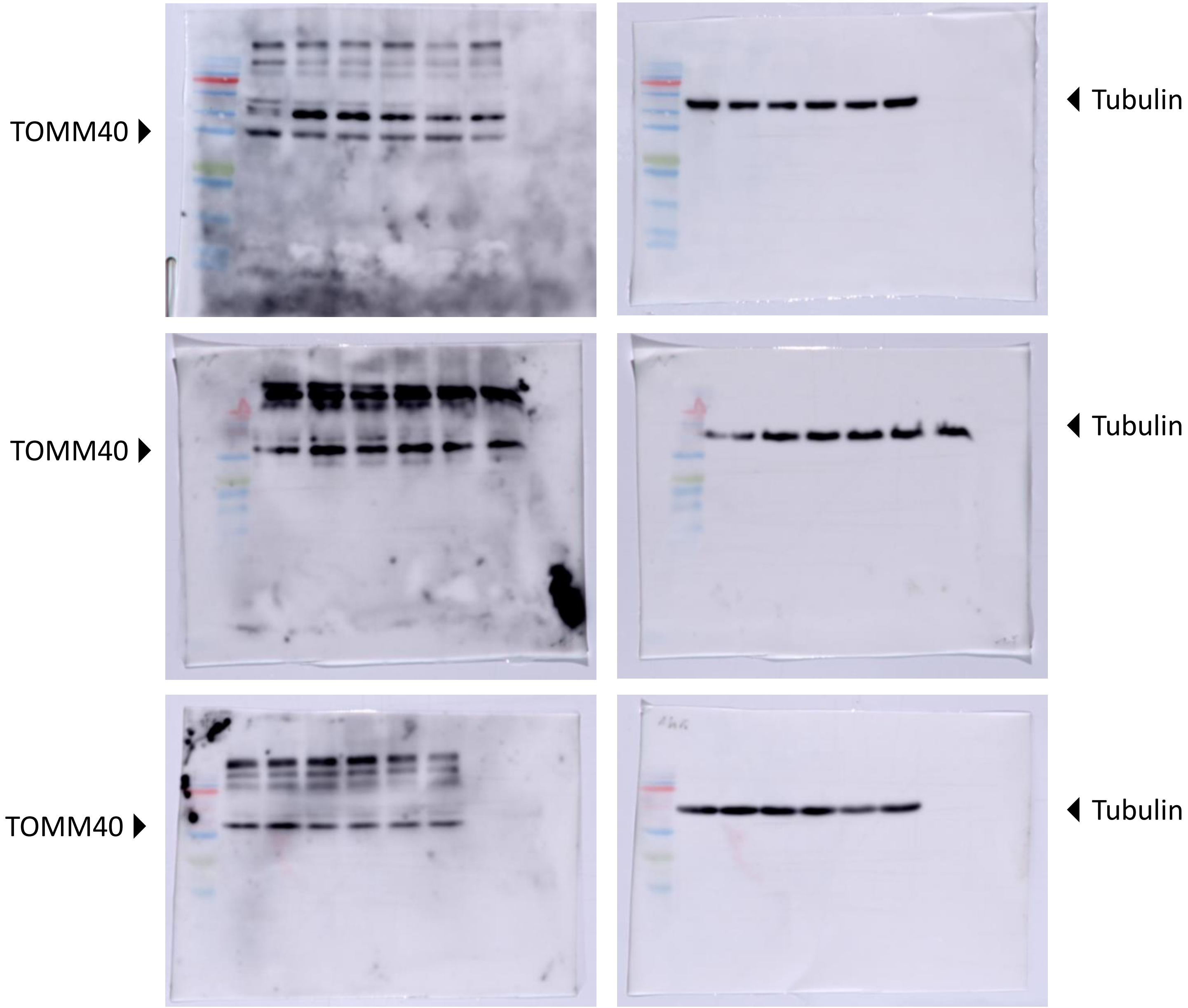

TOMM20

(Running order is Untreated / pLL3.7 control / Scr control / Sh1 / Sh2 / Sh3)

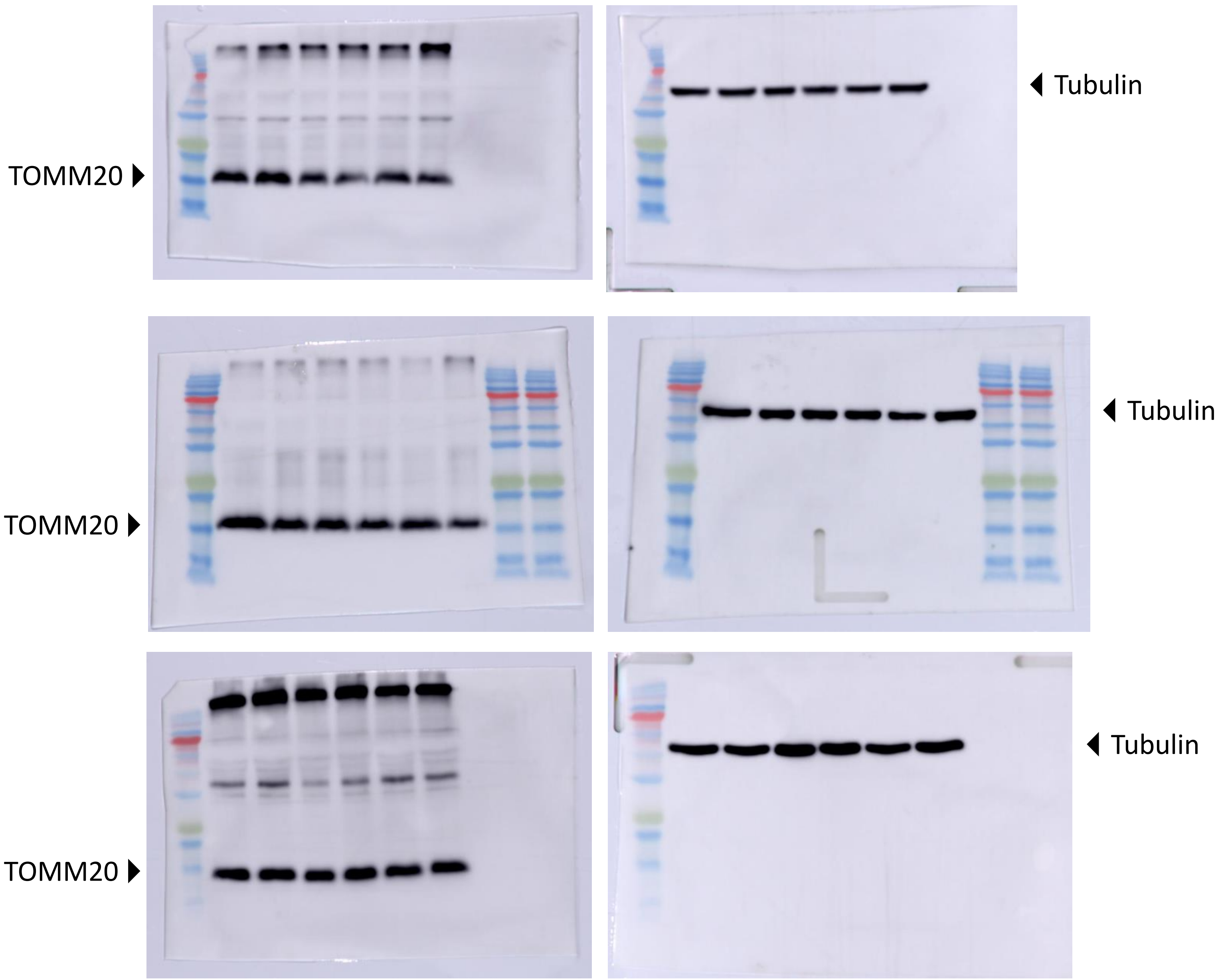

mtHsp60

(Running order is Untreated / pLL3.7 control / Scr control / Sh1 / Sh2 / Sh3)

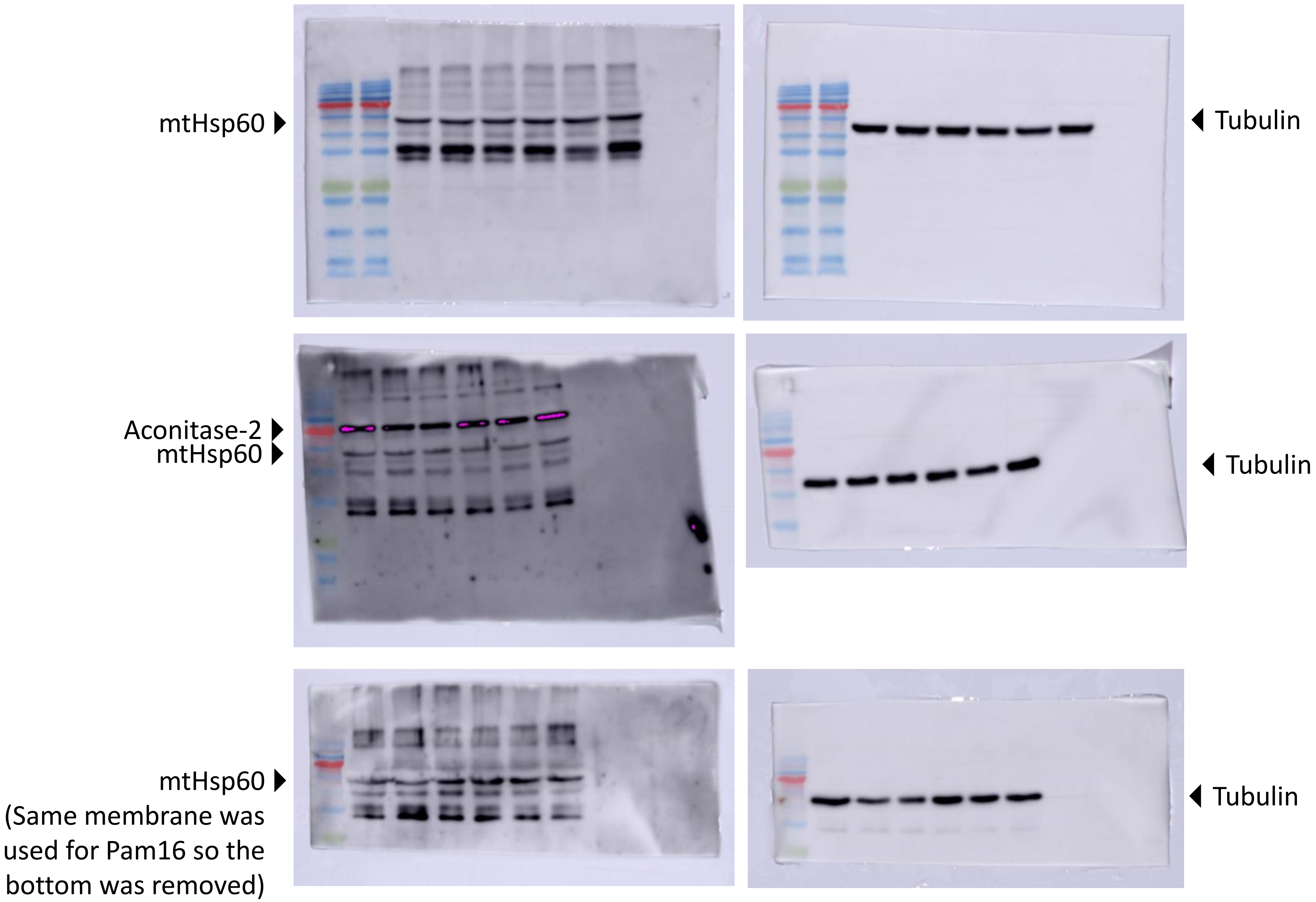

Aconitase-2

(Running order is Untreated / pLL3.7 control / Scr control / Sh1 / Sh2 / Sh3)

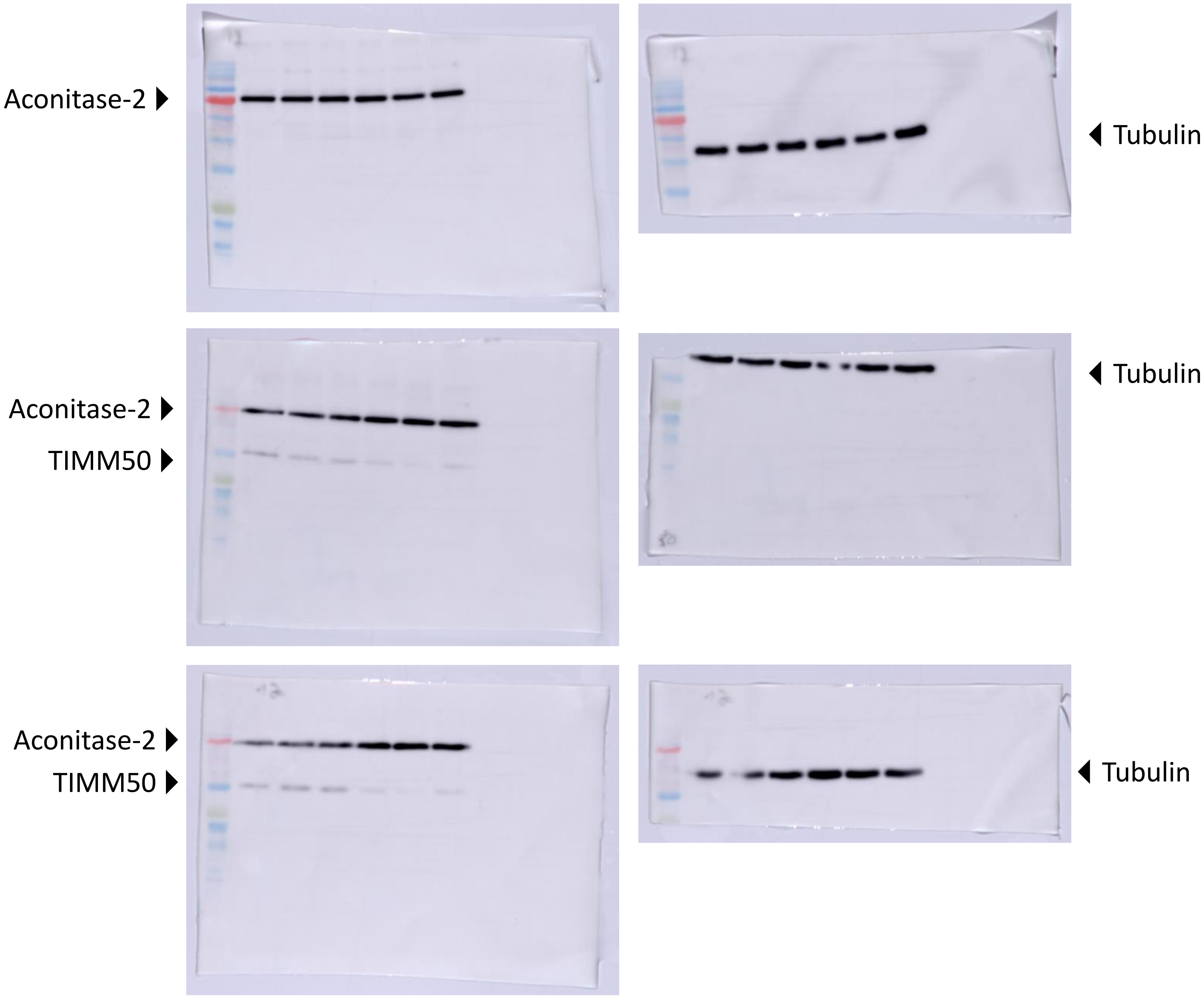

Supplement: Figure 2—source data 1. [file elife-99914-fig2-data1.zip › Figure 2-source data 1.pdf]
