# Supplementary figures and images for: Biochemical and neurophysiological effects of deficiency of the mitochondrial import protein TIMM50 (part 2 of 2)
Source: eLife. 2024 Dec 16;13:RP99914. doi: 10.7554/eLife.99914 (PMC11649234; doi:10.7554/eLife.99914)

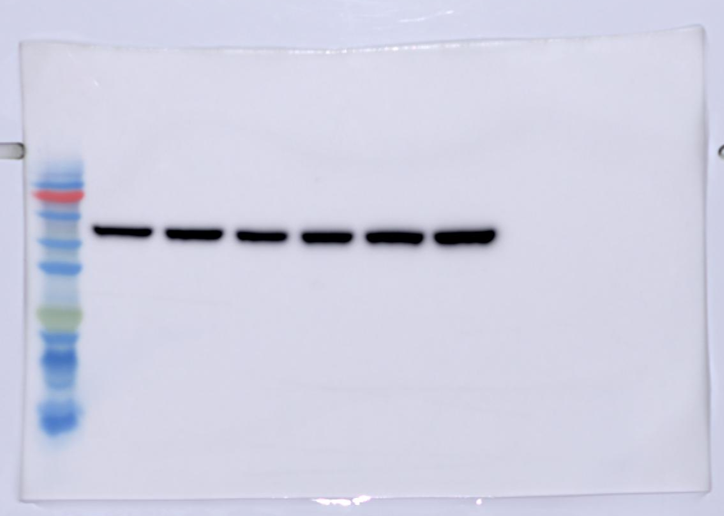

Supplement: Figure 2—source data 2. [file elife-99914-fig2-data2.zip › Figure 2-source data 2/TIMM17A Tubulin rep1.tif]

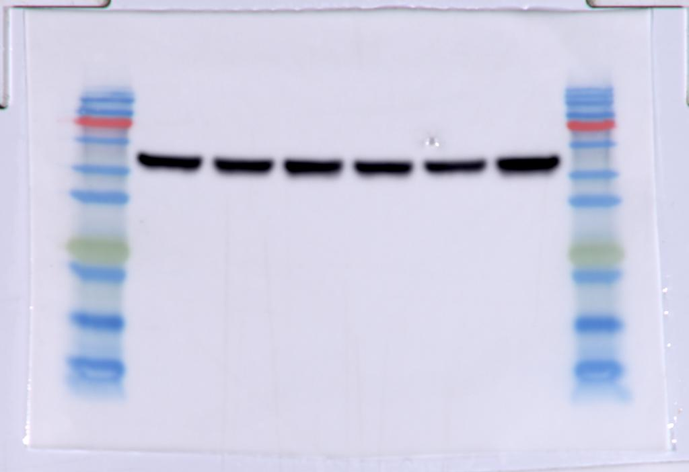

Supplement: Figure 2—source data 2. [file elife-99914-fig2-data2.zip › Figure 2-source data 2/TIMM17A Tubulin rep2.tif]

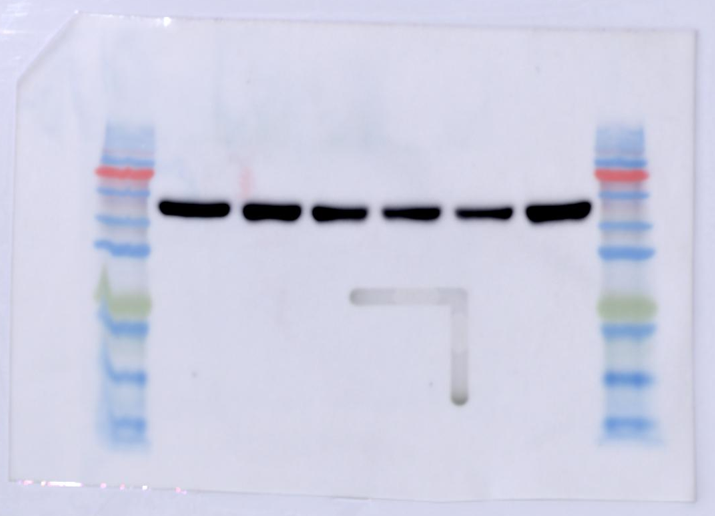

Supplement: Figure 2—source data 2. [file elife-99914-fig2-data2.zip › Figure 2-source data 2/TIMM17A Tubulin rep3.tif]

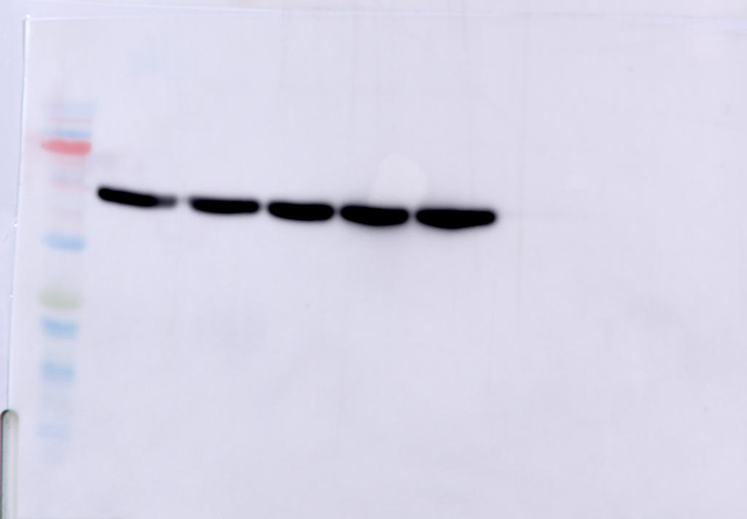

Supplement: Figure 2—source data 2. [file elife-99914-fig2-data2.zip › Figure 2-source data 2/TIMM17A Tubulin rep4.tif]

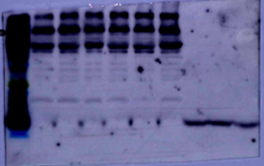

Supplement: Figure 2—source data 2. [file elife-99914-fig2-data2.zip › Figure 2-source data 2/TIMM17B rep1.tif]

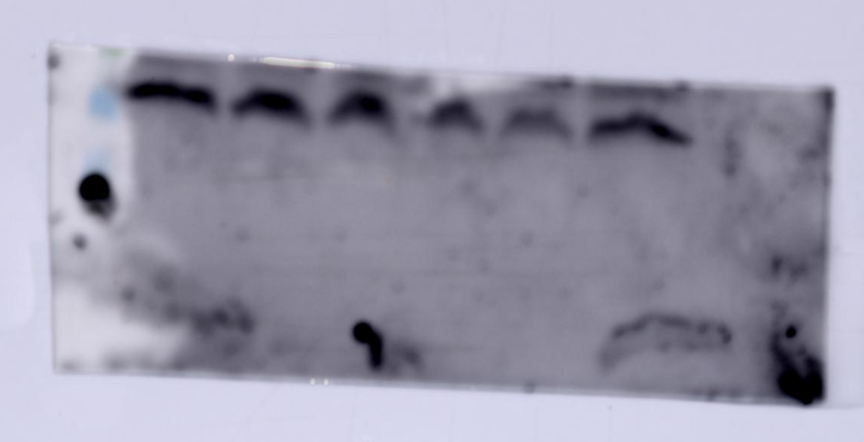

Supplement: Figure 2—source data 2. [file elife-99914-fig2-data2.zip › Figure 2-source data 2/TIMM17B rep2.tif]

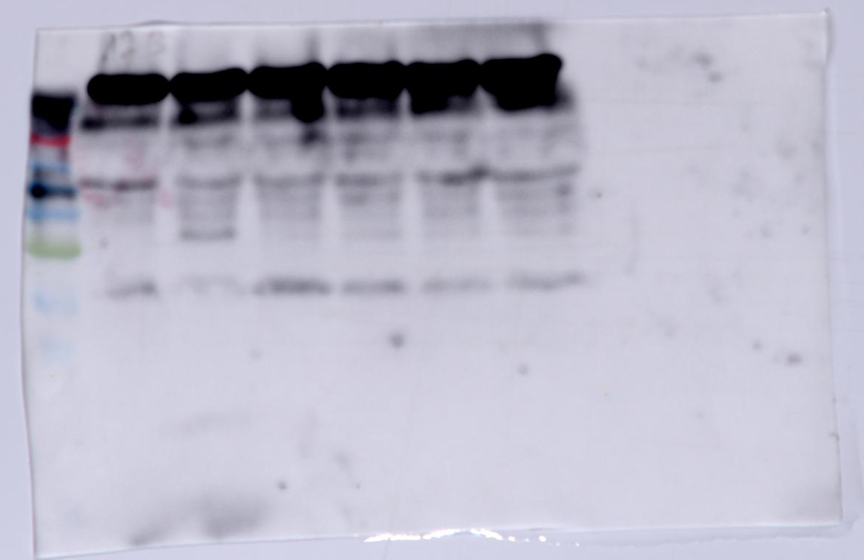

Supplement: Figure 2—source data 2. [file elife-99914-fig2-data2.zip › Figure 2-source data 2/TIMM17B rep3.tif]

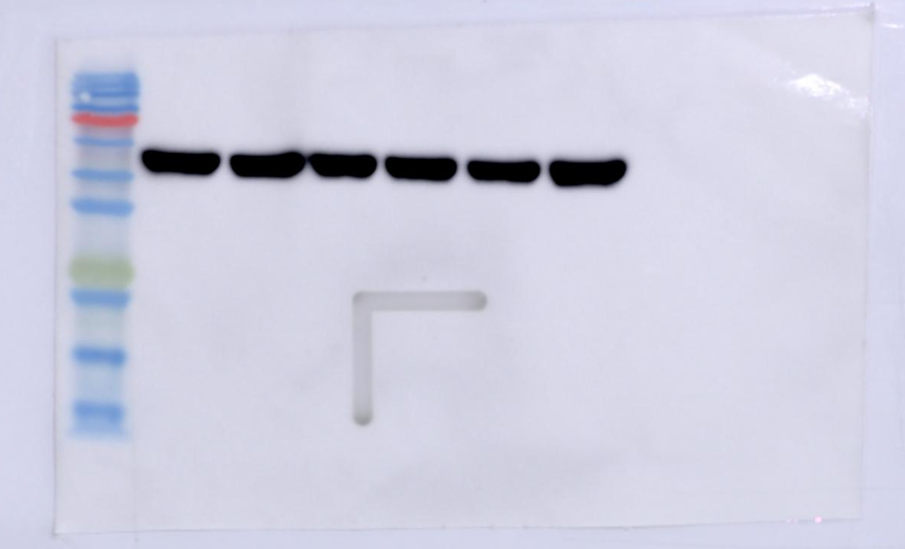

Supplement: Figure 2—source data 2. [file elife-99914-fig2-data2.zip › Figure 2-source data 2/TIMM17B Tubulin rep1.tif]

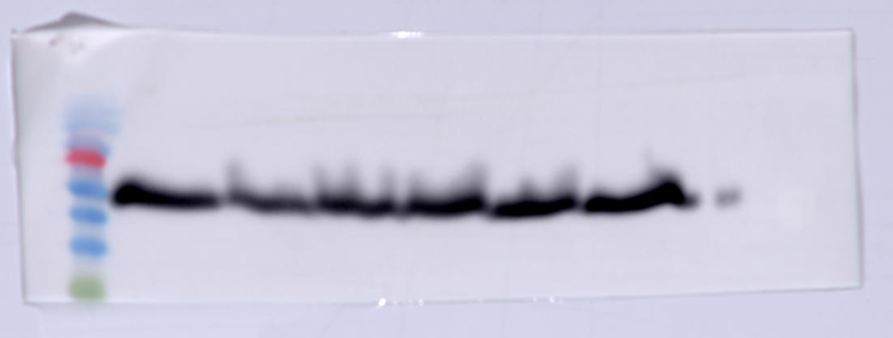

Supplement: Figure 2—source data 2. [file elife-99914-fig2-data2.zip › Figure 2-source data 2/TIMM17B Tubulin rep2.tif]

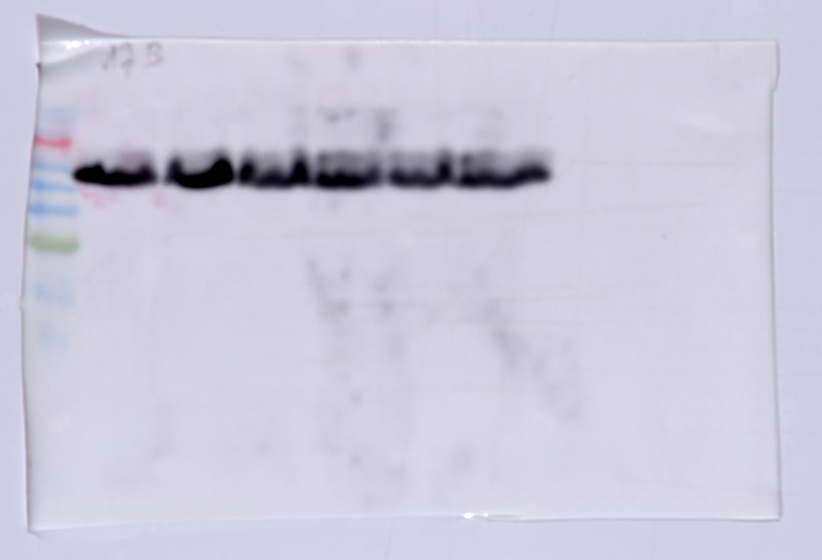

Supplement: Figure 2—source data 2. [file elife-99914-fig2-data2.zip › Figure 2-source data 2/TIMM17B Tubulin rep3.tif]

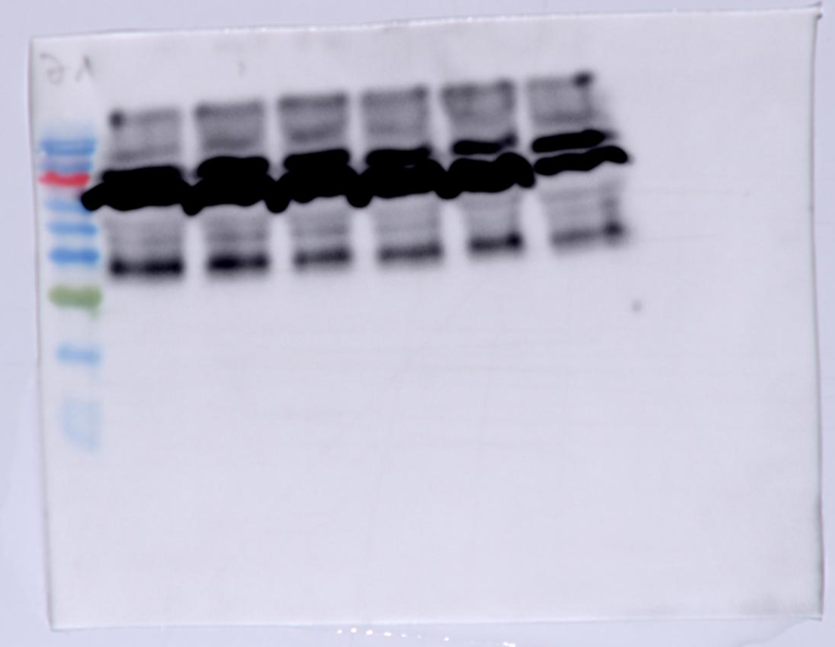

Supplement: Figure 2—source data 2. [file elife-99914-fig2-data2.zip › Figure 2-source data 2/TIMM21 rep1.tif]

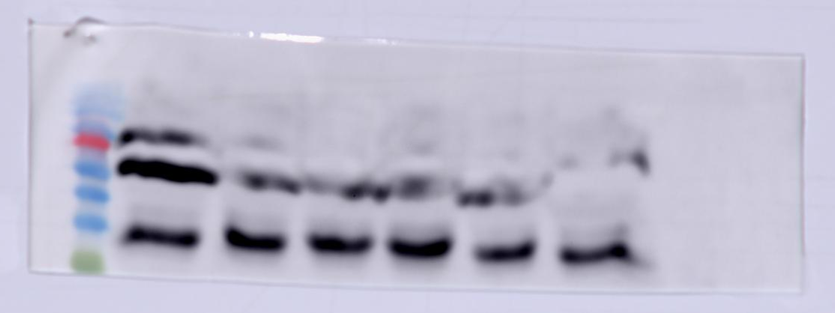

Supplement: Figure 2—source data 2. [file elife-99914-fig2-data2.zip › Figure 2-source data 2/TIMM21 rep2.tif]

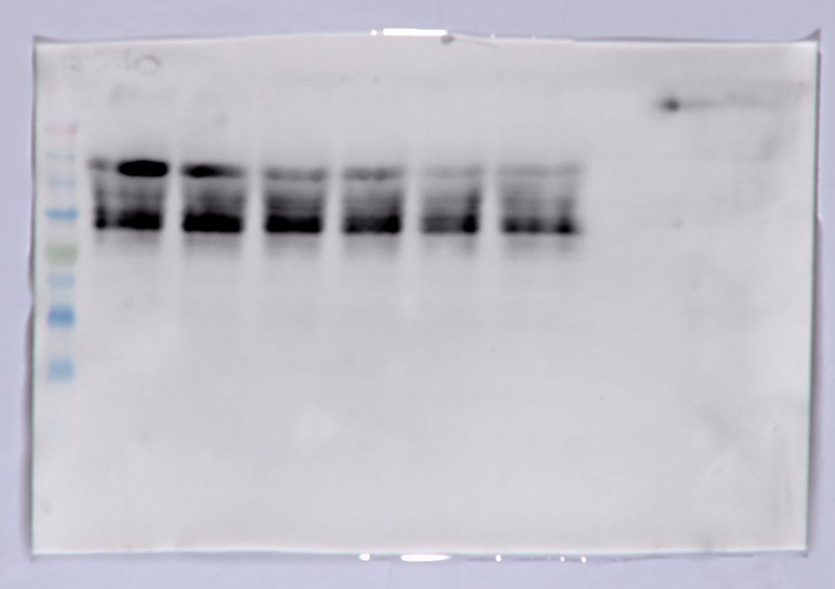

Supplement: Figure 2—source data 2. [file elife-99914-fig2-data2.zip › Figure 2-source data 2/TIMM21 rep3.tif]

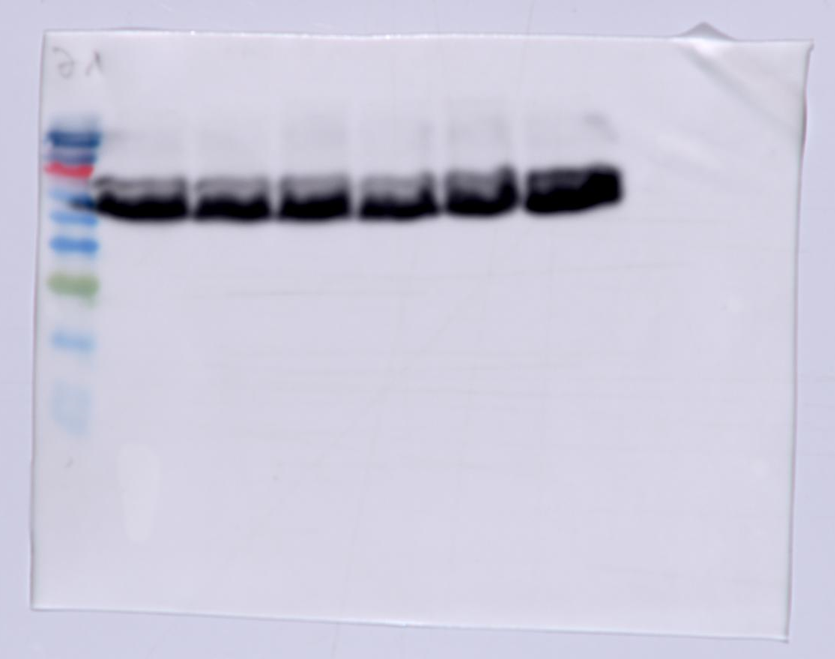

Supplement: Figure 2—source data 2. [file elife-99914-fig2-data2.zip › Figure 2-source data 2/TIMM21 Tubulin rep1.tif]

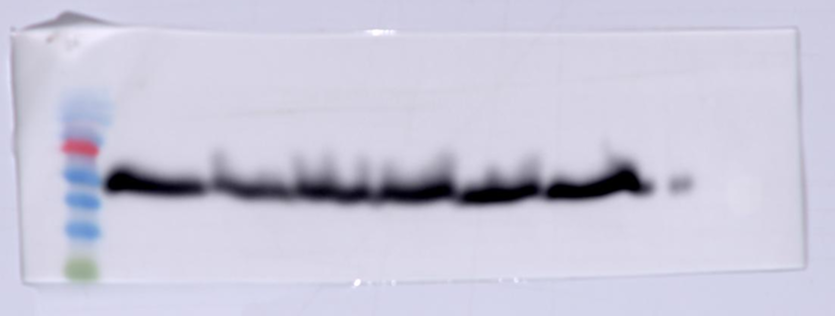

Supplement: Figure 2—source data 2. [file elife-99914-fig2-data2.zip › Figure 2-source data 2/TIMM21 Tubulin rep2.tif]

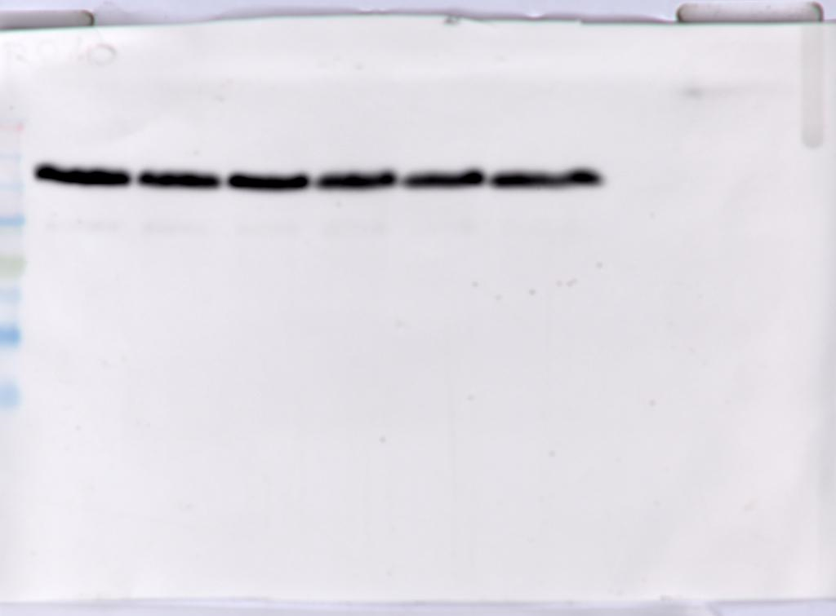

Supplement: Figure 2—source data 2. [file elife-99914-fig2-data2.zip › Figure 2-source data 2/TIMM21 Tubulin rep3.tif]

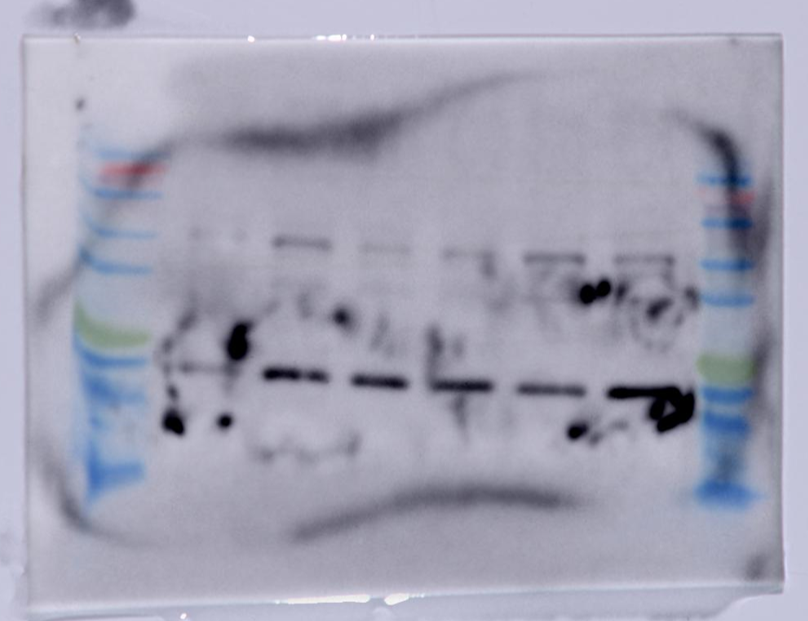

Supplement: Figure 2—source data 2. [file elife-99914-fig2-data2.zip › Figure 2-source data 2/TIMM23 rep1.tif]

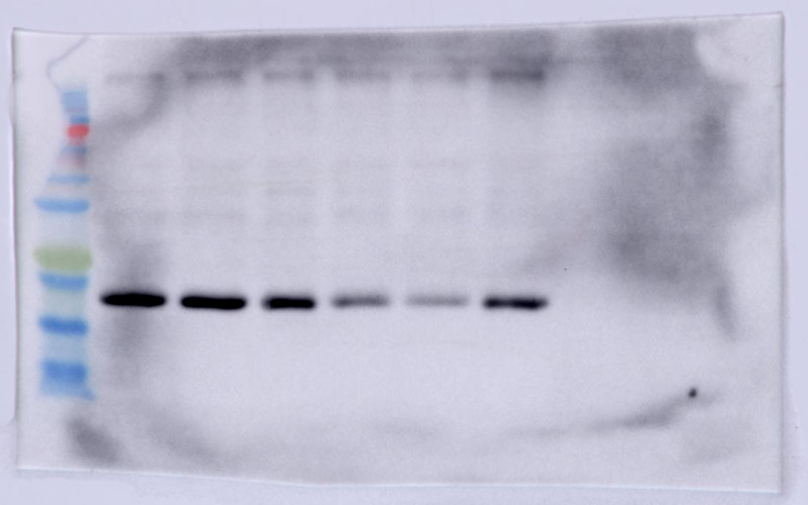

Supplement: Figure 2—source data 2. [file elife-99914-fig2-data2.zip › Figure 2-source data 2/TIMM23 rep2.tif]

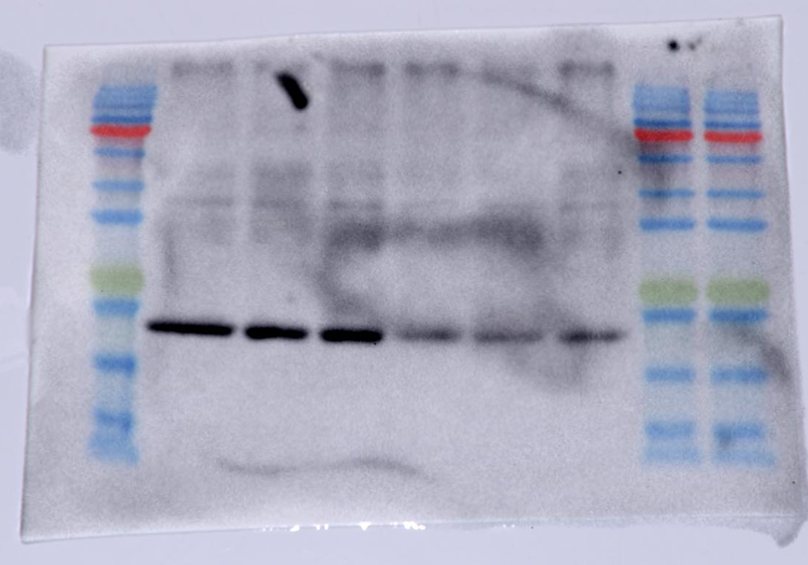

Supplement: Figure 2—source data 2. [file elife-99914-fig2-data2.zip › Figure 2-source data 2/TIMM23 rep3.tif]

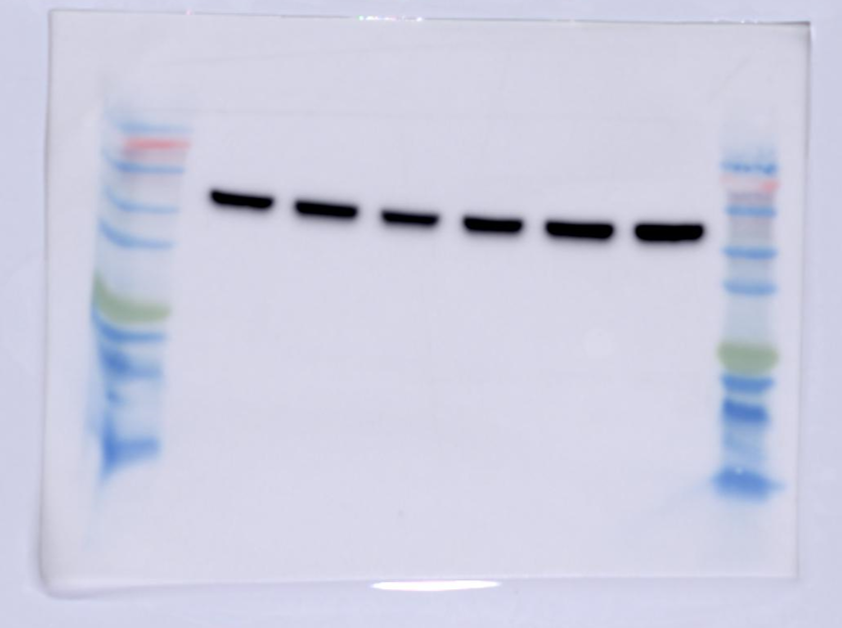

Supplement: Figure 2—source data 2. [file elife-99914-fig2-data2.zip › Figure 2-source data 2/TIMM23 Tubulin rep1.tif]

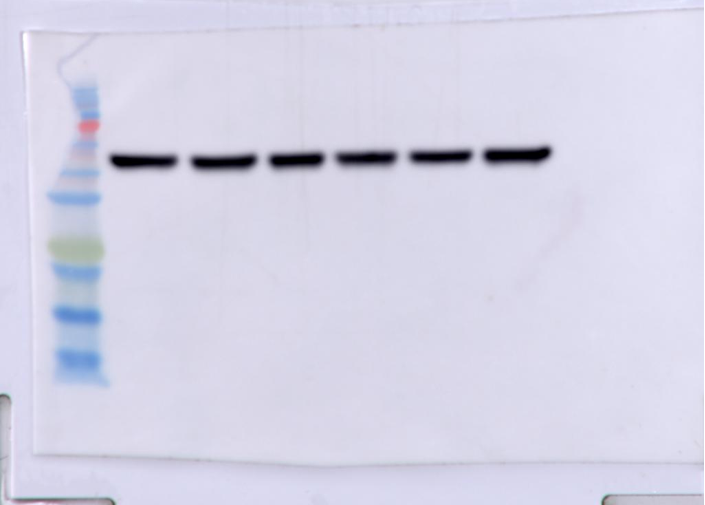

Supplement: Figure 2—source data 2. [file elife-99914-fig2-data2.zip › Figure 2-source data 2/TIMM23 Tubulin rep2.tif]

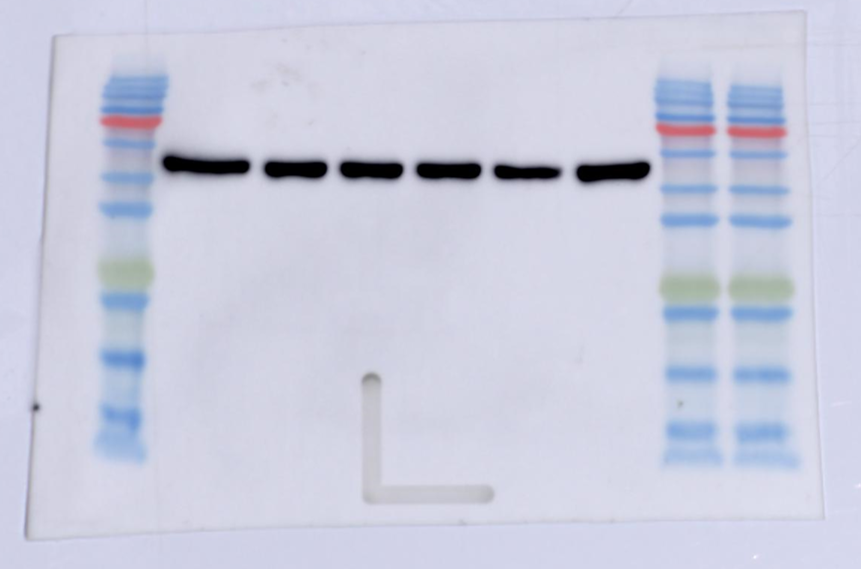

Supplement: Figure 2—source data 2. [file elife-99914-fig2-data2.zip › Figure 2-source data 2/TIMM23 Tubulin rep3.tif]

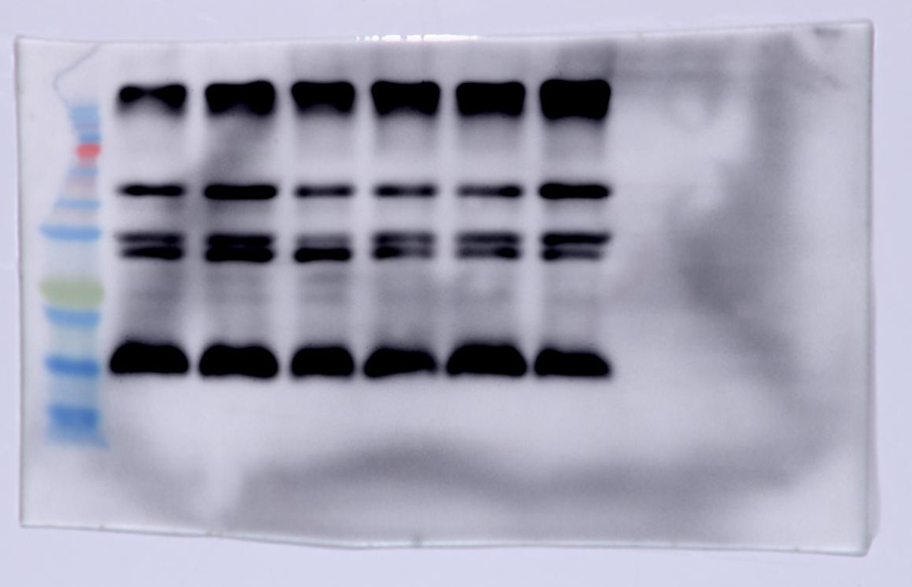

Supplement: Figure 2—source data 2. [file elife-99914-fig2-data2.zip › Figure 2-source data 2/TIMM44 rep1.tif]

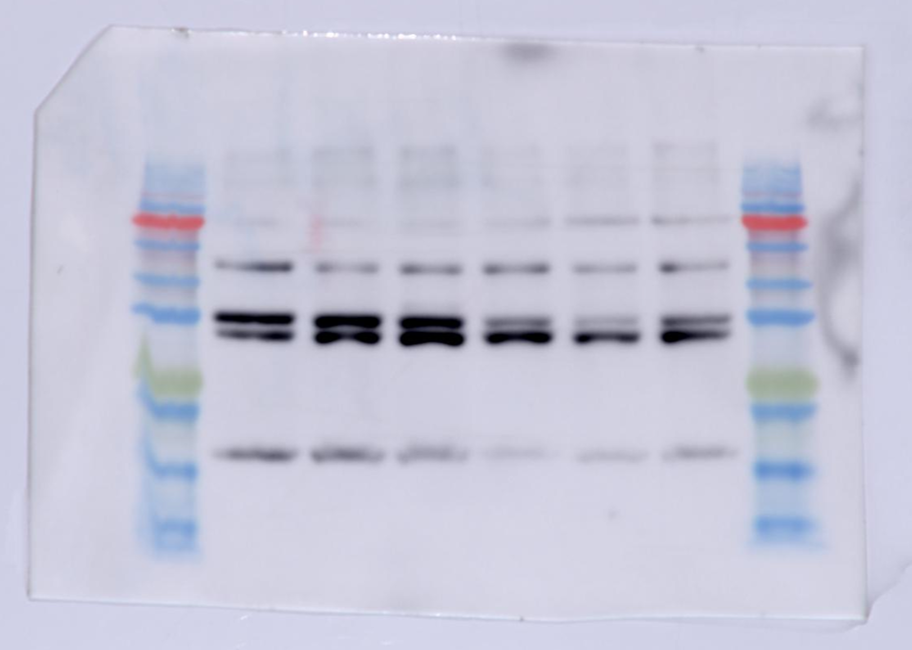

Supplement: Figure 2—source data 2. [file elife-99914-fig2-data2.zip › Figure 2-source data 2/TIMM44 rep2.tif]

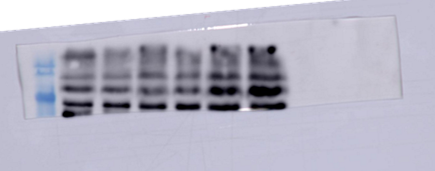

Supplement: Figure 2—source data 2. [file elife-99914-fig2-data2.zip › Figure 2-source data 2/TIMM44 rep3.tif]

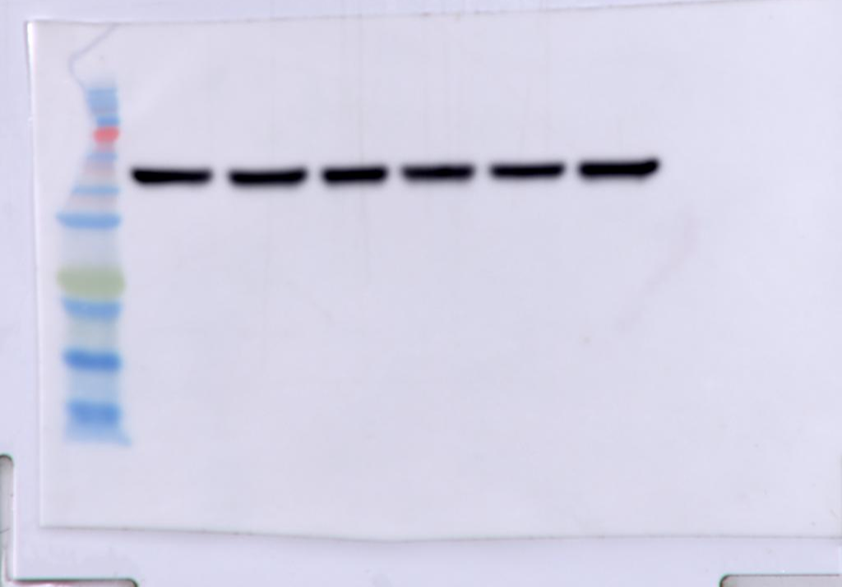

Supplement: Figure 2—source data 2. [file elife-99914-fig2-data2.zip › Figure 2-source data 2/TIMM44 Tubulin rep1.tif]

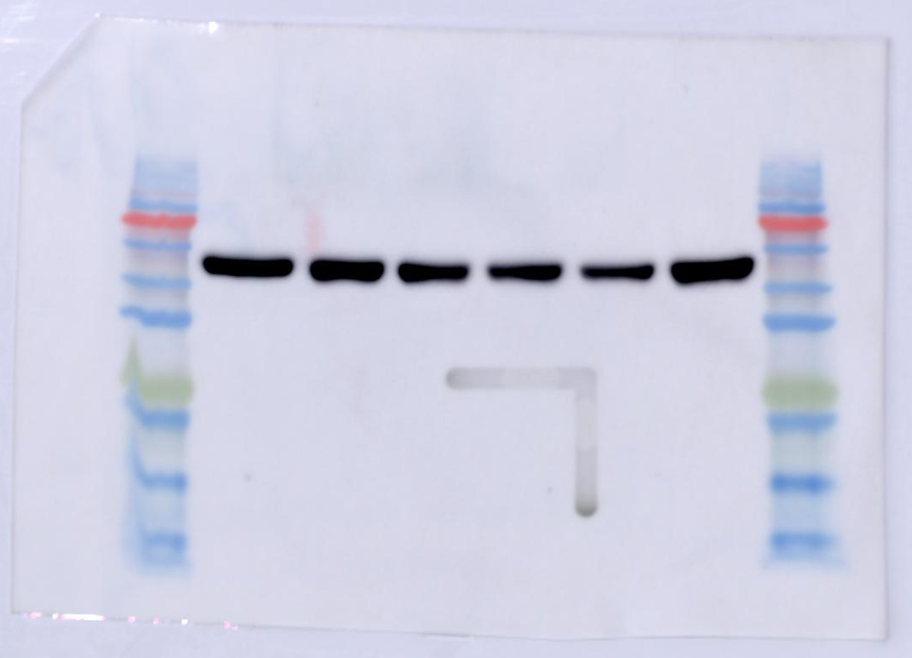

Supplement: Figure 2—source data 2. [file elife-99914-fig2-data2.zip › Figure 2-source data 2/TIMM44 Tubulin rep2.tif]

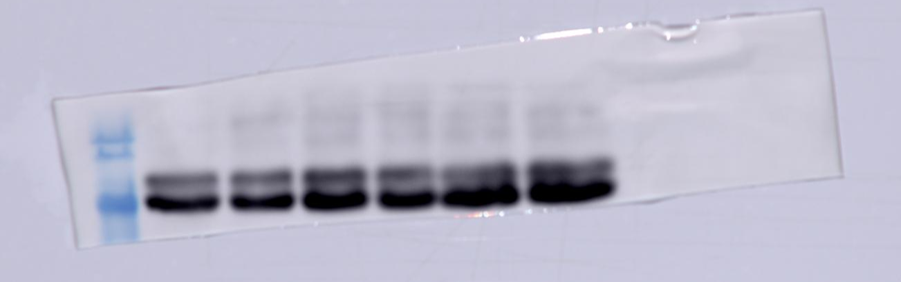

Supplement: Figure 2—source data 2. [file elife-99914-fig2-data2.zip › Figure 2-source data 2/TIMM44 Tubulin rep3.tif]

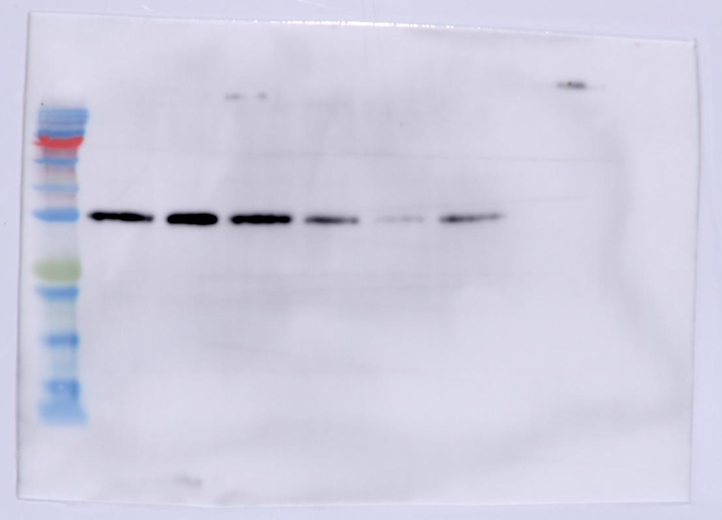

Supplement: Figure 2—source data 2. [file elife-99914-fig2-data2.zip › Figure 2-source data 2/TIMM50 rep1.tif]

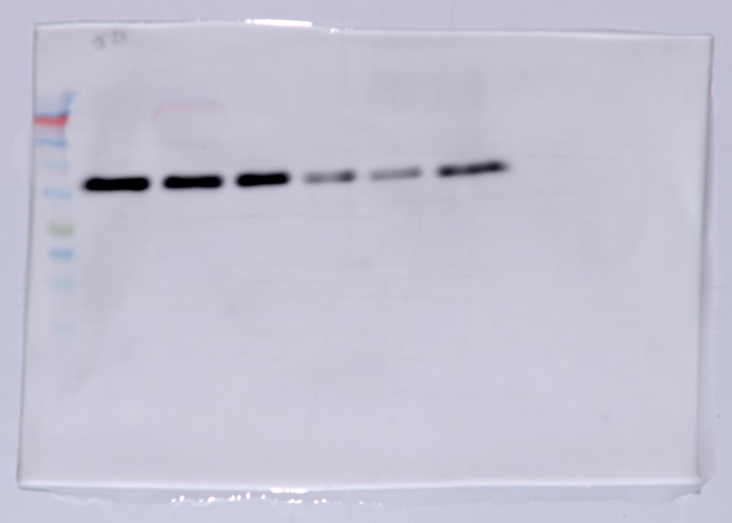

Supplement: Figure 2—source data 2. [file elife-99914-fig2-data2.zip › Figure 2-source data 2/TIMM50 rep10.tif]

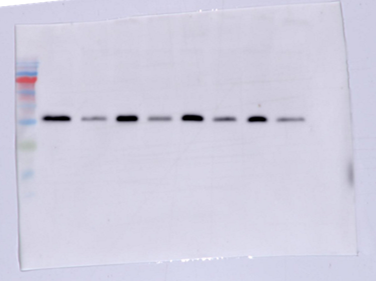

Supplement: Figure 2—source data 2. [file elife-99914-fig2-data2.zip › Figure 2-source data 2/TIMM50 rep11-14.tif]

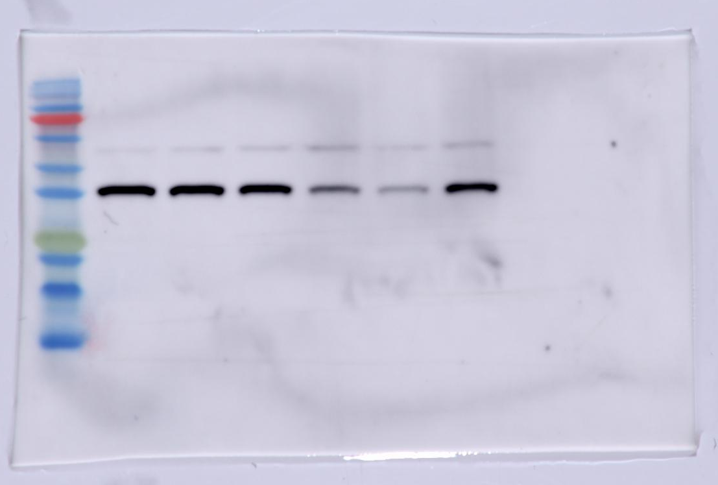

Supplement: Figure 2—source data 2. [file elife-99914-fig2-data2.zip › Figure 2-source data 2/TIMM50 rep2.tif]

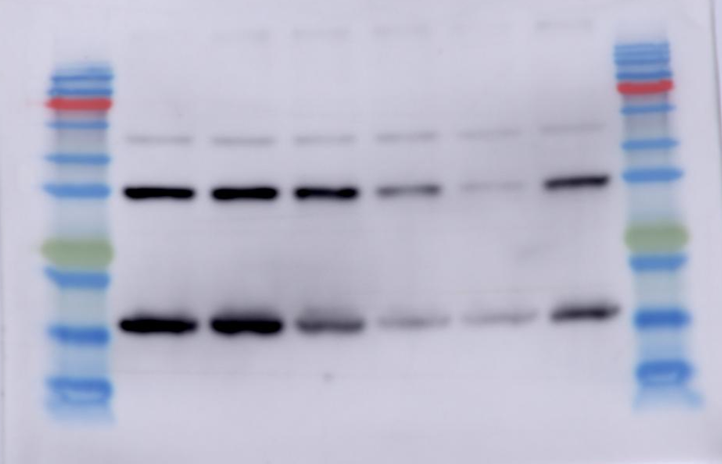

Supplement: Figure 2—source data 2. [file elife-99914-fig2-data2.zip › Figure 2-source data 2/TIMM50 rep3.tif]

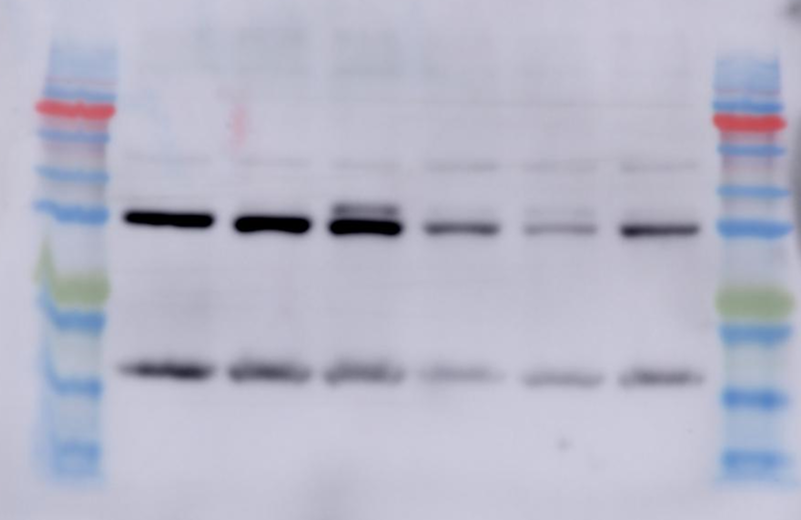

Supplement: Figure 2—source data 2. [file elife-99914-fig2-data2.zip › Figure 2-source data 2/TIMM50 rep4.tif]

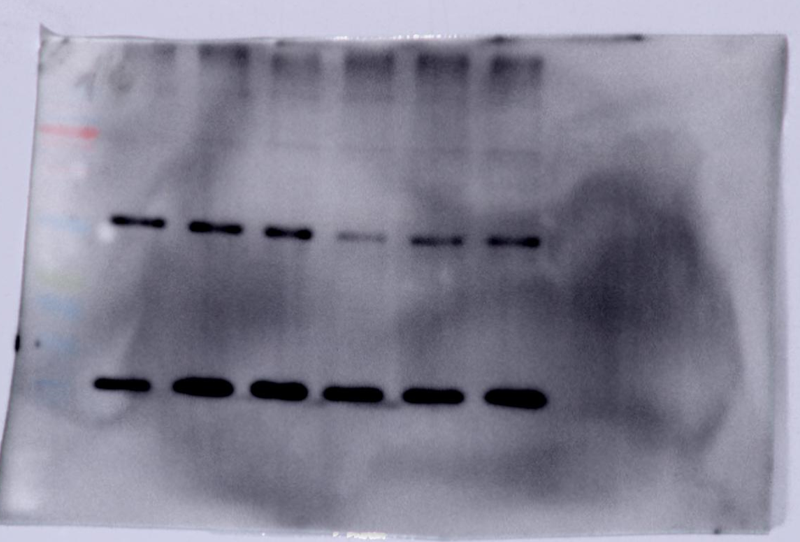

Supplement: Figure 2—source data 2. [file elife-99914-fig2-data2.zip › Figure 2-source data 2/TIMM50 rep5.tif]

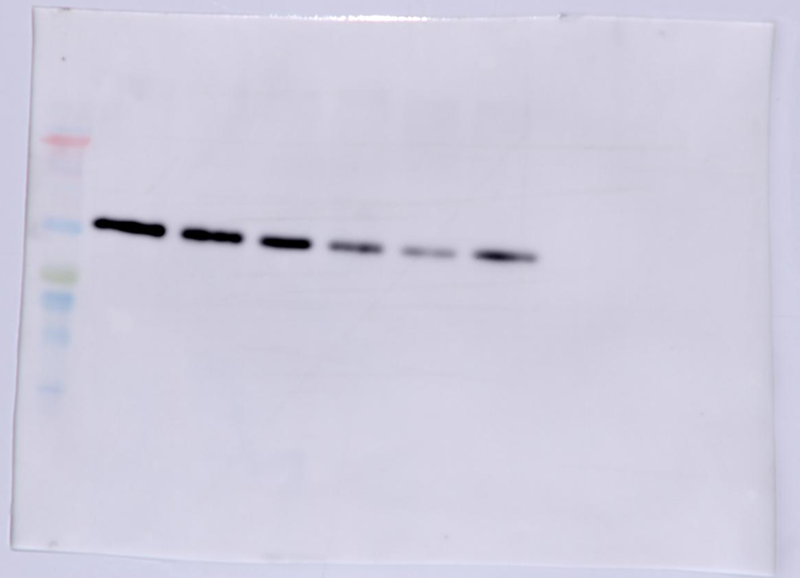

Supplement: Figure 2—source data 2. [file elife-99914-fig2-data2.zip › Figure 2-source data 2/TIMM50 rep6.tif]

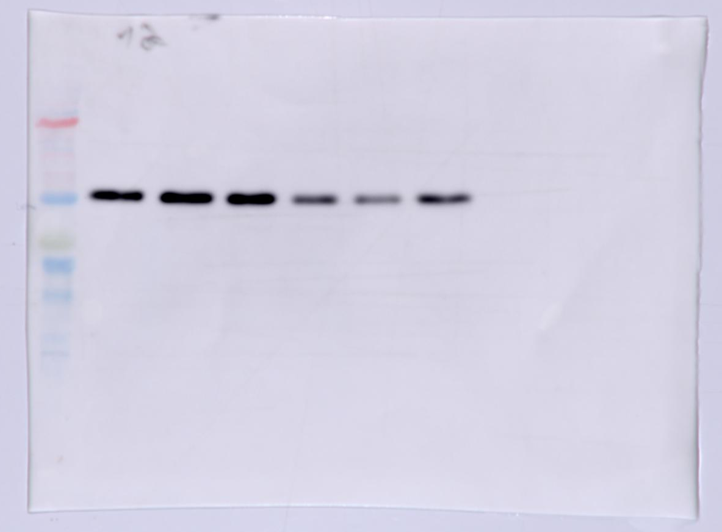

Supplement: Figure 2—source data 2. [file elife-99914-fig2-data2.zip › Figure 2-source data 2/TIMM50 rep7.tif]

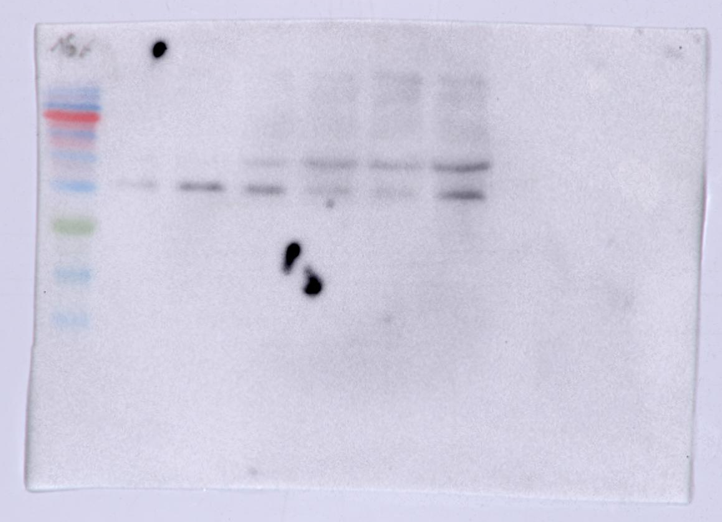

Supplement: Figure 2—source data 2. [file elife-99914-fig2-data2.zip › Figure 2-source data 2/TIMM50 rep8.tif]

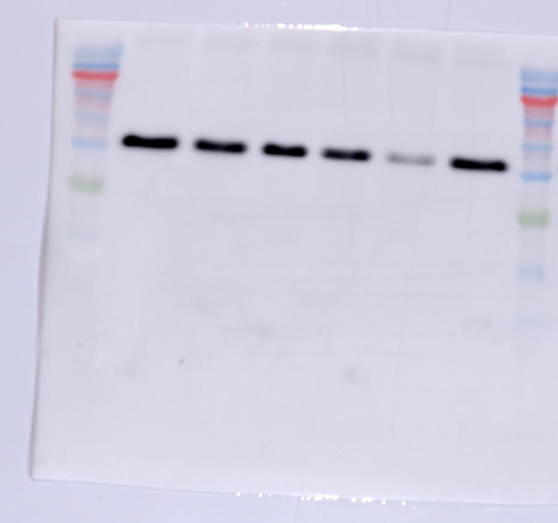

Supplement: Figure 2—source data 2. [file elife-99914-fig2-data2.zip › Figure 2-source data 2/TIMM50 rep9.tif]

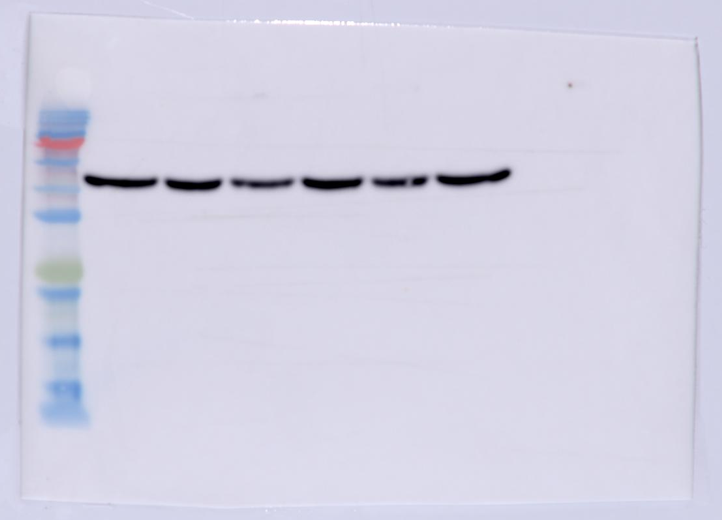

Supplement: Figure 2—source data 2. [file elife-99914-fig2-data2.zip › Figure 2-source data 2/TIMM50 Tubulin rep1.tif]

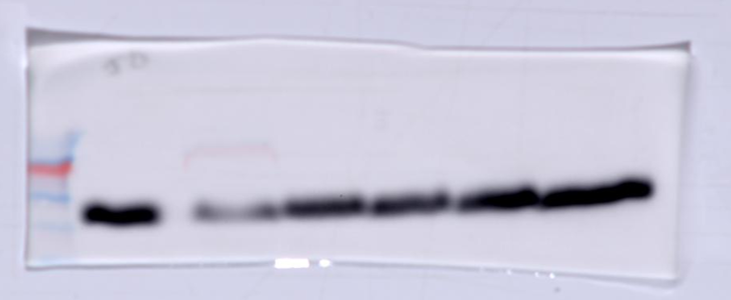

Supplement: Figure 2—source data 2. [file elife-99914-fig2-data2.zip › Figure 2-source data 2/TIMM50 Tubulin rep10.tif]

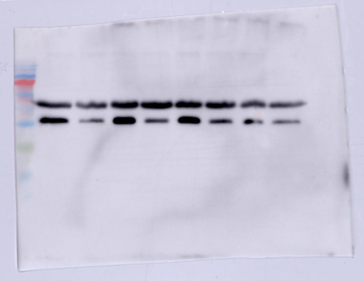

Supplement: Figure 2—source data 2. [file elife-99914-fig2-data2.zip › Figure 2-source data 2/TIMM50 Tubulin rep11-14.tif]

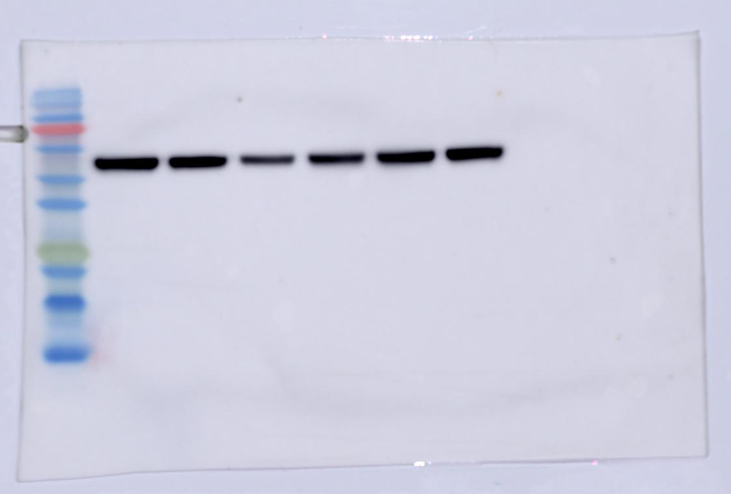

Supplement: Figure 2—source data 2. [file elife-99914-fig2-data2.zip › Figure 2-source data 2/TIMM50 Tubulin rep2.tif]

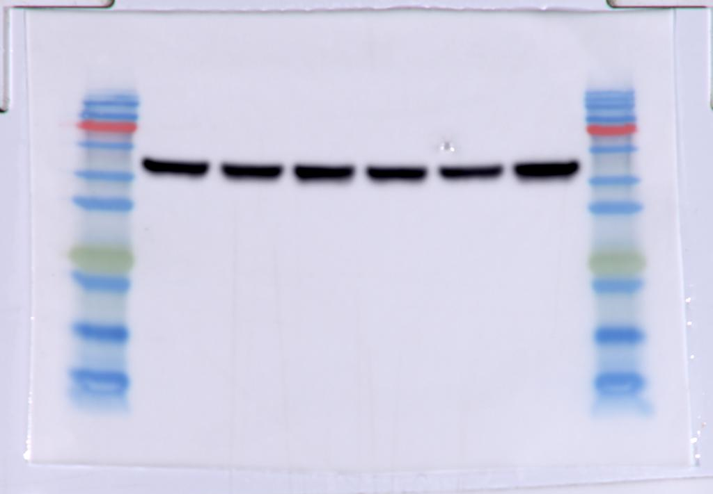

Supplement: Figure 2—source data 2. [file elife-99914-fig2-data2.zip › Figure 2-source data 2/TIMM50 Tubulin rep3.tif]

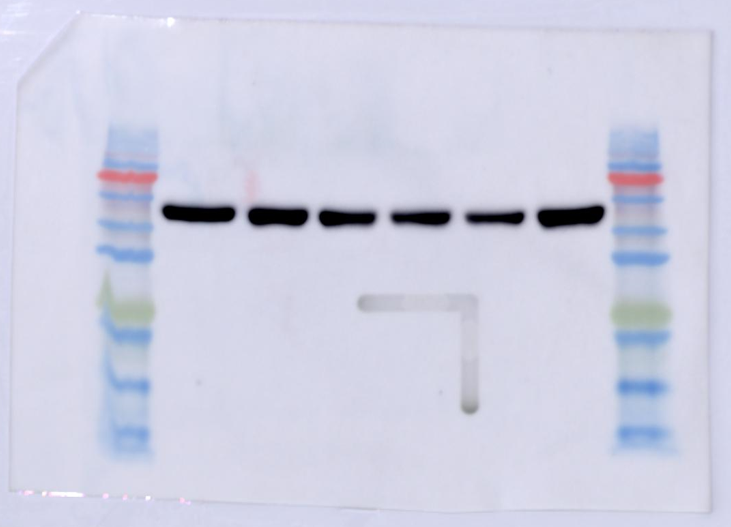

Supplement: Figure 2—source data 2. [file elife-99914-fig2-data2.zip › Figure 2-source data 2/TIMM50 Tubulin rep4.tif]

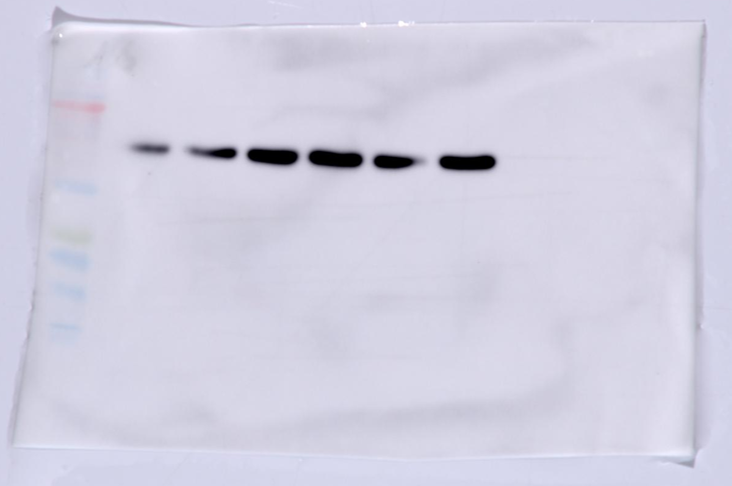

Supplement: Figure 2—source data 2. [file elife-99914-fig2-data2.zip › Figure 2-source data 2/TIMM50 Tubulin rep5.tif]

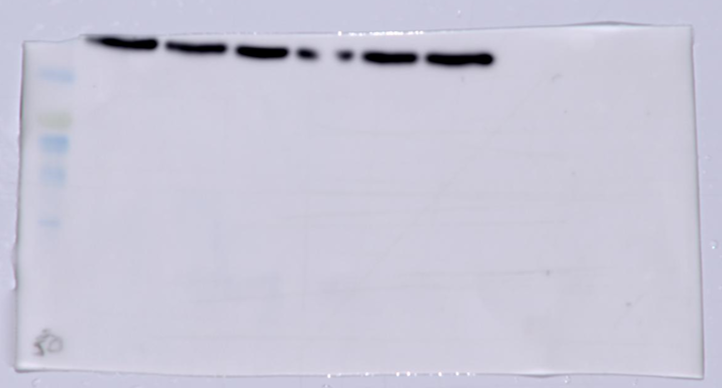

Supplement: Figure 2—source data 2. [file elife-99914-fig2-data2.zip › Figure 2-source data 2/TIMM50 Tubulin rep6.tif]

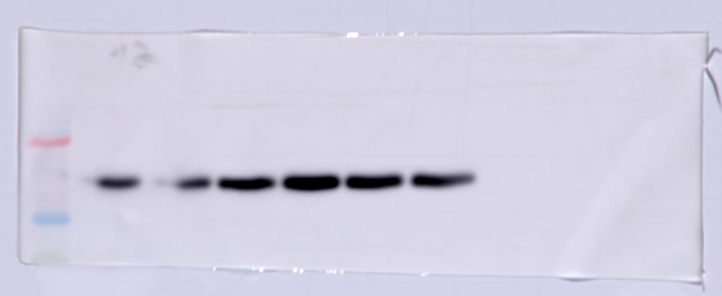

Supplement: Figure 2—source data 2. [file elife-99914-fig2-data2.zip › Figure 2-source data 2/TIMM50 Tubulin rep7.tif]

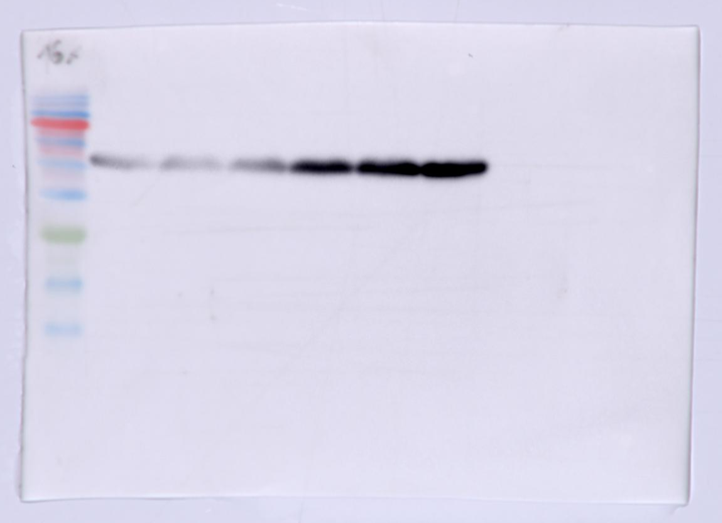

Supplement: Figure 2—source data 2. [file elife-99914-fig2-data2.zip › Figure 2-source data 2/TIMM50 Tubulin rep8.tif]

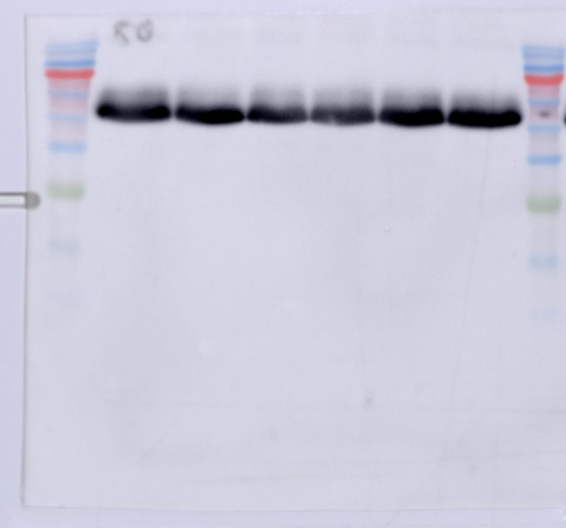

Supplement: Figure 2—source data 2. [file elife-99914-fig2-data2.zip › Figure 2-source data 2/TIMM50 Tubulin rep9.tif]

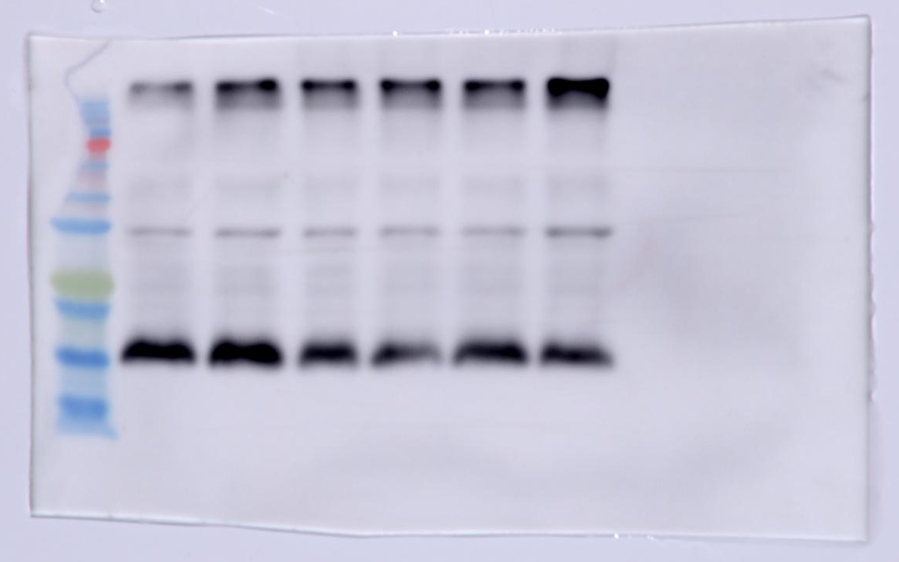

Supplement: Figure 2—source data 2. [file elife-99914-fig2-data2.zip › Figure 2-source data 2/TOMM20 rep1.tif]

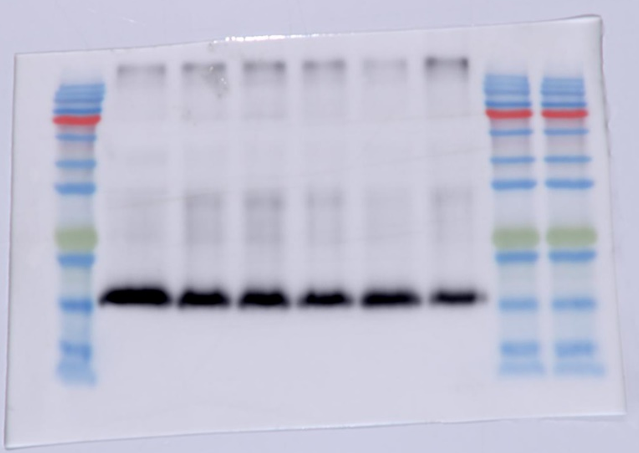

Supplement: Figure 2—source data 2. [file elife-99914-fig2-data2.zip › Figure 2-source data 2/TOMM20 rep2.tif]

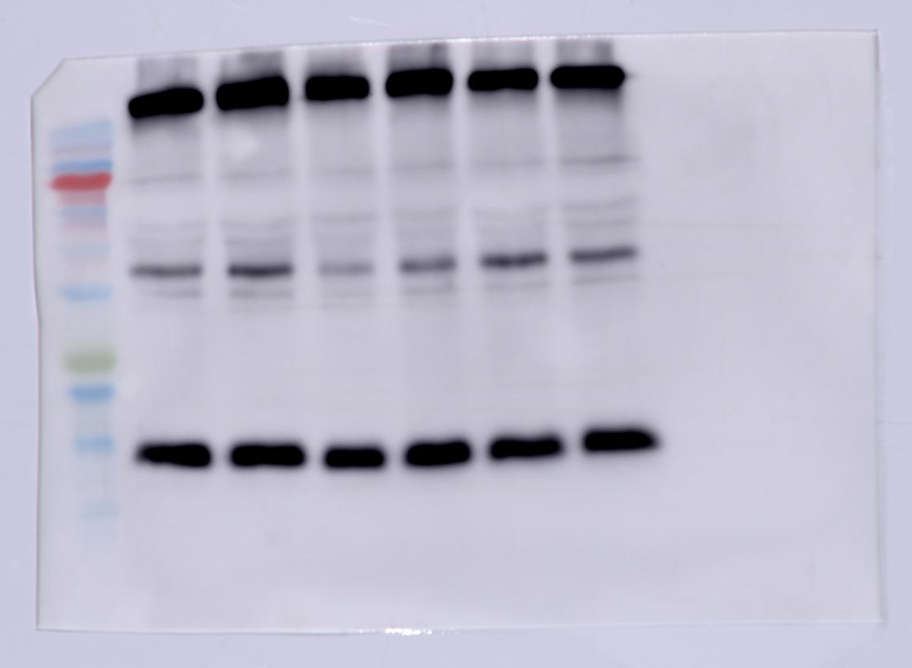

Supplement: Figure 2—source data 2. [file elife-99914-fig2-data2.zip › Figure 2-source data 2/TOMM20 rep3.tif]

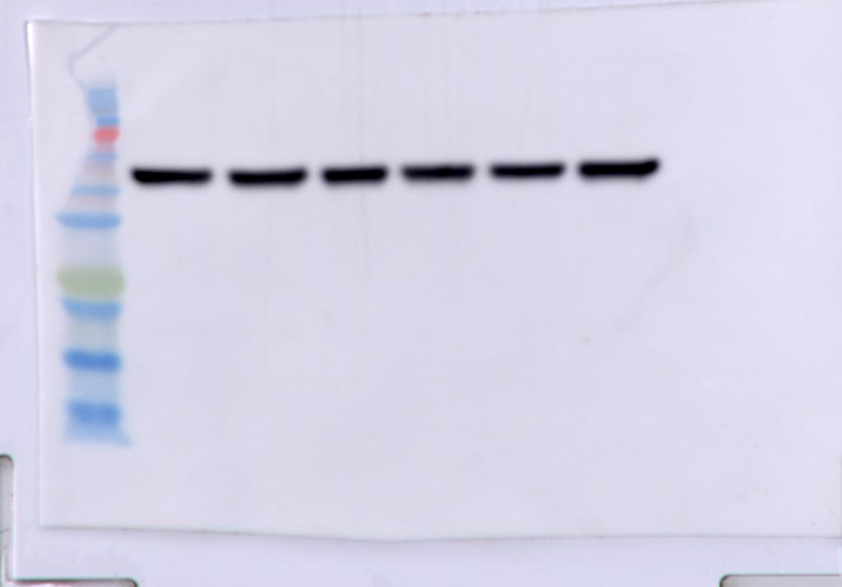

Supplement: Figure 2—source data 2. [file elife-99914-fig2-data2.zip › Figure 2-source data 2/TOMM20 Tubulin rep1.tif]

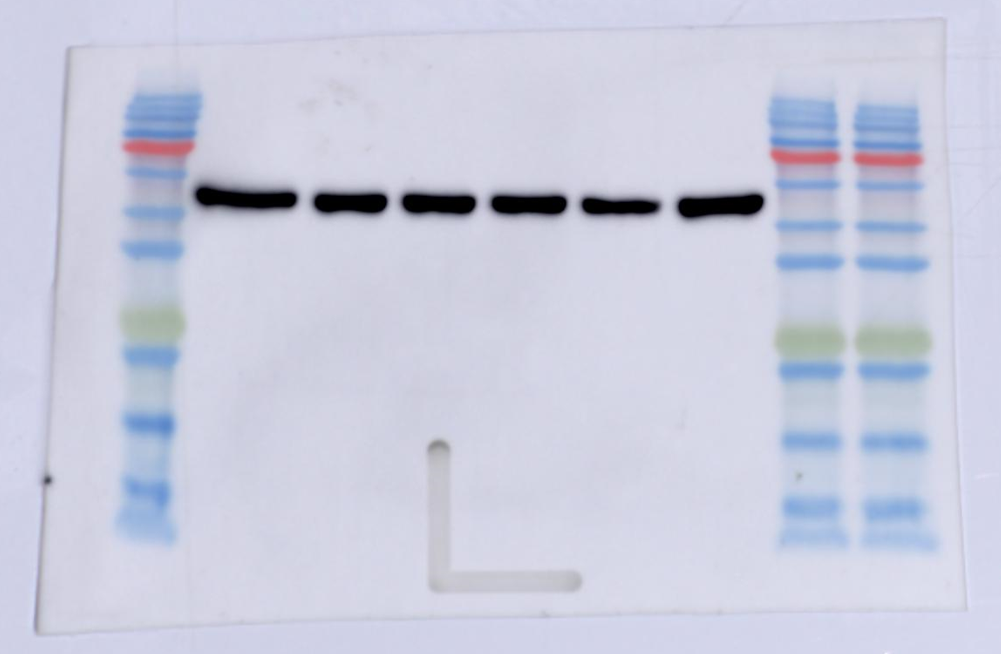

Supplement: Figure 2—source data 2. [file elife-99914-fig2-data2.zip › Figure 2-source data 2/TOMM20 Tubulin rep2.tif]

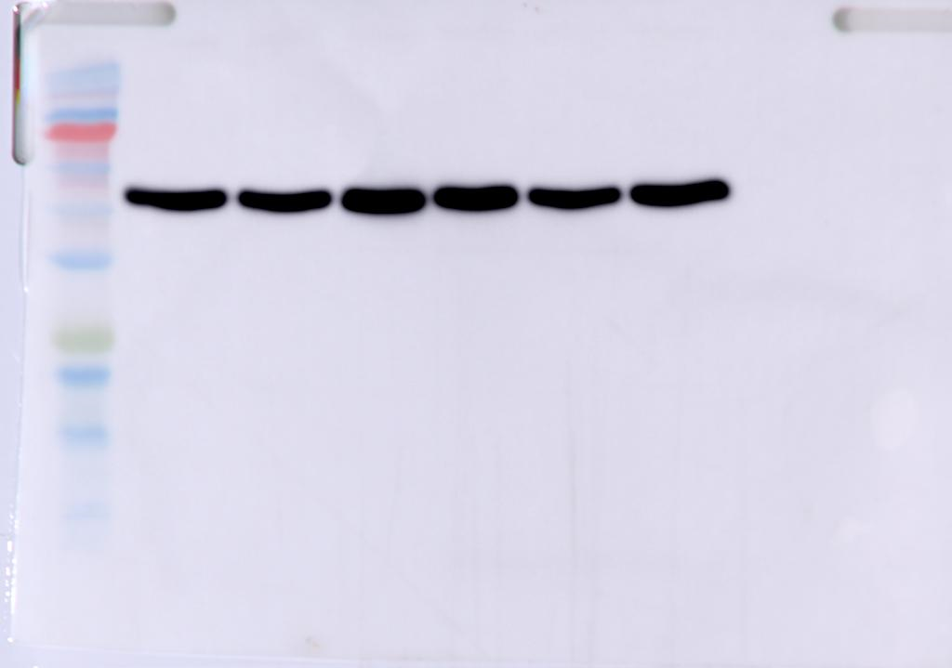

Supplement: Figure 2—source data 2. [file elife-99914-fig2-data2.zip › Figure 2-source data 2/TOMM20 Tubulin rep3.tif]

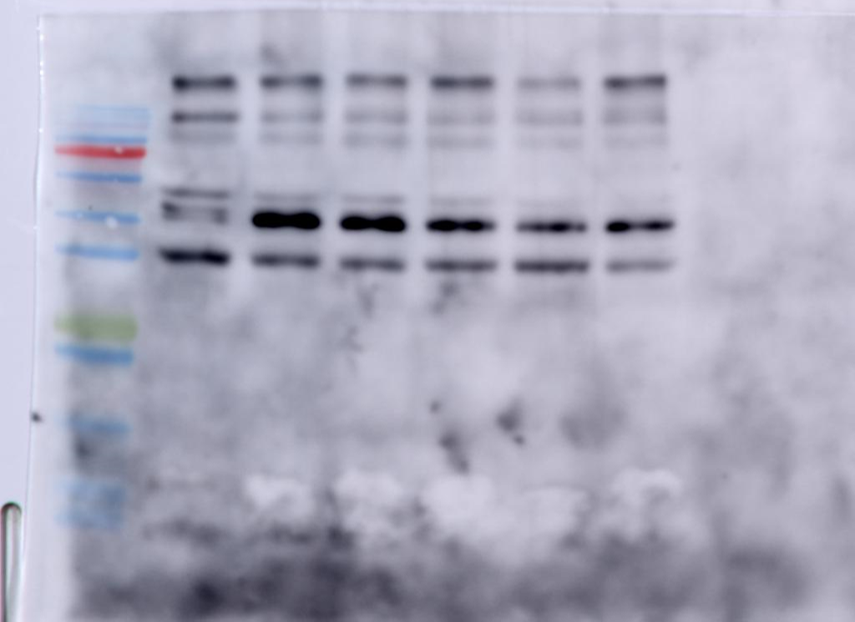

Supplement: Figure 2—source data 2. [file elife-99914-fig2-data2.zip › Figure 2-source data 2/TOMM40 rep1.tif]

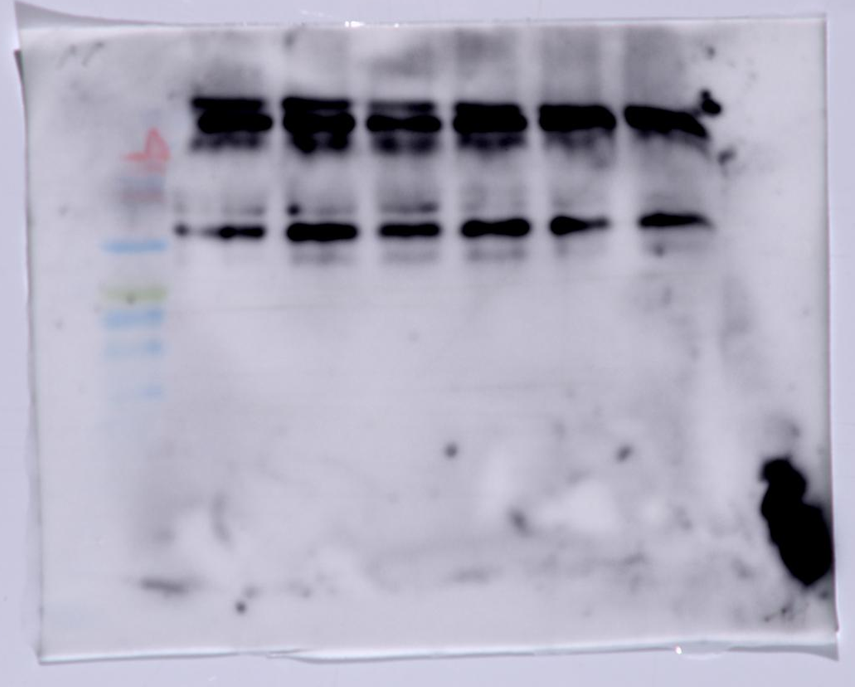

Supplement: Figure 2—source data 2. [file elife-99914-fig2-data2.zip › Figure 2-source data 2/TOMM40 rep2.tif]

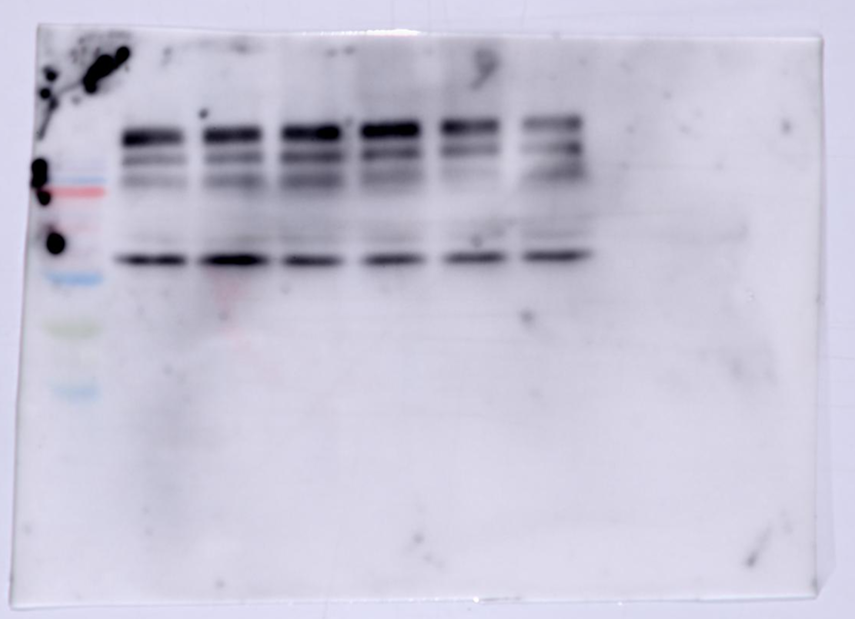

Supplement: Figure 2—source data 2. [file elife-99914-fig2-data2.zip › Figure 2-source data 2/TOMM40 rep3.tif]

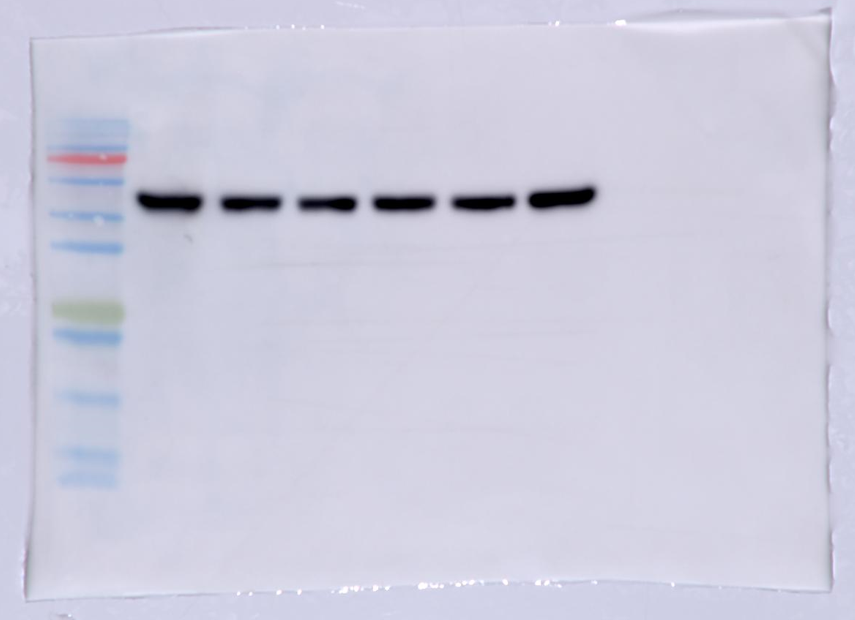

Supplement: Figure 2—source data 2. [file elife-99914-fig2-data2.zip › Figure 2-source data 2/TOMM40 Tubulin rep1.tif]

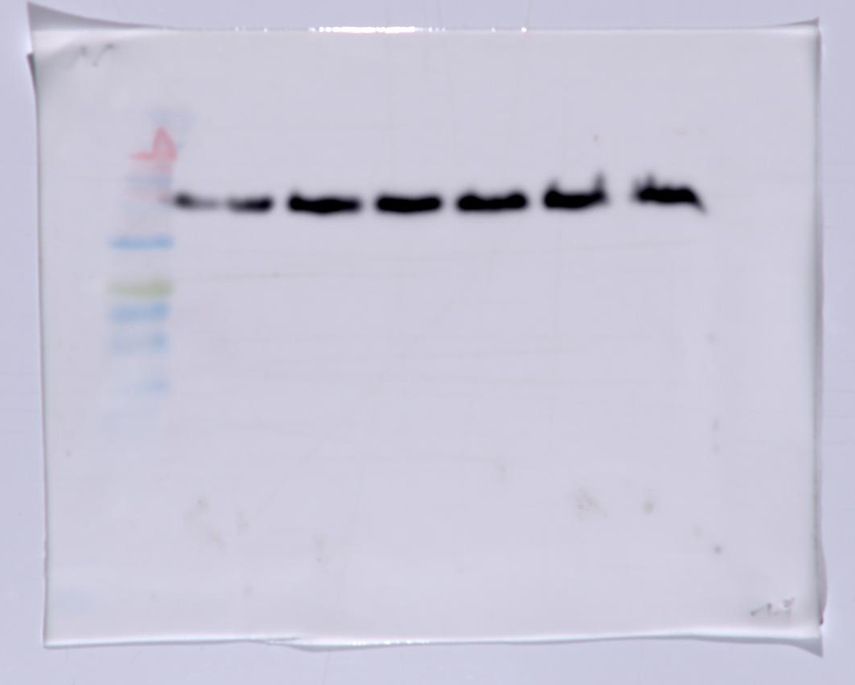

Supplement: Figure 2—source data 2. [file elife-99914-fig2-data2.zip › Figure 2-source data 2/TOMM40 Tubulin rep2.tif]

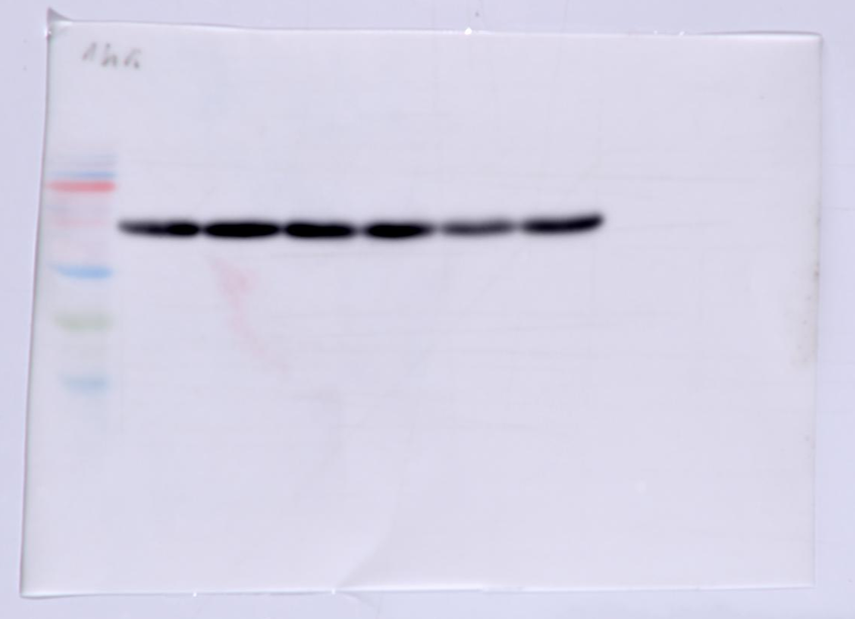

Supplement: Figure 2—source data 2. [file elife-99914-fig2-data2.zip › Figure 2-source data 2/TOMM40 Tubulin rep3.tif]

# ALDH2

(The running order is H.C / P1 / P2)

ALDH2 ▶

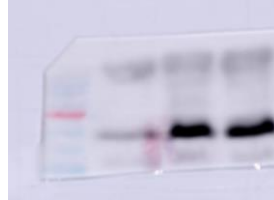

◀ GAPDH

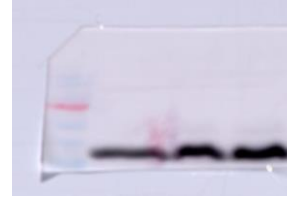

ALDH2 ▶

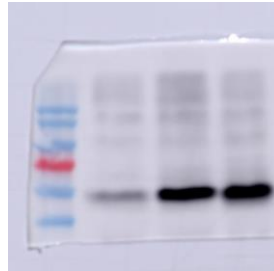

◀ Actin

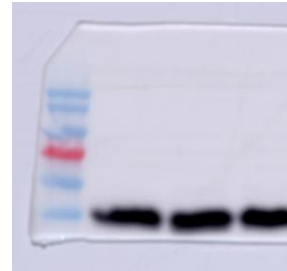

ALDH2 ▶

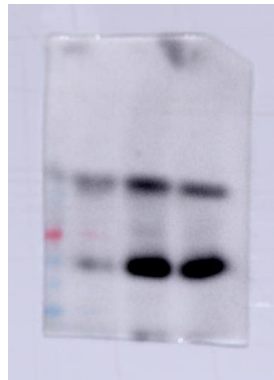

◀ Actin

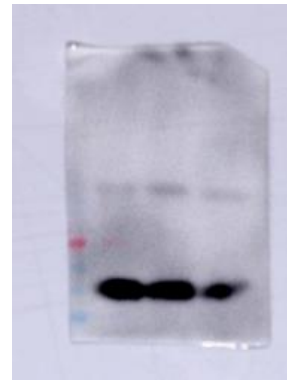

# GRSF1

(The running order is H.C / P1 / P2)

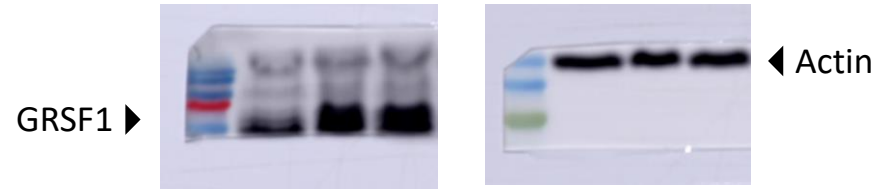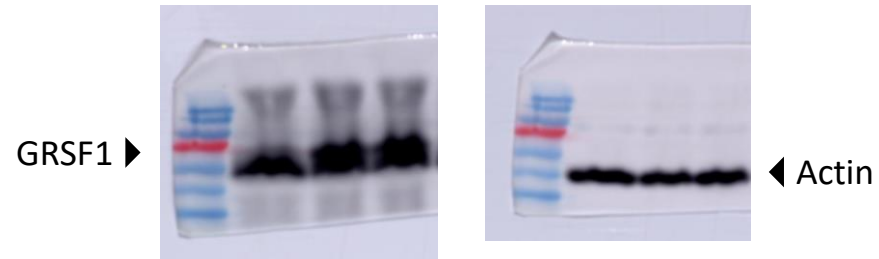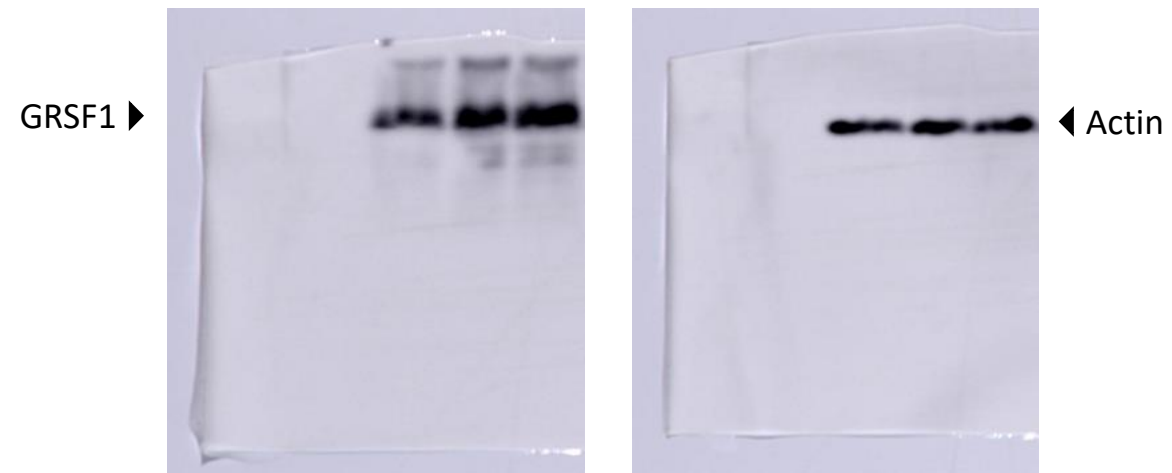

Supplement: Figure 3—figure supplement 2—source data 1. [file elife-99914-fig3-figsupp2-data1.zip › Figure 3-figure supplement 2-source data 1.pdf]

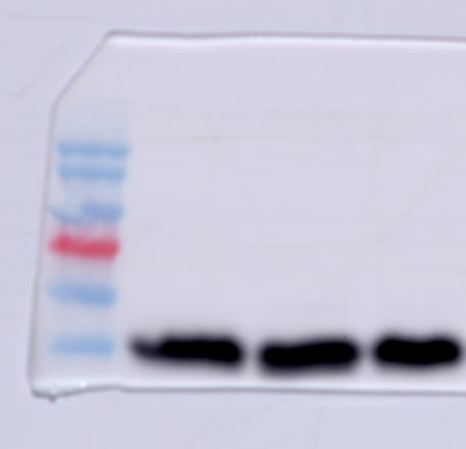

Supplement: Figure 3—figure supplement 2—source data 2. [file elife-99914-fig3-figsupp2-data2.zip › Figure 3-figure supplement 2-source data 2/ALDH2 Actin rep2.tif]

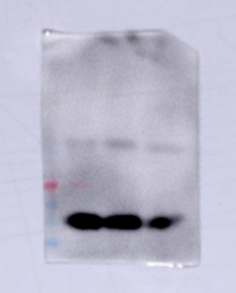

Supplement: Figure 3—figure supplement 2—source data 2. [file elife-99914-fig3-figsupp2-data2.zip › Figure 3-figure supplement 2-source data 2/ALDH2 Actin rep3.tif]

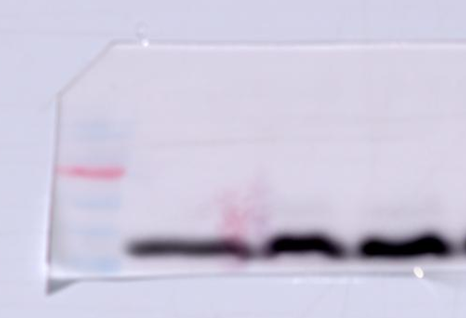

Supplement: Figure 3—figure supplement 2—source data 2. [file elife-99914-fig3-figsupp2-data2.zip › Figure 3-figure supplement 2-source data 2/ALDH2 GAPDH rep1.tif]

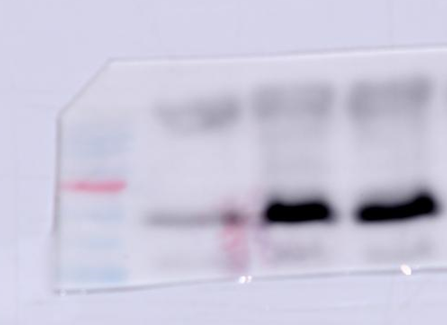

Supplement: Figure 3—figure supplement 2—source data 2. [file elife-99914-fig3-figsupp2-data2.zip › Figure 3-figure supplement 2-source data 2/ALDH2 rep1.tif]

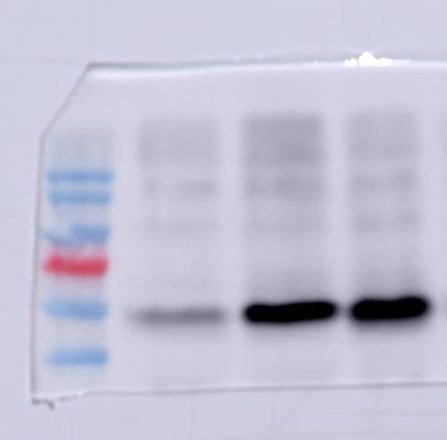

Supplement: Figure 3—figure supplement 2—source data 2. [file elife-99914-fig3-figsupp2-data2.zip › Figure 3-figure supplement 2-source data 2/ALDH2 rep2.tif]

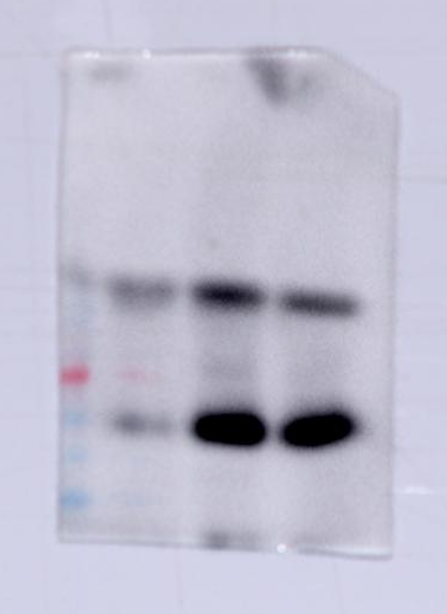

Supplement: Figure 3—figure supplement 2—source data 2. [file elife-99914-fig3-figsupp2-data2.zip › Figure 3-figure supplement 2-source data 2/ALDH2 rep3.tif]

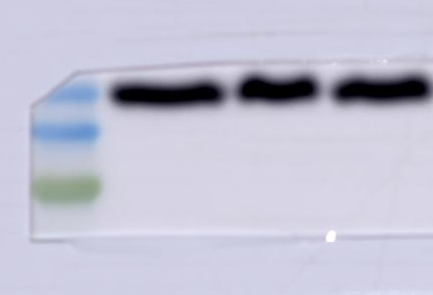

Supplement: Figure 3—figure supplement 2—source data 2. [file elife-99914-fig3-figsupp2-data2.zip › Figure 3-figure supplement 2-source data 2/GRSF1 Actin rep1.tif]

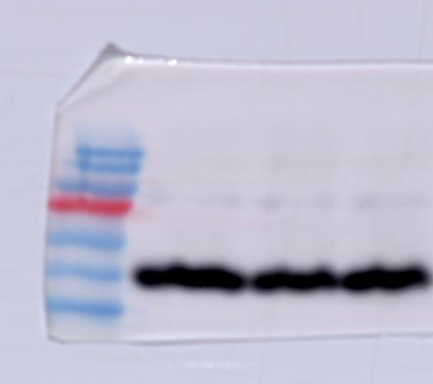

Supplement: Figure 3—figure supplement 2—source data 2. [file elife-99914-fig3-figsupp2-data2.zip › Figure 3-figure supplement 2-source data 2/GRSF1 Actin rep2.tif]

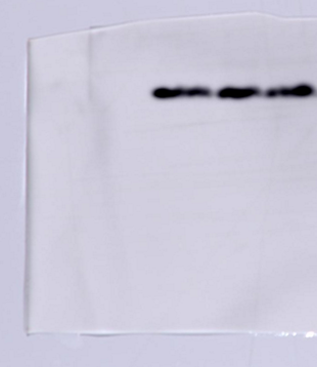

Supplement: Figure 3—figure supplement 2—source data 2. [file elife-99914-fig3-figsupp2-data2.zip › Figure 3-figure supplement 2-source data 2/GRSF1 Actin rep3.tif]

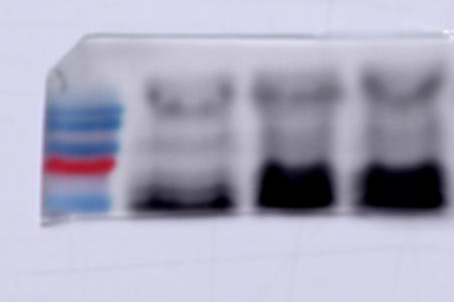

Supplement: Figure 3—figure supplement 2—source data 2. [file elife-99914-fig3-figsupp2-data2.zip › Figure 3-figure supplement 2-source data 2/GRSF1 rep1.tif]

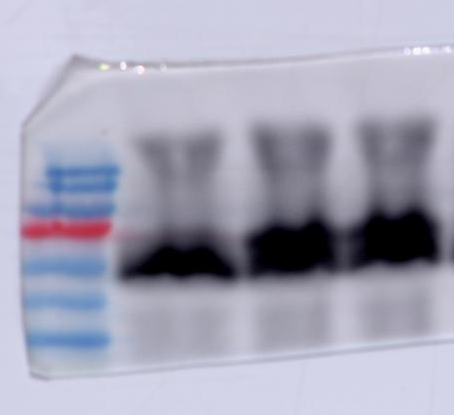

Supplement: Figure 3—figure supplement 2—source data 2. [file elife-99914-fig3-figsupp2-data2.zip › Figure 3-figure supplement 2-source data 2/GRSF1 rep2.tif]

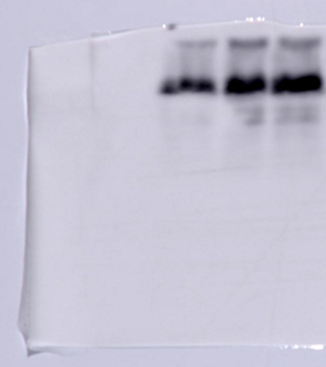

Supplement: Figure 3—figure supplement 2—source data 2. [file elife-99914-fig3-figsupp2-data2.zip › Figure 3-figure supplement 2-source data 2/GRSF1 rep3.tif]

# OXA1

(The running order is H.C / P1 / P2)

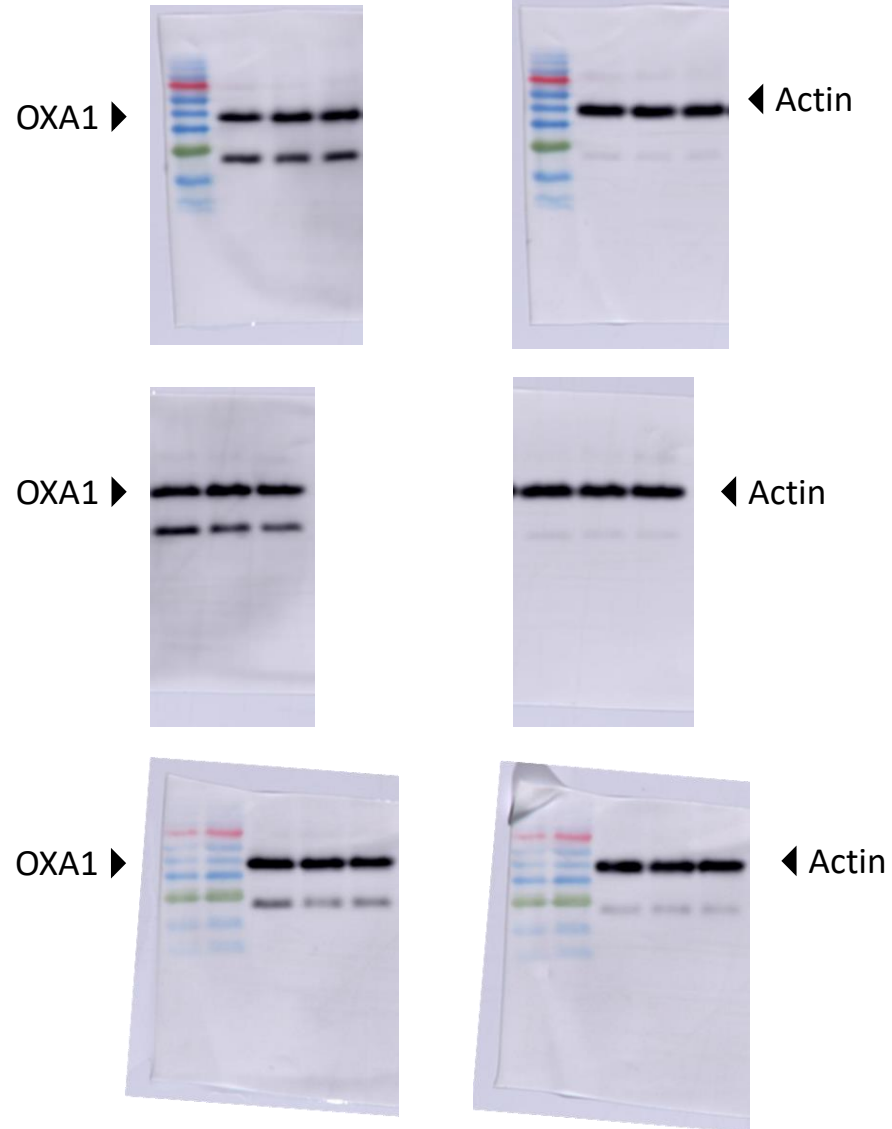

Supplement: Figure 3—figure supplement 3—source data 1. [file elife-99914-fig3-figsupp3-data1.zip › Figure 3-figure supplement 3-source data 1.pdf]

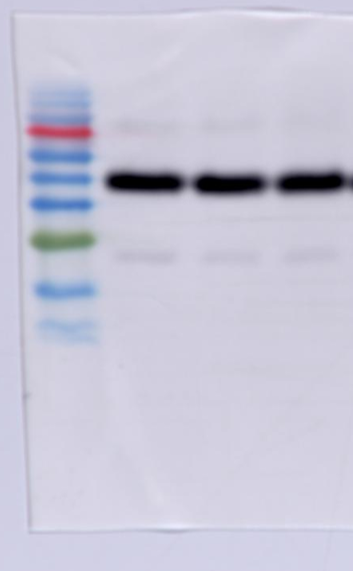

Supplement: Figure 3—figure supplement 3—source data 2. [file elife-99914-fig3-figsupp3-data2.zip › Figure 3-supplement 3-source data 2/OXA1 Actin rep1.tif]

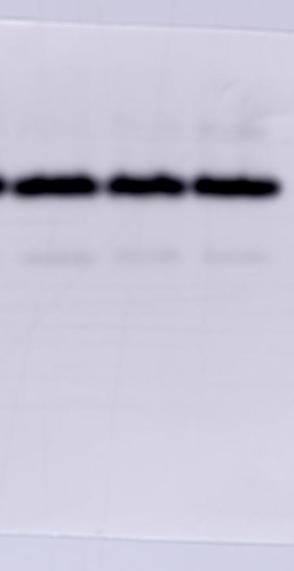

Supplement: Figure 3—figure supplement 3—source data 2. [file elife-99914-fig3-figsupp3-data2.zip › Figure 3-supplement 3-source data 2/OXA1 Actin rep2.tif]

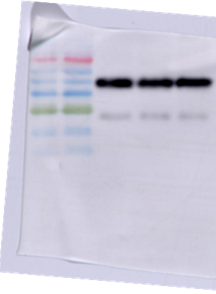

Supplement: Figure 3—figure supplement 3—source data 2. [file elife-99914-fig3-figsupp3-data2.zip › Figure 3-supplement 3-source data 2/OXA1 Actin rep3.tif]

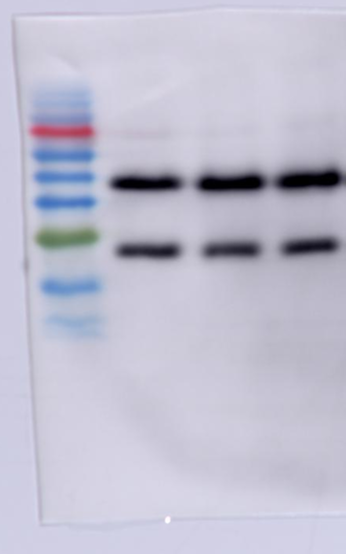

Supplement: Figure 3—figure supplement 3—source data 2. [file elife-99914-fig3-figsupp3-data2.zip › Figure 3-supplement 3-source data 2/OXA1 rep1.tif]

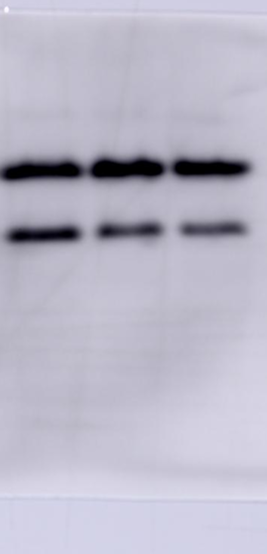

Supplement: Figure 3—figure supplement 3—source data 2. [file elife-99914-fig3-figsupp3-data2.zip › Figure 3-supplement 3-source data 2/OXA1 rep2.tif]

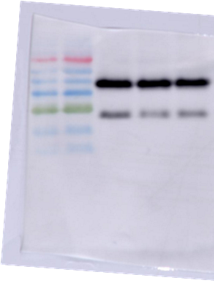

Supplement: Figure 3—figure supplement 3—source data 2. [file elife-99914-fig3-figsupp3-data2.zip › Figure 3-supplement 3-source data 2/OXA1 rep3.tif]

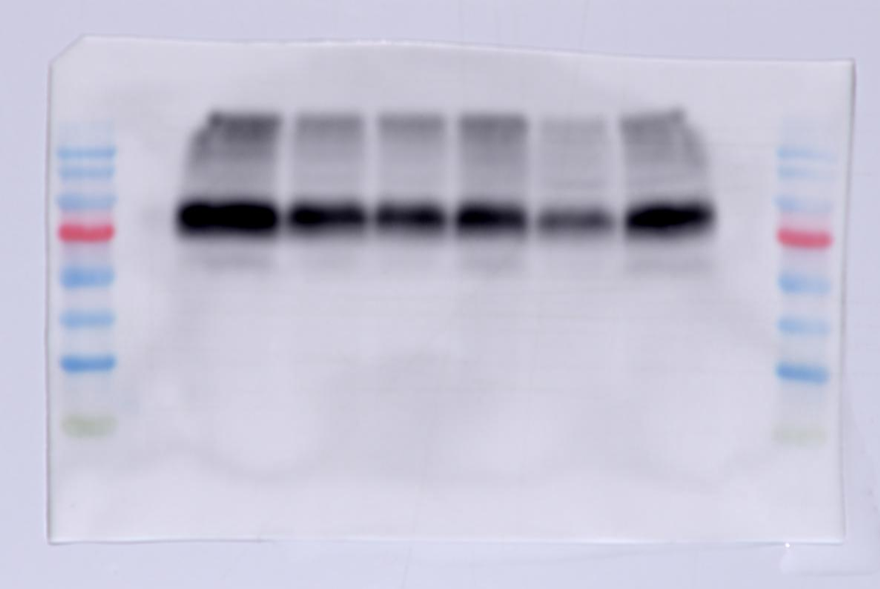

Supplement: Figure 7—figure supplement 2—source data 2. [file elife-99914-fig7-figsupp2-data2.zip › Figure 7-figure supplement 2-source data 2/KCNA2 rep1.tif]

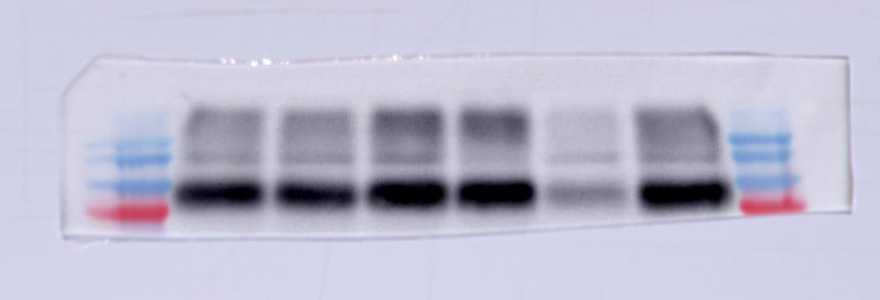

Supplement: Figure 7—figure supplement 2—source data 2. [file elife-99914-fig7-figsupp2-data2.zip › Figure 7-figure supplement 2-source data 2/KCNA2 rep2.tif]

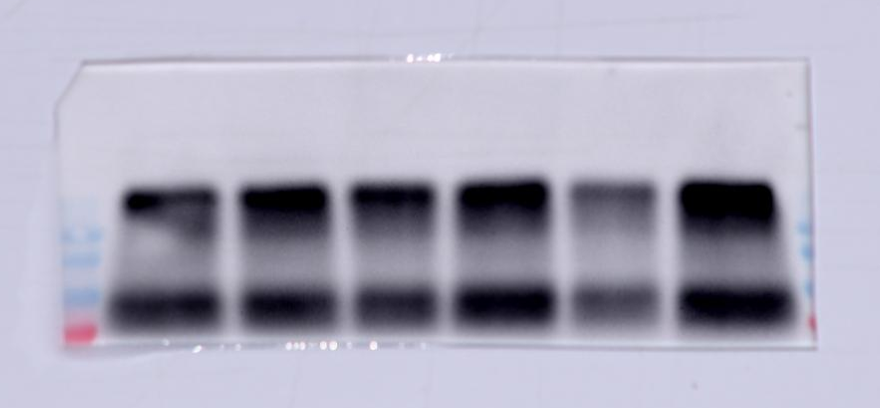

Supplement: Figure 7—figure supplement 2—source data 2. [file elife-99914-fig7-figsupp2-data2.zip › Figure 7-figure supplement 2-source data 2/KCNA2 rep3.tif]

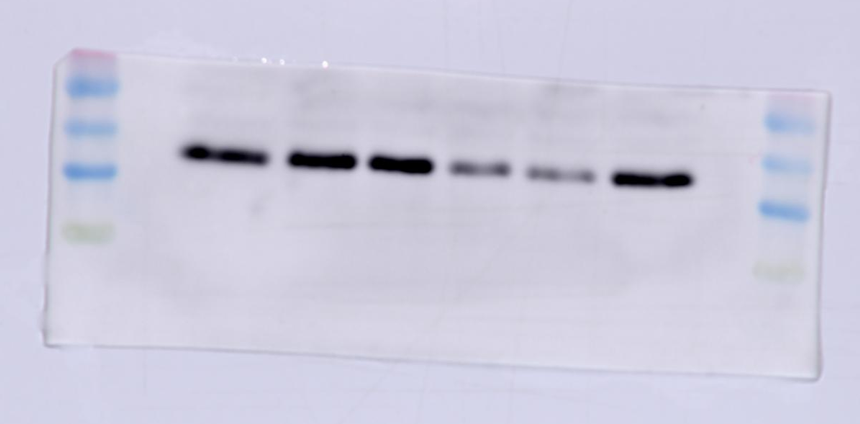

Supplement: Figure 7—figure supplement 2—source data 2. [file elife-99914-fig7-figsupp2-data2.zip › Figure 7-figure supplement 2-source data 2/KCNA2 TIMM50 rep1.tif]

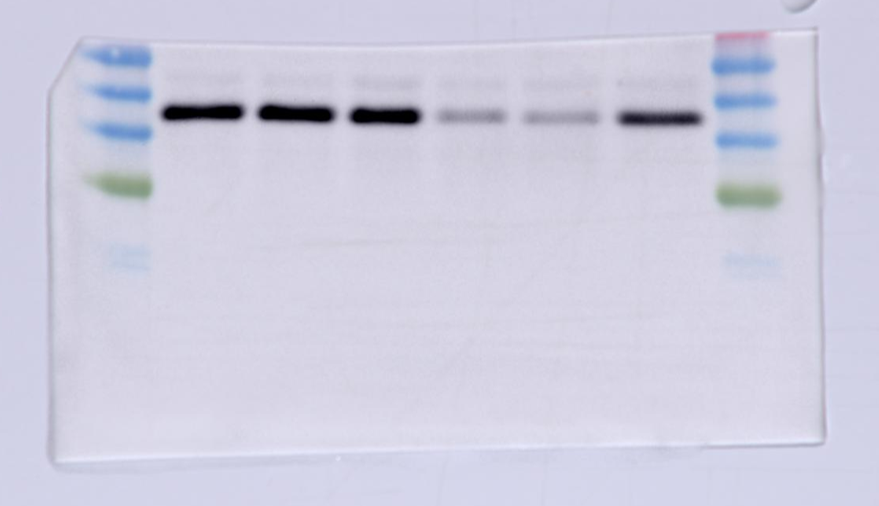

Supplement: Figure 7—figure supplement 2—source data 2. [file elife-99914-fig7-figsupp2-data2.zip › Figure 7-figure supplement 2-source data 2/KCNA2 TIMM50 rep2.tif]

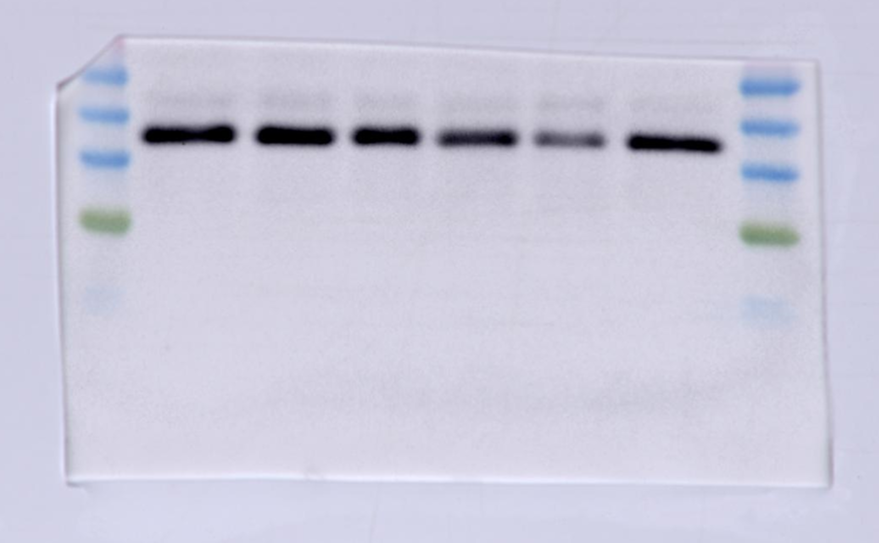

Supplement: Figure 7—figure supplement 2—source data 2. [file elife-99914-fig7-figsupp2-data2.zip › Figure 7-figure supplement 2-source data 2/KCNA2 TIMM50 rep3.tif]

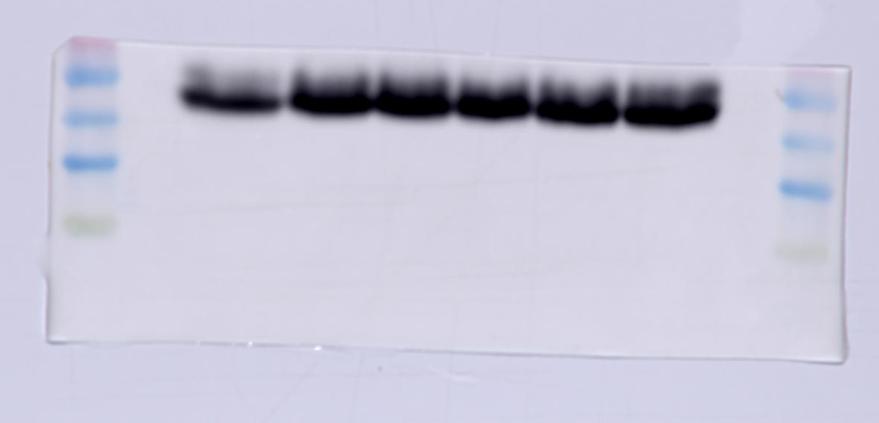

Supplement: Figure 7—figure supplement 2—source data 2. [file elife-99914-fig7-figsupp2-data2.zip › Figure 7-figure supplement 2-source data 2/KCNA2 Tubulin rep1.tif]

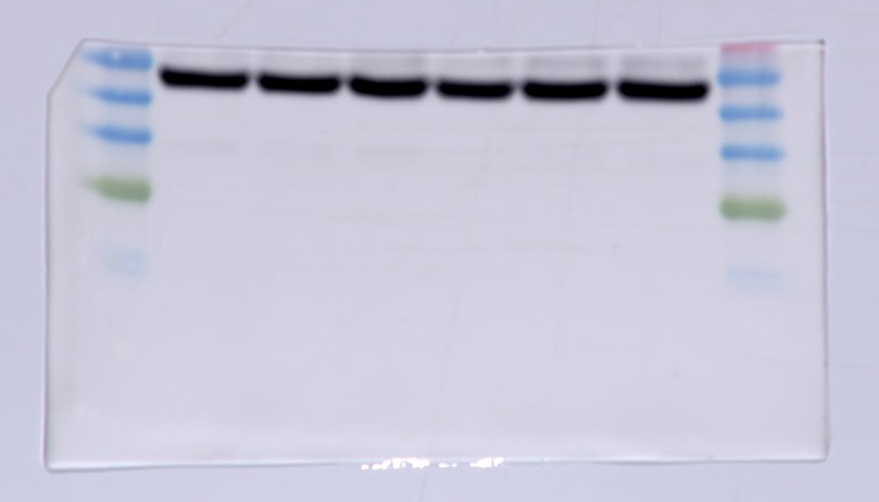

Supplement: Figure 7—figure supplement 2—source data 2. [file elife-99914-fig7-figsupp2-data2.zip › Figure 7-figure supplement 2-source data 2/KCNA2 Tubulin rep2.tif]

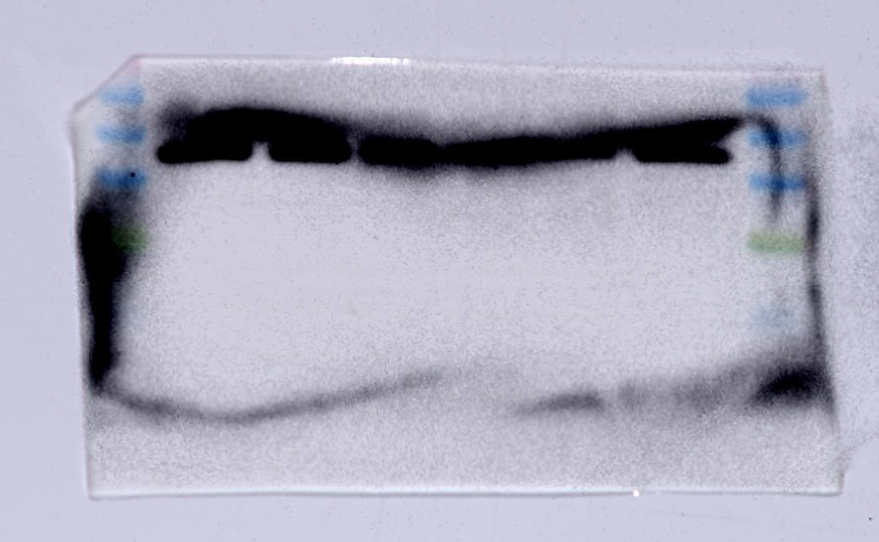

Supplement: Figure 7—figure supplement 2—source data 2. [file elife-99914-fig7-figsupp2-data2.zip › Figure 7-figure supplement 2-source data 2/KCNA2 Tubulin rep3.tif]

## Slide 1
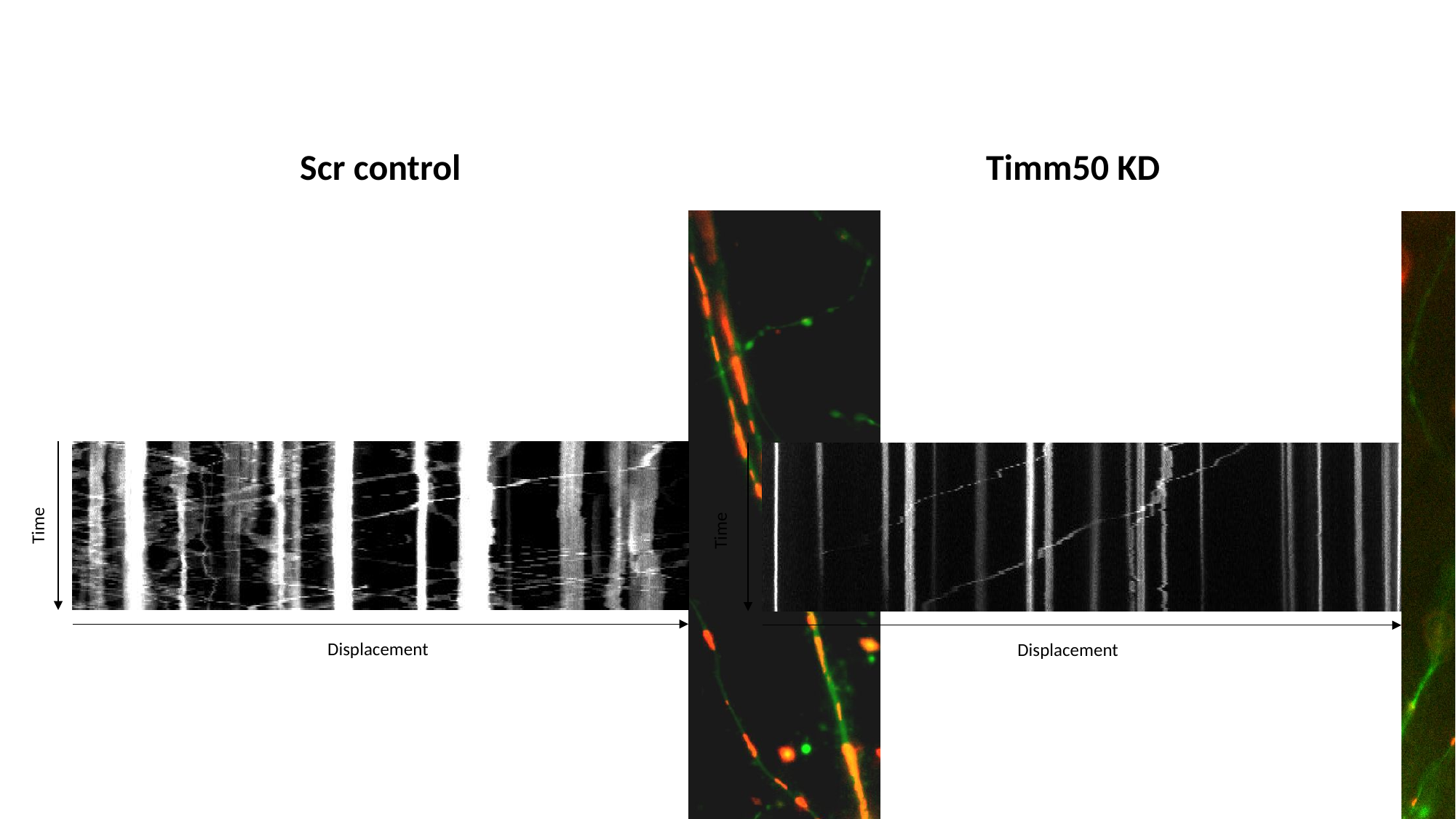

Scr control
Timm50 KD
Time
Time
Displacement
Displacement

Supplement: Supplementary file 3. — Scr control neurons and TIMM50 knockdown (KD) neurons co-transfected with the corresponding control/KD plasmid and mito-DsRed plasmid. The cells were imaged for 5 min with a 3 s interval between each image. The videos displayed are sped up by ~15x. [file elife-99914-supp3.zip › Video_1.pptx]
