# Supplementary material for: Biochemical and neurophysiological effects of deficiency of the mitochondrial import protein TIMM50
Source: eLife. 2024 Dec 16;13:RP99914. doi: 10.7554/eLife.99914 (PMC11649234; doi:10.7554/eLife.99914)

KCNA2

(Running order is Untreated / pLL3.7 control / Scr control / Sh1 / Sh2 / Sh3)

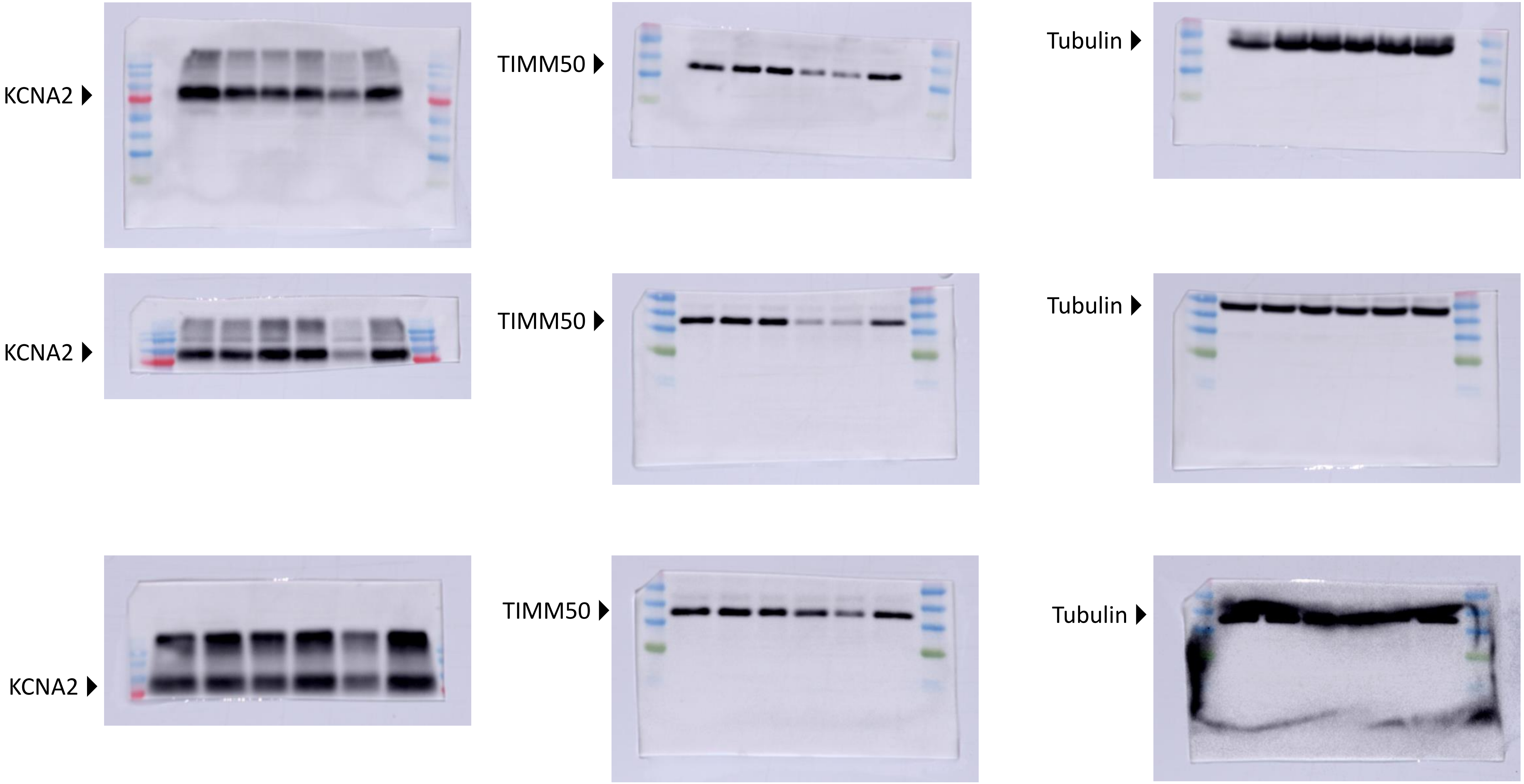

Supplement: Figure 7—figure supplement 2—source data 1. [file elife-99914-fig7-figsupp2-data1.zip › Figure 7-figure supplement 2-source data 1.pdf]
